# Short-Range Effects in the Special Pair of Photosystem II Reaction Centers: The Nonconservative Nature of Circular Dichroism

**DOI:** 10.1021/acs.jpclett.3c02693

**Published:** 2023-12-20

**Authors:** Felix G. Gemeinhardt, Yigal Lahav, Igor Schapiro, Dror Noy, Frank Müh, Dominik Lindorfer, Thomas Renger

**Affiliations:** † Institut für Theoretische Physik, Johannes Kepler Universität Linz, Altenberger Strasse 69, 4040 Linz, Austria; ‡ Fritz Haber Center for Molecular Dynamics Research, Institute of Chemistry, Hebrew University of Jerusalem, 9190401 Jerusalem, Israel; ¶ MIGAL - Galilee Research Institute, S. Industrial Zone, 1101602 Kiryat Shmona, Israel; § Faculty of Sciences and Technology, Tel-Hai Academic College, 1220800 Upper Galilee, Israel

## Abstract

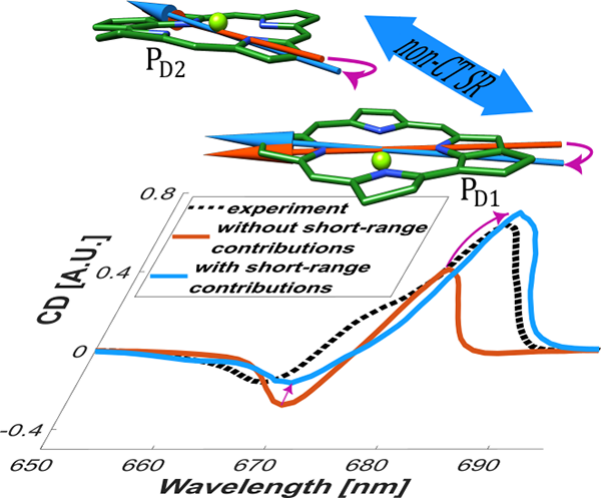

Photosystem II reaction centers extract electrons from water, providing the basis of oxygenic life on earth. Among the light-sensitive pigments of the reaction center, a central chlorophyll *a* dimer, known as the special pair, so far has escaped a complete theoretical characterization of its excited state properties. The close proximity of the special pair pigments gives rise to short-range effects that comprise a coupling between local and charge transfer (CT) excited states as well as other intermolecular quantum effects. Using a multiscale simulation and a diabatization technique, we show that the coupling to CT states is responsible for 45% of the excitonic coupling in the special pair. The other short-range effects cause a nonconservative nature of the circular dichroism spectrum of the reaction center by effectively rotating the electric transition dipole moments of the special pair pigments inverting and strongly enhancing their intrinsic rotational strength.

In photosynthesis, light is absorbed by light-harvesting antennae, and its energy is transferred to a reaction center (RC), where it drives transmembrane electron transfer.^1,2^ Whereas there are different types of antennae, which have been adapted to a particular environmental condition or a certain light-harvesting strategy,^3^ all known photosynthetic reaction centers share a very similar structure.^4^ There is a central chlorophyll (Chl) or bacteriochlorophyll (BChl) dimer, known as the special pair, and two quasisymmetric branches of cofactors spanning the photosynthetic membrane (Figure 1).

**Figure 1:**
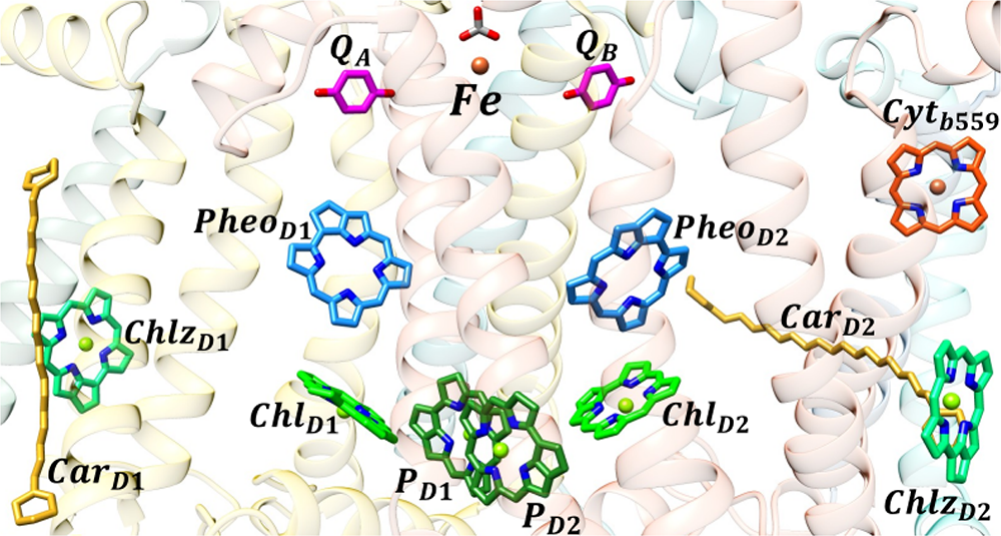
Cofactors of the reaction center of photosystem II (PDB ID: 3WU2), viewed parallel to the photosynthetic membrane. Two quasi-symmetric branches span the membrane with the P_D1_–P_D2_ dimer, termed special pair, in the bottom center and two quinones Q_A_ and Q_B_ at the top. Chlorophyll (green), pheophytin (blue) and β-carotene (yellow) pigments are included in the calculation of optical spectra.

In the case of the type II RC of purple bacteria (bRC), the primary electron donor is the special pair containing two BChl pigments. Due to their close proximity, besides long-range (LR) Coulomb interaction, also short-range (SR) interactions, resulting from wave function overlap, become significant. These SR interactions give rise to a coupling between local excited (LE) and charge transfer (CT) states,^5,6^ as well as additional effects,^7^ which will be summarized as non-CT-SR contributions. Theoretical studies on simple model systems investigated the relative importance of CT-coupling and the non-CT-SR contributions due to Dexter exchange interaction^8^ and orbital penetration^9^ to the excitonic coupling. The CT coupling effects were reported to be large compared to non-CT-SR contributions.^10,11^ Concerning the latter, except for the case of orthogonal donor and acceptor orbitals,^12^ orbital penetration effects were reported to dominate Dexter electron exchange.^9^ In the present work, we will use a diabatization technique to identify the CT-contributions, but we will not be able to discriminate between different non-CT-SR effects.

In the special pair of bRC, SR effects lead to a strong red shift with respect to the transition energies of the isolated special pair pigments,^5,6,13−16^ bringing it in near resonance to the low-energy exciton states of the core light-harvesting complex LH1 for efficient energy transfer.^17^

In the related type II RC of cyanobacteria and higher plants (Figure 1), Chl *a* and pheophytin (Pheo) *a* molecules, respectively, replace the BChl and bacteriopheophytin pigments of bRC. The former absorb blue-shifted with respect to their counterparts in bRC and exhibit a higher oxidation potential, which is needed for the splitting of water in photosystem II (PSII). Electrostatic pigment–protein coupling has been identified as a major source in oxidation potential tuning in PSII-RCs.^4,18^ Another factor contributing to the large oxidation potential, most likely, is a decrease of the wave function overlap in the special pair. There is a mutual tilt of the special pair pigments in PSII with respect to those in bRC.^19^ This tilt is assumed to increase the oxidation potential to accept an electron from water.^4,20^ The decreased wave function overlap has dramatic consequences for the optical properties of PSII-RC. Whereas in bRC, the low-energy band is dominated by the special pair,^21^ in the case of PSII-RCs all bands are strongly overlapping.^19^ This spectral congestion problem has hampered our interpretation of optical experiments and, thereby, our understanding of the primary processes in the PSII-RC.

Valuable information about the energetics of excited states in PSII-RCs has been obtained from optical difference spectra. In these experiments, single amino acids in the neighborhood of certain RC pigments have been varied,^22,23^ or whole pigments have been exchanged,^24^ removed^25^ or reduced,^26^ or the RC was converted into a triplet^22−24,27^ or charge-separated state.^28,29^ In combination with a theoretical description of the optical spectra,^30−38^ we have now a better understanding of the energetics of excited states in PSII-RCs.

An open point in the calculation of the optical spectra of PSII-RCs concerns the quantification of SR effects in the special pair. Raszewski et al.^33^ inferred an excitonic coupling of 140–170 cm^–1^ in the special pair from fits of optical spectra. This value is significantly larger than the LR Coulomb contribution they calculated based on the crystal structure. Preliminary calculations,^20^ using a premature diabatization technique,^16^ revealed a 100 cm^–1^ SR contribution, but with an opposite sign than the LR part. The simple technique will be replaced in the present work by a sophisticated diabatization^39^ that explicitly includes CT states and uses a special pair geometry obtained from hybrid quantum mechanics/molecular mechanics (QM/MM) simulations.

Circular dichroism (CD) spectra represent a valuable means to assess the validity of exciton Hamiltonians, because the spectra of pigment–protein complexes are usually dominated by the chirality of the exciton wave functions, rather than the intrinsic chiralities of the individual pigments. Therefore, CD spectra are exceptionally sensitive to relative orientations and distances between pigments.^40,41^ Chl *a* and related pigments exhibit a relatively large spectral gap between the first excited state (Q_y_) and the higher excited states (Q_x_, B_y_, B_x_, N_x+xy_, etc.). Therefore, the low-energy region of the CD spectrum of photosynthetic pigment–protein complexes are often described by just accounting for the Q_y_ states of the pigments.^35,36,42,43^ However, the CD spectrum of PSII-RCs in the Q_y_ region is highly nonconservative with an experimentally observed ratio of positive and negative contributions of 3.6,^24,44^ instead of the ratio 1.0 predicted by standard exciton theory including the Q_y_ states.

The nonconservativities of the Q_y_ region of the CD spectra of the LH1 light-harvesting complex of purple bacteria^45^ and of the CP29 light-harvesting complex of PSII^46^ have been explained by taking into account the interpigment excitonic couplings between Q_y_ transitions of (bacterio-) chlorophyll pigments and high-energy transitions of (bacterio-) chlorophylls and carotenoids.

Lindorfer et al.^47^ recently investigated these couplings in a study of the nonconservative nature of the CD spectrum of PSII-RCs. In order to explain the experimental nonconservativity, they had to assume that the intraspecial pair excitonic couplings between Q_y_ and higher excited states, calculated from the Coulomb coupling of transition densities, are strongly enhanced. This result gave rise to the hypothesis that the coupling between LE and CT states^10,11,16^ is responsible for the enhancement and, thereby, for the nonconservativity of the CD spectrum. A main goal of the present work has been to investigate this hypothesis by explicitly taking into account the coupling between the LE and CT states. As will be shown here, besides coupling to CT states, there are other more dominant SR effects at play that determine the nonconservative nature of the CD spectrum.

In order to cope with the tradeoff between accuracy and computational feasibility, we divide the PSII-RC into parts, which are treated according to the exciton Hamiltonian with parameters taken from our earlier work,^36,47^ and the special pair dimer which is described using quantum chemistry methods. Starting from the crystal structure provided by Umena et al.,^48^ only the D1 and D2 proteins together with PsbI and cytochrome b559 were kept (for a review of protein subunits and available crystal structures, see ref^49^). The most probable protonation pattern of the PSII-RC (with the water-oxidizing Mn_4_CaO_5_ complex removed) has been computed with Poisson–Boltzmann type calculations.^36,50^ The resulting atomic structure was embedded in a DOPC membrane and served as an input for molecular dynamics (MD) simulations. A schematic overview of the applied methodological procedure is given in the SI, Section 1, and further details are provided in the SI, Sections 2 and 3.

A representative structure of the special pair dimer is selected from the MD simulation as the snapshot, which shows the least root-mean-square deviation (RMSD) compared to the average backbone configuration of the protein (cf. SI, Section 4). We will use this representative structure and an additional 110 MD snapshots (after a QM/MM geometry optimization) for the quantum chemical calculations of SR effects in the special pair. The calculations on the snapshots are needed to obtain disorder parameters for the calculation of inhomogeneous broadening of the spectra and to validate the short-range contributions obtained for the respresentative structure. The atomic coordinates of the representative structure are provided in the SI (Section 4.2).

Using an electrostatic embedding, modeling the environment of the dimer as a set of atomic partial charges, 30 excited states of the special pair have been computed at the TDDFT level of theory using the LR corrected functional ωB97X-D3(BJ) together with the def2-SVP basis set. The Multi-FED-FCD diabatization method developed by Nottoli et al.^39^ has been utilized to find an adiabatic-to-diabatic transformation from dimer excited states to LE and CT states. The scheme combines the Fragment Charge Difference^51^ and the Fragment Excitation Difference methods.^52^ Based on molecular properties of the dimer system (excitation energies, transition densities, molecular orbitals), a unitary transformation from the basis of adiabatic dimer eigenstates to a basis of diabatic CT and LE states is computed in a multistep process, as described in detail in the SI (Section 5.1).

After the diabatization, we obtain a special pair Hamiltonian that contains local excitation energies *E*_*ma*_^diab^ of the *a*th diabatic excited state localized at site *m* = P_D1_, P_D2_ and energies *E*_*k*CT_^diab^ of intra special pair CT states. Additionally, we get electronic couplings *V*_*ma*,*nb*_^diab^, *V*_*ma*,*k*CT_^diab^ and *V*_*k*CT,*l*CT_^diab^ between the different diabatic states. We further obtain electric transition dipole moments **μ**_*ma*_^diab^ and **μ**_*k*CT_^diab^ of LE and CT states, respectively, and corresponding magnetic transition dipole moments ***m***_*ma*_^diab^ and ***m***_*k*CT_^diab^.

The lowest energy CT states are about 10000 cm^–1^ above the Q_y_ states of P_D1_ and P_D2_ and the largest couplings  between the Q_y_ and the CT states are in the order of 1000 cm^–1^ (Table S4), justifying the perturbation theory that will be used below to treat the mixing of Q_y_ and CT states. From an analysis of the transition density matrix of the special pair, the first two adiabatic states can be characterized as delocalized over the Q_y_ states of P_D1_ and P_D2_ with a 3–5% admixture of CT states. The CT states obtained from the diabatization have an actual CT chararacter larger than 82% (90% on average). The remaining small percentage of LE character results in nonzero but small transition dipole moments of the diabatic CT states, which, however, have no critical effect on the results, as will be shown below. Further details of the diabatic Hamiltonian parameters are reported and discussed in the SI (Section 5.2).

The above quantities are incorporated into an existing Frenkel exciton Hamiltonian of the PSII-RC,^47^ where we use perturbation theory to include the coupling between LE Q_y_ states and CT states of the special pair. The resulting Hamiltonian reads
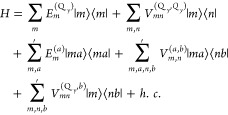
1

Here, |*m*⟩ = |*m*Q_y_⟩ describes an excited state of the RC in which the *m*th pigment is in its first excited state and all other pigments are in their electronic ground state, and in |*ma*⟩ the *m*th pigment is in a higher excited state *a*, with local excitation energies  and *E*_*m*_^(*a*)^, respectively. The , *V*_*m*,*n*_^(*a*,*b*)^ and *V*_*mn*_^(*Q_y_,_b_*)^ denote the excitonic couplings between Q_y_ transitions, between high-energy transitions, and between Q_y_ and high-energy transitions, respectively. The prime at the sums in the second and third lines of eq 1 indicates that *a* = Q_y_ and *b* = Q_y_ are not included. The five lowest LE states obtained for P_D1_ and P_D2_ from the diabatization are included in the Hamiltonian in eq 1. The first two diabatic LE states of the special pair have been identified as Q_y_ states, based on the fact that their transition dipole moments are oriented along the *y*-axis of the P_D1_ or P_D2_ pigment.

The various local excitation energies and excitonic couplings in eq 1 that do not involve the special pair pigments P_D1_ and P_D2_ are taken from our previous work.^36,47^ As before, the Q_y_, B_y_, B_x_, and N_x+xy_ transitions are included for the Chl *a* and Pheo *a* pigments and the *S*_0_ → *S*_2_ transition for the β-carotene (Car) pigments.^46,47^ We used the Poisson-TrEsp method^53−55^ for the calculation of the LR part of the excitonic coupling between LE states of the special pair and the LE states of the remaining RC pigments, as described in the SI (Section 6.1.1).

The local excitation energies of the Q_y_ transitions  and the excitonic coupling between the Q_y_ transition of one special pair pigment and the different electronic excitations of the other are derived from the diabatization described above. Here, we use second-order perturbation theory for the coupling of local excitations to intradimer CT states. The resulting Q_y_ transition energies are given by
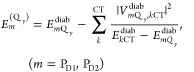
2

By comparing the diabatic energies  with those obtained for the isolated monomers, non-CT-SR contributions to the site energy shifts can be inferred, whereas the second contribution on the r.h.s. of eq 2 reveals the effect of couplings between LE and CT states. This analysis was performed for the representative structure. We have checked the reliability of this structure by comparing energies and couplings with those obtained from an average over 110 geometry-optimized MD snapshots. Interestingly, non-CT-SR effects lead to a 57 cm^–1^ blue-shift of the site energies (averaged over P_D1_ and P_D2_, Table S5) and the coupling to CT states results in a 158 cm^–1^ red shift (164 cm^–1^ average over the 110 MD snapshots, Table S8), such that the overall SR effect on the site energies is rather small. In particular, it is an order of magnitude smaller than for the special pair of the bacterial RC,^5,6,16^ which has an increased wave function overlap, as discussed above. This result is in agreement with the finding that the energy sink in PSII-RCs is not the special pair but the accessory chlorophyll of the D1-branch, Chl_D1_.^31,36−38^ In our earlier electrostatic calculations of site energies,^36^ SR related (as well as inductive and dispersive) site energy shifts have been treated implicitly by a refinement fit of site energies, based on the comparison of calculated and experimental spectra. The present calculations show that the SR effects are indeed small. Further details of the site energy calculations of the special pair, including also the electrostatic shifts by the protein environment, are given in the SI (Section 5.2).

The CT state mediated excitonic coupling between the Q_y_ transition of one and the *a*th diabatic higher energy LE state of the other special pair pigment is obtained in a similar way as the site energy (eq 2)
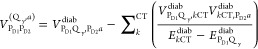
3

Whereas eq 2 is the standard perturbation theory, eq 3 needs some justification. The case *a* = Q_y_ in eq 3 is more accurate than *a* ≠ Q_y_, since in the latter case an upper bound is used for the energy gap, as explained in the SI (Section 5.1).

LR Coulomb interaction of transition densities and non-CT-SR effects are included in the coupling  between Q_y_ states, for which we obtain a value of 137 cm^–1^ from the diabatization (133 cm^–1^ average over the 110 MD snapshots). The LR contribution is obtained from the Coulomb coupling between monomer transition densities and amounts to 149 cm^–1^. Hence, we see that the non-CT-SR contribution is small (−12 cm^–1^) and opposite in sign compared with the Coulomb contribution. The Coulomb part can be rescaled in order to correct for uncertainties in the quantum chemical transition dipole moments and for screening effects by the optical polarizability of the environment. We use experimental data for the dipole strengths^56^ as well as Poisson-TrEsp electrostatic calculations of screening effects.^54,55,57^ These corrections result in a LR Coulomb contribution of 107 cm^–1^, as explained in detail in the SI (Section 5.2).

The second contribution on the rhs of eq 3 for *a* = Q_y_ arises from superexchange type couplings of Q_y_ states via CT states in the special pair, which result in a coupling contribution of 98 cm^–1^ (110 cm^–1^ average over 110 MD snapshots). Adding to the latter value the non-CT-SR contribution (−12 cm^–1^) and the rescaled LR contribution (107 cm^–1^) results in an overall excitonic coupling of
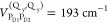
4which is close to the range of 140–170 cm^–1^ obtained indirectly from fits of optical spectra.^33^ In summary, about 45% of the excitonic coupling between Q_y_ transitions of the special pair is due to coupling to CT states, and non-CT-SR effects are small, in agreement with earlier calculations on simple model systems.^10,11^ Note that the fit of optical spectra suggested an optimal value of 150 cm^–1^ for ,^33^ which is also suitable for the description of the nonconservativity ratio of the CD spectrum, discussed below.

As for , we find for the excitonic couplings  and  (eq 3) between Q_y_ and high-energy states that the non-CT-SR contributions are small compared to the LR and the CT-state mediated couplings (SI, Tables S6 and S7). Interestingly, the relative CT coupling contributions are smaller for the high-energy than for the Q_y_ transition, in contrast to our earlier hypothesis,^47^ that due to the larger wave function overlap of higher excited states, the enhancement of excitonic couplings for these states should be larger. Although the above estimates are lower bounds to the CT state contributions, we will see in the minimal model presented below that non-CT-SR effects, not related to excitonic couplings, determine the nonconservativity of the CD spectrum.

The CD spectrum is calculated using an expression derived earlier^46^ with slight modifications concerning an approximation of excitation energies that we find is not necessary, as discussed in the SI (Section 6.1.2). The expression for the CD spectrum contains two contributions:
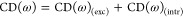
5The excitonic part reads
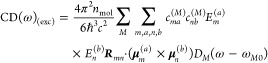
6where *n*_mol_ is the volume density of molecular complexes in the sample, ***R***_*mn*_ = ***R***_*m*_ – ***R***_*n*_ is the distance vector connecting the centers of pigments *m* and *n*, and *c* is the vacuum velocity of light. The intrinsic part of the CD signal is given as
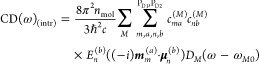
7where we only include the special pair, since the intrinsic contribution from monomeric pigments is known to be small. Note that the magnetic transition dipole moment ***m***_*m*_^(*a*)^ is a purely imaginary vector, such that (−*i*)***m***_*m*_^(*a*)^ is a real quantity. The electric transition dipole moments of the higher excited states (*a* ≠ Q_y_) are given as the diabatic transition dipole moments
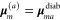
8whereas perturbation theory with respect to the coupling to CT states is included for the Q_y_ transition
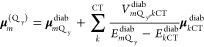
9

Please note that the CT states, obtained in our diabatization procedure, have small but nonzero transition dipole moments, as discussed above. By treating the CT states in the above perturbation theory, it is possible to circumvent the problem of defining a proper pigment center for the CT states, which would be needed, if the CT states were explicitly included in eq 6. The ***m***_*m*_^(*a*)^ for the higher excited states (*a* ≠ Q_y_) equals the intrinsic transition dipole moment that is obtained from the diabatic magnetic transition dipole moment ***m***_*ma*_^diab^ by subtracting the exciton contribution that is already included in CD(ω)_(exc)_ (eq 6)
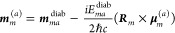
10(see SI, Section 6.1.2, eq (39)), with the diabatic electric transiton dipole moment **μ**_*m*_^(*a*)^ (eq 8). For the Q_y_ states, we again take into account the coupling to CT states in first-order perturbation theory
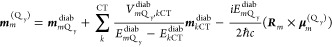
11where , obtained from eq 9, also contains the perturbation by CT states.

The line shape function *D*_*M*_(ω – ω_*M*0_) of the transition between the ground state and the *M*th exciton state peaks close to the energy *ℏω*_*M*0_ of this state, which is obtained as the *M*th eigenenergy of the exciton Hamiltonian in eq 1. From the eigenvectors of this Hamiltonian, the coefficients *c*_*ma*_^(*M*)^ result. Note that eq 6 is independent of the origin of the coordinate system despite the fact that the site energies *E*_*m*_^(*a*)^ and *E*_*n*_^(*b*)^ are not approximated by the photon energy *ℏω*. The latter approximation is necessary if the Rosenfeld equation^58^ is used (SI (Section 6.1.2), and Figure S7). Besides the CD spectrum, we also calculated the linear absorption and linear dichroism spectra. Expressions for the latter, including details of the line shape function, are given in the SI (Section 6.1.2).

In order to take into account systematic errors in the quantum chemical excitation energies and the effect of the protein environment, all local excitation energies *E*_*m*_^(*a*)^ of the special pair pigments are shifted by a constant (950 cm^–1^) such that the lowest excited state energies agree with the values obtained from previous fits of optical spectra.^36,47^ Note, that we further apply final site energy fits within the tolerance limit of the latter (±60 cm^–1^), the minor effects of which are shown in Figure S7.

Static disorder in local excitation energies and some electronic couplings (see below) was taken into account by randomly assigning values from Gaussian (normal) distribution functions, calculating the homogeneous spectra for the different realizations of static disorder, and finally averaging the homogeneous spectra. For the nonspecial pair pigments of the RC, we assume a width (fwhm) of 180 cm^–1^ for the distribution function, as done previously.^47^ For the special pair pigments, it can be expected that SR effects on site energies and couplings are particularly sensitive with respect to the conformational changes of the complex. Therefore, the width of the distribution of every element of the diabatic Hamiltonian of the special pair has been estimated by considering 110 geometry-optimized snapshots of the MD simulations, as discussed in more detail below and in the SI (Sections 4.1 and 5.2, Figure S6).

The optical spectra of PSII-RCs obtained by applying the outlined methodology are compared in Figure 2 to experimental data^24,44,59^ as well as to calculations neglecting any high-energy electronic states of the pigments and SR effects in the special pair. We will term the latter the ”Q_y_-only” model in the following, since only the Q_y_ transition of the Chl *a* and Pheo *a* pigments and their LR excitonic couplings are involved. Overall, a very good agreement between calculated and measured spectra is obtained for the full model, especially when considering the fact that the deviations between theory and experiment are comparable in magnitude to the deviations between the two different experimental sets of circular dichroism data. The agreement between the two sets of experimental data is much better in the linear absorption spectrum, indicating the high sensitivity of the CD spectrum to changes in the electronic structure. A notable deviation between theory and experiment is the theoretical underrepresentation of the shoulder occurring in both CD experiments (with different magnitude) around 673 nm. Among other uncertainties, it could be that this shoulder reflects a conformational substate not present in our calculations, which reveal unimodal distribution functions for energies and couplings (Figure S6). In our calculations of optical spectra, these distribution functions are approximated by single Gaussian functions. The differences between experimental CD spectra could be related to different sample preparations, giving rise to slightly different conformations that are sensed by the SR effects in the CD spectrum.

**Figure 2:**
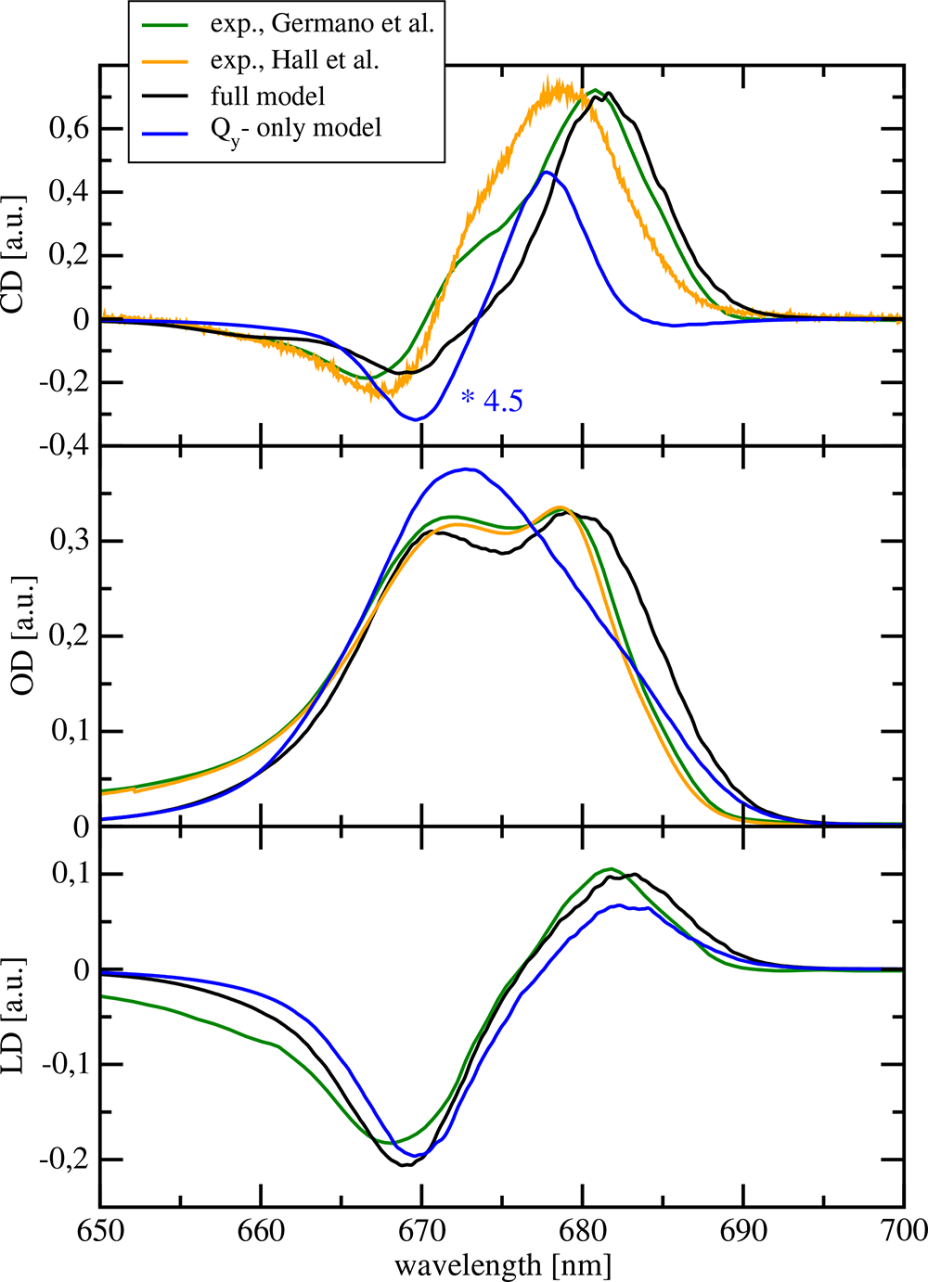
Comparison between low-temperature (*T* = 1.8 K) circular dichroism (upper panel), linear absorption (middle panel), and linear dichroism (lower panel) spectra, calculated with the full model (black) or the Q_y_-only model (blue) with the experimental data by Germano et al.^24,59^ (yellow) and Hall et al.^44^ (green).

The Q_y_-only model still provides a quantitatively correct description of the LD spectrum but misses the splitting between main absorption peaks in the OD spectrum and the strong nonconservativity of the CD spectrum. The deviations in the OD spectrum reflect the 45% smaller intradimer excitonic coupling obtained by excluding SR effects in the special pair. The deviations in the CD spectrum are due to the fact that in the Q_y_-only model, besides the missing SR effects, no high-energy excited states as well as no intrinsic magnetic transition dipole moments are included.

The CD spectrum obtained with the full model computations exhibits a ratio of nonconservativity of *R* = 3.4 ± 0.1 for the Q_y_-region, which is remarkably close to the experimental observation of *R* = 3.6. Neglecting any dynamic or static disorder effects that broaden the optical lines, a *R* value of only 1.5 is obtained. Obviously the overlap between optical lines with opposite signs has a strong effect on the apparent nonconservativity. Taking into account the homogeneous broadening results in only *R* = 1.7. Hence, the major effect comes from the inhomogeneous broadening.

Based on this finding, the role of static disorder in the special pair and the effects of CT states on the latter are investigated by considering different models, which do or do not take into account microscopic information from the additional 110 MD snapshots. Assuming an equal width of 180 cm^–1^ for all distribution functions of site energies *E*_*m*_^(*a*)^, already results in *R* = 3.1. In a next step, we included the non-CT-SR effects on the site energy variations by analyzing the distribution function for the energies of the diabatic Q_y_ states  obtained from the MD snapshots, resulting in fwhm of 160 cm^–1^ and 179 cm^–1^ for P_D1_ and P_D2_, respectively. Since these values are close to the 180 cm^–1^ assumed above, we still obtain *R* = 3.1. Taking into account the coupling to CT states (eq 2) results in an increased fwhm of 217 cm^–1^ and 211 cm^–1^ for  and  respectively, yielding an increased nonconservatitvity ratio of *R* = 3.3. If, in addition, the fluctuations of the excitonic coupling  (eq 3) is taken into account, *R* = 3.4 results. Please note that the major contribution (80%) to these fluctuations is due to the coupling to CT states. This effect can be explained by large fluctuations of CT state energies as well as CT-LE couplings due to the strong distance dependence of SR couplings (Figure S6). Similar results have been obtained for the case of the LH2 light-harvesting complex of purple bacteria.^60^

Based on the computed quantities of the full model, we derive a minimal model that comprises solely effective Q_y_-parameters but still allows for a sufficiently accurate calculation of optical spectra. In this way, the main contributions to the observed nonconservativity can be identified. Different simplifications of the full model have been investigated, as described in detail in the SI (Section 7). We finally arrive at an effective Q_y_ Hamiltonian of the RC reading
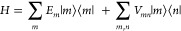
12which resembles the one of the Q_y_-only model, discussed above, except for the following adaptations concerning the special pair pigments: (i) an increased excitonic coupling  of 150 cm^–1^, that takes into account CT state coupling effects, and (ii) enhanced static disorder, described by an increased width (fwhm) of 280 cm^–1^ of the distribution function of the site energies *E*_P_D1__ and *E*_P_D2__, as compared to 180 cm^–1^ (fwhm) used for *m* ≠ P_D1_, *P*_*D*2_. In addition, our minimal model includes (iii) rotated electric and magnetic transition dipole moments of the special pair pigments, taking into account SR effects (eqs 9 and 11, respectively) and (iv) the resulting intrinsic contribution to the CD signal (eq 7). Please note, that site energies of the RC pigments proposed earlier^31,36^ are used.

Since only Q_y_ transitions are included, the excitonic part of the CD spectrum is conservative in the Q_y_ spectral region, that is ∫_Q_y__*dω*CD(ω)_exc_ = 0. This result follows from eq 6 by noting ∫_Q_y__*dωD*_*M*_(ω-ω_*M*0_) = 1 and ∑_*M*_*c*_*m*_^(*M*)^*c*_*n*_^(*M*)^ = δ_*m*,*n*_. The integral over the CD spectrum is then given as the integral over the intrinsic contribution, obtained from eq 7 in a similar way as
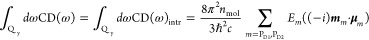
13Hence, the scalar product ((−*i*)***m***_*m*_ ·**μ**_*m*_) between the intrinsic magnetic and the electric transition dipole moments of the pigments is a decisive factor for the sign and magnitude of the integral intrinsic CD signal. Note that a negative (positive) sign corresponds to a nonconservative ratio < (>)1.

Neglecting SR effects on the transition dipole moments of the special pair, that is, using transition dipole moments obtained from quantum chemical calculations on the isolated monomers, results in a nonconservativity ratio of the CD spectrum of *R* = 0.9. The respective CD spectrum (Figure 3) has a sign-inverted integral compared to the spectrum obtained in the full model. Using the diabatic transition dipole moments including the couplings to CT states (eqs 9 and 11) instead of the isolated-monomer dipoles results in *R* = 3.3 and a CD spectrum that semiquantitatively resembles the spectrum obtained in the full model (Figure 3). Neglecting the coupling to CT states but including non-CT-SR effects (that is, using eqs 8 and 10 instead of eqs 9 and 11, respectively, for *a* = Q_y_) results in *R* = 2.8, reflecting the strong influence of non-CT-SR effects. Neglecting all SR effects on the magnetic transition dipole moments, that is, using the magnetic transition dipole moments of the isolated P_D1_ and P_D2_ monomers, still results in a *R* = 2.5. Hence, we find the impact of non-CT-SR effects on the electric transition dipole moments to be the main source of nonconservativity. We note in passing that despite the stronger influence of non-CT-SR effects on the electric than on the magnetic transition dipole moment of the Q_y_ transition of the P_D1_ and P_D2_ pigments, the magnetic transition dipole moments of the higher excited states are affected stronger than the electric ones (SI, Section 8, Figures S15 and S16). These higher excited state transition dipole moments, however, have practically no effect on the spectra in the Q_y_ spectral region, as our minimal model demonstrates.

**Figure 3:**
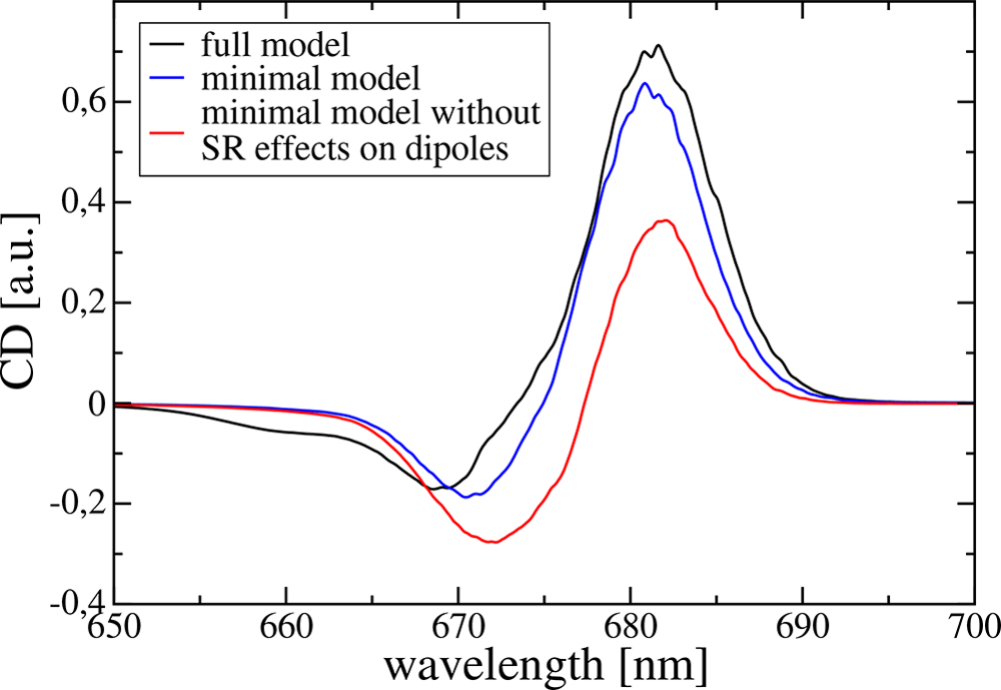
CD spectrum calculated in the full model (black line; same as in Figure 2) is compared to calculations using the minimal model, where Q_y_-transition dipoles  and  (*m* = P_D1_, *P*_*D*2_) from calculations on isolated monomers (red line) or from eqs 9 and 11, respectively, (blue line) are used.

In Table 1, we provide the angles of the electric and intrinsic magnetic transition dipole moments used for the minimal model, where the influence of non-CT-SR coupling as well as CT state coupling is considered compared to the Q_y_-only model. The angles Θ and Φ are defined, as shown in Figure 4.

**Figure 4:**
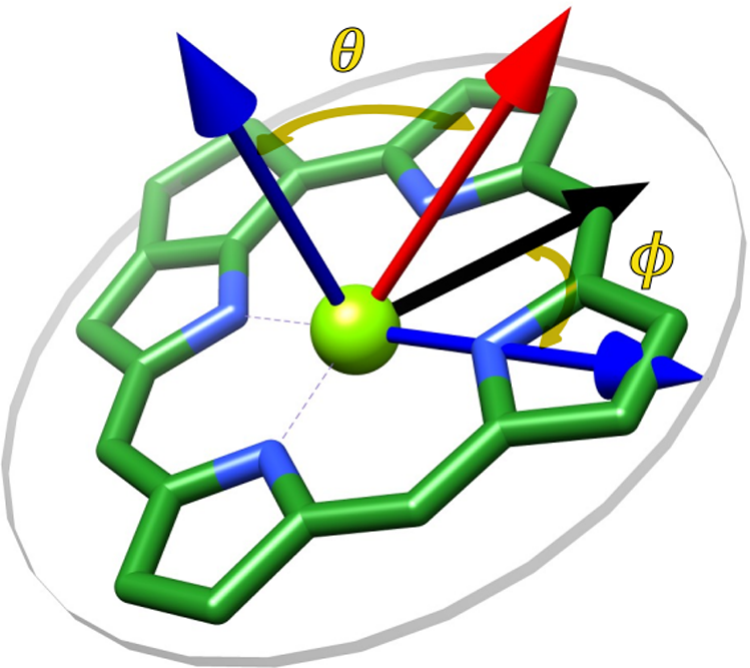
Definition of angles Θ and Φ with respect to reference vectors analogously to spherical coordinates; Θ is the angle between vector (red) and pigment plane normal vector (blue), Φ is the angle between N_B_-N_D_ axis (blue) and vector projected on pigment plane (black)

**Table 1:** Q_y_ Electric and Magnetic Transition Dipole Moments of the Special Pair Pigments

*m*	P_D1_		P_D2_	
μ_*m*_^Qy^	Θ[°]^d^	Φ[°]^d^	Θ[°]^d^	Φ[°]^d^
mon.^a^	90.2	1.0	89.9	0.6
dimer^b^	85.6	3.1	93.9	6.4
*m*_*m*_^Qy^	Θ[°]	Φ[°]	Θ[°]	Φ[°]
mon.^a^	2.2	71.0	1.3	71.2
dimer^c^	4.0	89.9	4.0	87.8
	mon.	dimer	mon.	dimer
	–0.015	0.081	–0.010	0.077

a isolated monomers,

b eq 9,

c eq 11

d defined in Figure 4

By comparing the orientations of transition dipole moments obtained from quantum chemical calculations on the isolated monomers with those obtained from dimer calculations, subsequent diabatization, and perturbation by CT states, the SR effects on the orientation of the transition dipoles can be revealed. For the isolated monomers, we obtain electric transition dipole moments **μ**_*m*_ lying in the pigment planes (Θ = 90°) and intrinsic magnetic transition dipole moments ***m***_*m*_ that are oriented almost parallel to the normal on the pigment planes (Θ = 1°–2°).

From the monomer calculations, scalar products between (normalized) intrinsic magnetic and electric transition dipole moments of −0.015 and −0.010 are obtained for P_D1_ and P_D2_, respectively (Table 1). The negative signs reflect a nonconservativity ratio < 1 of the CD spectrum, obtained from the minimal model by neglecting SR effects on the transition dipole moments (Figure 3). This result is also consistent with the fact that a slightly negative intrinsic CD spectrum has been measured on isolated Chl *a*.^61^

Interestingly, the analysis of transition dipole moments of the special pair pigments shows that SR effects cause small but distinct rotations (Table 1). In particular, the electric transition dipole moments are tilted out of the pigment planes toward the other pigment in the special pair by about 4°, as illustrated in Figure 5. As shown there, non-CT-SR effects dominate the tilt of electric transition dipole moments. This rotation is sufficient to invert the sign of the scalar product ((−*i*)***m***_*m*_·**μ**_*m*_) and to increase its magnitude 6-fold to 0.081 and 0.077 for P_D1_ and P_D2_, respectively. The sign change explains why the intrinsic contribution to the CD now leads to a nonconservativity ratio > 1. The larger magnitude of the scalar product appears in the spectrum as a larger nonconservativity ratio of 3.3 as compared to 0.9 obtained by using monomer transition dipole moments in the minimal model (Figure 3). Note, that differences regarding the magnitude of electric dipole moments, also contained in our diabatization procedure, on the obtained spectra are minor (Figure S8).

**Figure 5:**
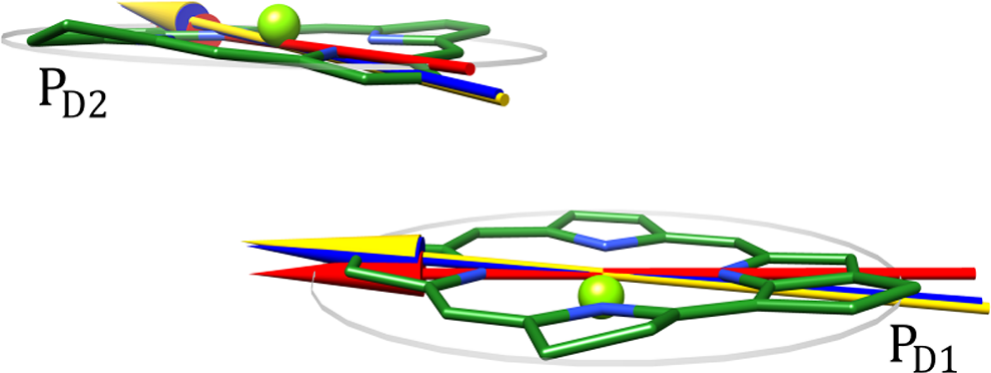
Illustration of the rotation of electric transition dipole moments  and  by non-CT-SR effects. Red: isolated monomer, blue:  in eq 9, yellow: full eq 9.

We have checked that the observed rotation of Q_y_ transition dipole moments is real and not caused by a computational artifact, such as a basis set superposition error, or by electrostatic polarization effects, as described in detail in the SI (Section 10). Increasing the distance between the special pair pigments by moving P_D2_ along the normal on the pigment plane of P_D1_, the wave function overlap is decreased and the orientations of transition dipole moments approach that of the isolated pigments (Figure S18). The largest changes occur for distance increases between Δ*r* = 0 Å (native structure) and Δ*r* = 3 Å, reflecting the SR effects. The minor changes observed for Δ*r* up to 10 Å also show that small LR effects are present, which could be due to dispersive interactions in the special pair leading to a mutual polarization of LE states.

Treating the special pair as a supermolecule, characterized by quantum chemical calculations in the framework of an exciton model of the reaction center, results in a nonconservativity ratio of 3.6, in excellent agreement with the experiment and with the results obtained with the diabatization technique in the full model above (SI Section 9).

We finally want to come back to our previous model,^47^ where, without including SR effects on the transition dipole moments of the special pair, but including the coupling between Q_y_ states and higher LE states of the RC pigments, a nonconservativity ratio of *R* = 2.3 was obtained,^47^ that could be increased to *R* = 2.9 by assuming a 3-fold enhanced excitonic coupling between Q_y_ and high-energy LE states in the special pair. It was proposed that the enhancement could occur due to superexchange via the CT states. As noted above, this effect could not be found in the present calculations. In order to clarify the remaining discrepancy between our previous and the present work, we applied the same methodology as before^47^ and obtained *R* = 1.4. The remaining discrepancy to the earlier value of *R* = 2.3 is caused by the choice and calculation of special pair parameters (LE state energies, couplings, and transition dipole moments) as explained in the SI (Section 6.2). Obviously, the coupling to high-energy transitions in the case of PSII RCs is not the main contributor to the nonconservativity of the CD spectrum, in contrast to other pigment–protein complexes.^45,46^

An open point of the present work concerns the temporal characterization of conformational dynamics by QM/MM simulations. So far, we have attributed the enhancement of site energy fluctuations by CT state couplings to the static disorder. However, it will be more realistic to include the fast fluctuations in the spectral density of the exciton-vibrational coupling, leading to stronger homogeneous broadening that could lead to different overlap effects between optical lines in the CD spectrum influencing the apparent nonconservativity ratio. A related point concerns the modeling of solubilized complexes (including their detergent belts) that could reveal information about possible conformational substates probed by the optical experiments on such samples. We note that the detergent has an influence on SR-effects in the special pair of bRC due to a shift of conformational equilibria.^62^

In summary, we have quantified the importance of SR effects in the special pair of PSII RCs. Two main findings are (i) the coupling between LE and CT states significantly enhances the excitonic coupling as well as static disorder in local transition energies within the special pair, and (ii) intra special pair non-CT-SR effects change the direction of electric Q_y_ transition dipole moments, causing the strong nonconservativity of the CD spectrum in the low-energy region. It is interesting to note that CT state coupling and non-CT-SR effects have distinct consequences. The first enhances the excitonic coupling, and the second rotates the electric transition dipole moments of the special pair pigments toward the respective other pigment.

## Supporting Information Available

Overview and methodology, protonation pattern calculations, molecular dynamics simulations, structure preparation, parameterization of special pair Hamiltonian, computation of CD-spectra, construction of a minimal model, dipole moment analysis, embedding special pair adiabatic states into the reaction center Hamiltonian, assessment of possible errors (PDF)
Atomic coordinates of the representative structure of the special pair (XYZ)
Hamiltonian parameters of the full model (XLSX)
Hamiltonian parameters of the special pair after diabatization (XLSX)
Hamiltonian parameters of the special pair after diabatization and perturbation by CT states (XLSX)
Transparent Peer Review report available (PDF)

## Supplementary Material

jz3c02693_si_001.pdf

jz3c02693_si_002.xyz
