# Supplementary material for: Short-Range Effects in the Special Pair of Photosystem II Reaction Centers: The Nonconservative Nature of Circular Dichroism
Source: J Phys Chem Lett. 2023 Dec 20;14(51):11758–67. doi: 10.1021/acs.jpclett.3c02693 (PMC10758115; doi:10.1021/acs.jpclett.3c02693)
Supplement: Supplementary file 1 — jz3c02693_si_001.pdf [file jz3c02693_si_001.pdf]

Supplementary Material: Short-Range Effects in the Special Pair of Photosystem II Reaction Centers: The Non-Conservative Nature of Circular Dichroism

Felix G. Gemeinhardt[1,5], Yigal Lahav[2,3,5], Igor Shapiro[2], Dror Noy[3,4], Frank Müh[1], Dominik Lindorfer[1], and Thomas Renger[1]

[1]Institut für Theoretische Physik, Johannes Kepler Universität Linz, Altenberger Strasse 69, 4040 Linz, Austria

[2]Fritz Haber Center for Molecular Dynamics Research, Institute of Chemistry, Hebrew University of Jerusalem, 9190401 Jerusalem, Israel

[3]MIGAL - Galilee Research Institute, S. Industrial Zone, 1101602 Kiryat Shmona, Israel

[4]Faculty of Sciences and Technology, Tel-Hai Academic College, Upper Galilee, Israel

[5]Contributed equally to the present work.

Contents

1 Overview of methodology **S2**

2 Protonation pattern calculations **S4**
- 2.1 Method . . . S4
- 2.2 Results . . . S5

3 Molecular dynamics simulations **S5**
- 3.1 Method . . . S5
- 3.2 Results . . . S6

4 Structure preparation **S7**
- 4.1 Method . . . S7
- 4.2 Results . . . S8

5 Parameterization of special pair Hamiltonian **S9**
- 5.1 Method . . . S9
- 5.2 Results . . . S13

6 Computation of CD-spectra **S17**
- 6.1 Method . . . S17
  - 6.1.1 Embedding the Special Pair Hamiltonian into the Reaction Center Hamiltonian . . . S17
  - 6.1.2 Theory of Circular Dichroism Spectra . . . S19
- 6.2 CD spectra for alternative parameters and theories . . . S24

7 **Construction of a minimal model** **S27**

8 **Dipole moment analysis** **S32**

9 **Embedding special pair adiabatic states into the reaction center Hamiltonian** **S35**

10 **Assessment of possible errors** **S35**

1 Overview of methodology

The overall computation protocol is illustrated in Figure S1. Each step is discussed in detail in the upcoming sections. Here we present a short overview.

The coordinates of the D1D2cytb_{559} complex were extracted from that of the PSII core complex (pdb file 3WU2.pdb).[2] After adding hydrogen atoms, the protonation probabilities of titratable residues were calculated and molecular dynamics (MD) simulations were performed. 110 snapshots of the MD simulations were geometry optimized by QM/MM, including the special pair in the QM part. Based on an analysis of the snapshots, we define the snapshot with the lowest root mean square deviation (RMSD) to the average special pair coordinates as the representative special pair structure. Excited states of the special pair were calculated treating the remaining pigments and the protein environment by atomic partial charges (electrostatic embedding), for the representative structure and the remaining snapshots. These calculations are analyzed with the diabatization revealing local excited and charge transfer states as well as couplings. From the distribution functions of these quantities, obtained from the snapshots, static disorder is estimated. For the representative structure, in addition, quantum chemical calculations of excited states of the isolated monomers are performed, which are used to obtain the long-range excitonic coupling in the special pair. The energies and couplings obtained for the representative structure are post-processed, based on independent experimental data. The effects of charge transfer states are accounted for by perturbation theory, i.e., Q_y energies and dipoles, as well as couplings comprising at least one Q_y state are perturbed. The excitonic couplings between the special pair and the remaining RC pigments are obtained with the Poisson-TrEsp method for the representative structure. The parameters for the remaining RC pigments are taken from our earlier work.[1] Using these parameters of the Frenkel exciton Hamiltonian, the circular dichroism spectra are calculated in the full model, including higher excited states of the pigments, and a minimal model that takes into account the Q_y transitions only, but includes an enhanced excitonic coupling in the special pair due to coupling to CT states and a rotation of electríc and magnetic transition dipole moments by non-CT short-range effects.

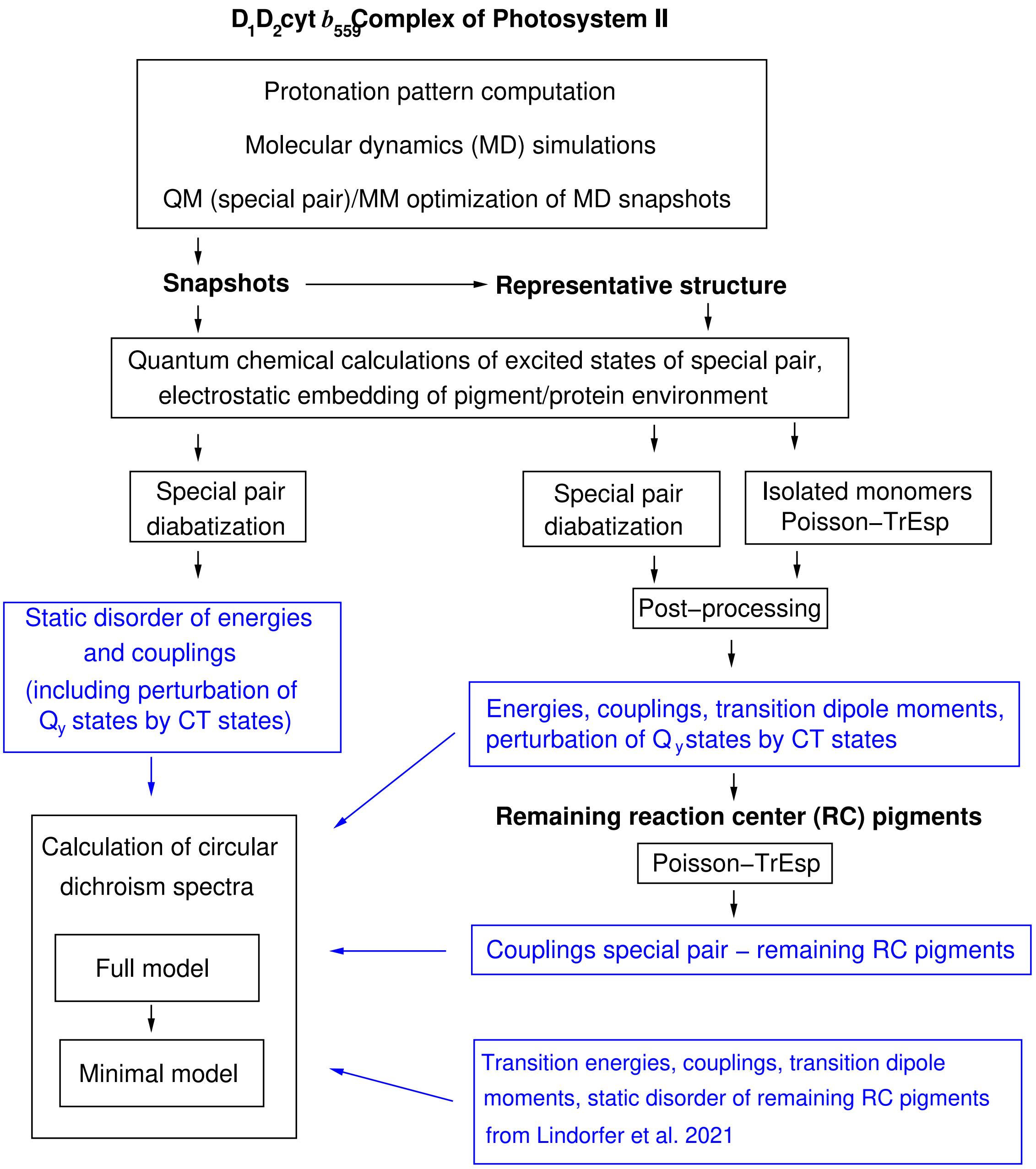

Figure S1: Overall computation protocol. Bold: Structural data, black boxes: applied methods, blue boxes: parameters of Frenkel exciton Hamiltonian, for Lindorfer et al. 2021, see ref.[1]

2 Protonation pattern calculations

2.1 Method

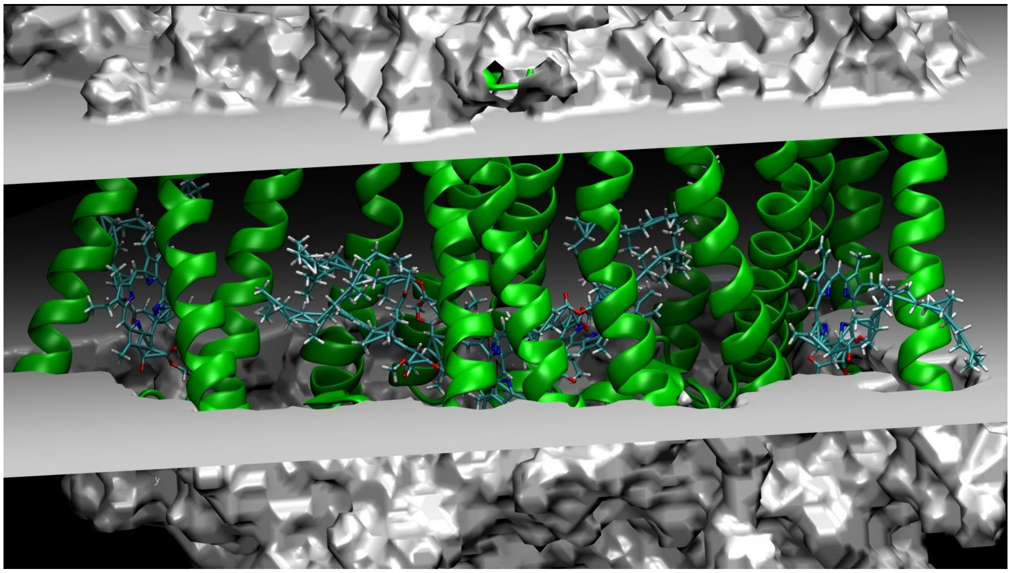

Figure S2: Model of PSII-RC (isosurface level of boundary between dielectric constants of the solvent ($\epsilon_{\mathrm{solv}} = 80$), the protein ($\epsilon_{\mathrm{p}} = 4$) and the membrane interior ($\epsilon_{\mathrm{mem}} = 2$).

The method for determining the protonation probabilities of titratable residues has been reviewed[3] and applied to the reaction center of PSII.[4] The overall process is given by i) preparing the structure for the PSII reaction center, ii) solving the Linearized Poisson-Boltzmann Equation (LPBE), and iii) running a Monte-Carlo titration.

The used structure is based on the crystal structure for the cyanobacterial PSII of *T. vulcanus* (PDB 3WU2) provided by Umena et al. (2011),[2] which shows a resolution of 1.9 Å. The structural elements contained in the full reaction center model are based on ref,[4] but omitting the J and X chain as well as the atoms of the water-oxidizing complex Mn_4CaO_5 (WOC), since these structural elements are expected to be absent in the D1D2cytb_{559} PSII-RC preparations on which the optical spectra are measured. Special attention has been paid to the protonation states of the ligands of the missing WOC, which were supposed to compensate for its charges. All ligands belonging to the reaction center have been taken into account. Neglecting the CP43-Glu-354 ligand can be justified by its absence in the PSII-RC preparations.

In the second step, the LPBE is solved using TAPBS.[5,6] After the definition of the protein and membrane, as well as the assignment of dielectric constants ($\epsilon_{mem} = 2$, $\epsilon_p = 4$, $\epsilon_{solv} = 80$), the LPBE is solved on a grid using a finite difference method and a grid focusing approach. The model for the PSII reaction center is depicted in Figure S2. Standard parameters have been used for the calculation (e.g., temperature: 300 K, ion radius: 2.00 Å, etc.).

Finally, the calculation of the average protonation state for each titratable group has been conducted with the Karlsberg2 software,[7] where a range of pH-values between 4 and 11 has been considered.

2.2 Results

In the following, the most probable protonation states for the histidines, the titratable groups of the ligands, as well as other titratable groups are presented. Regarding its protonation state, histidine represents an exception to the other considered amino acids. At physiological pH-values, histidine may occur in its protonated (HSP), or two different deprotonated forms (HSD, HSE). The obtained results are given in Table S1. The protonation states of the WOC ligands are presented in Table S2. It can be readily seen, that the missing charges of the Mn_4CaO_5 complex are compensated by the obtained protonation states of its ligands, as expected. Finally, only one other titratable group (Glu-D312) has been found to deviate from its standard

Table S1: Protonation states of histidines

	His-A92	His-A190	His-A195	His-A252	His-A304	His-A332	His-A337	His-D61	His-D87	His-D189	His-D336
**Result**	HSP	HSP	HSE	HSD	HSP	HSP	HSP	HSP	HSE	HSE	HSE

Table S2: Protonation states of WOC ligands

	Asp-A61	Asp-A170	Asp-A342	Ala-A344 (CTER)	Glu-A189	Glu-A333	His-A337	His-A332
**Standard**	D	D	D	D	D	D	-	-
**Result**	P	P	D	P	P	D	HSP	HSP

protonation state (deprotonated) at a pH-value of 6.5. However, since Glu-D219 shows a probability of only 50% to be protonated, it is still modelled in its standard form. The results can be compared to current findings in the literature. In this regard, Narzi and Guidoni[8] conducted MD simulations of the Apo-PSII with different protonation states for the WOC ligands. The considered protonation pattern which is closest to the results presented above has been taken from Han et al. (2019).[9] This pattern has been found to be the most stable one in terms of RMSD during the MD simulation of Narzi and Guidoni.

3 Molecular dynamics simulations

3.1 Method

Molecular dynamics preparation After the most probable protonation states were determined, the system was embedded in a DOPC membrane with KCl salt concentration of 0.15 mol using CHARMM-GUI.[10] The protein and cofactors were parameterized using the Amber ff14SB Force-Field (FF),[11] while the lipid17 FF used to describe the membrane and the TIP3P[12] model for water molecules. Missing parameters for metal-ligand complexes were calculated using the Seminario method[13] as implemented in the MCPB protocol.[14] Several types of metal-ligand complexes are found in our PSII model. These include: six chlorophylls (Chls), the heme of Cytb_{559}, and the non-heme iron at the interface of proteins D1 and D2. Two sets of parameters were calculated for the Chls. The first describes a Mg^{2+} ion coordinated to five nitrogens, the four nitrogens of the chlorin ring and the ϵ nitrogen of a histidine residue. The second set of parameters only describes the coordination between the Mg atoms and the four chlorin nitrogens. The former was used to describe the Chls P_{D1}, P_{D2}, $Chlz_{D1}$ and $Chlz_{D2}$, whereas the latter was used to describe the Chls Chl_{D1} and Chl_{D2}. The cytb_{559} heme was parameterized as an octahedral complex of low-spin Fe^{+2} iron coordinated to six nitrogens: two ϵ nitrogens of histidine, and the four nitrogens of the porphyrin. The heme propionate functional groups were taken to be deprotonated. The non-heme iron was also parameterized as an octahedral complex, however this time the ligands are four ϵ nitrogens of histidine and two oxygen atoms of bicarbonate. The bicarbonate was protonated at the oxygen facing away from the iron, and the complex was modeled as a

high-spin Fe^{+2} complex. The last two residues which require special attention are alanine with protonated C-terminus,and a methionine with formylated N-terminus. For these residues, we used the prepgen tool from the Amber toolbox.[15] All missing parameters and RESP charges were calculated in the gas-phase at the B3LYP[16]/6-31G*[17] level of theory using Gaussian16.[18]

Molecular dynamics simulation Starting with our reduced PSII model, initial geometry optimization was carried out with spatial restraints applied to all heavy atoms, excluding water oxygens. For backbone atoms, a restraint weight of 10.0 kcal/mol was used, while for all other atoms (side chain, ligands, lipids and ions), a restraint of 5.0 kcal/mol was applied. Following the optimization step, a heating simulation from 100 K to 300 K was carried out using a NVT ensemble for 800 ps while maintaining the spatial restraints. After the heating simulation was complete, the final geometry and velocities were used as a starting point for a ten step equilibration protocol, in which the spatial restraints were gradually removed (see table S3). Each equilibration step was carried out for 500 ps using a NPT ensemble with semi-isotropic pressure scaling. After all restraints were removed, the simulation was continued for additional 5 ns. During these parts of the simulation, a time step of 1 fs was used. The last geometry of the equilibration simulation was used as the starting point of a 240 ns production run for the NPT ensemble. Here, new velocities were assigned based on a Boltzmann distribution, and the time step was increased to 2 fs. During all steps of the molecular dynamics, SHAKE constraints were applied to all hydrogen involving bonds, and a Langevin thermostat with collision rate of 5 ps^{-1} was used.

step	backbone	side-chains	lipid heads	lipid tails	ligands	ions
1	10.0	5.0	2.5	5.0	5.0	5.0
2	10.0	5.0	2.5	2.5	5.0	5.0
3	10.0	5.0	1.0	2.5	5.0	5.0
4	10.0	5.0	0.0	1.0	5.0	5.0
5	10.0	2.5	0.0	0.0	2.5	2.5
6	10.0	2.5	0.0	0.0	1.0	1.0
7	10.0	1.0	0.0	0.0	0.0	0.0
8	5.0	0.0	0.0	0.0	0.0	0.0
9	2.5	0.0	0.0	0.0	0.0	0.0
10	1.0	0.0	0.0	0.0	0.0	0.0

Table S3: Restraint weights during the equilibration steps in kcal/mol .

3.2 Results

To measure the quality of the MD simulation, we investigated the backbone RMSD with respect to the crystal structure. In this way, we can see, if our reduced model is stable, and examine how far it drifts away from the original structure. Figure S3 shows the backbone RMSD of the initial production run. Overall, the RMSD shows stability over the course of the MD simulations and the backbone does not deviate much from the crystal structure. However, within the first 40 ns of the simulation, the fluctuations in the RMSD are still relatively rapid. Therefore, these first 40 ns were taken as equilibration phase.

Figure S4 shows the backbone RMSDs of the second stage production run. Since all MD simulations reveal a stable structure, these simulations are used for sampling geometries representing the static disorder in the system.

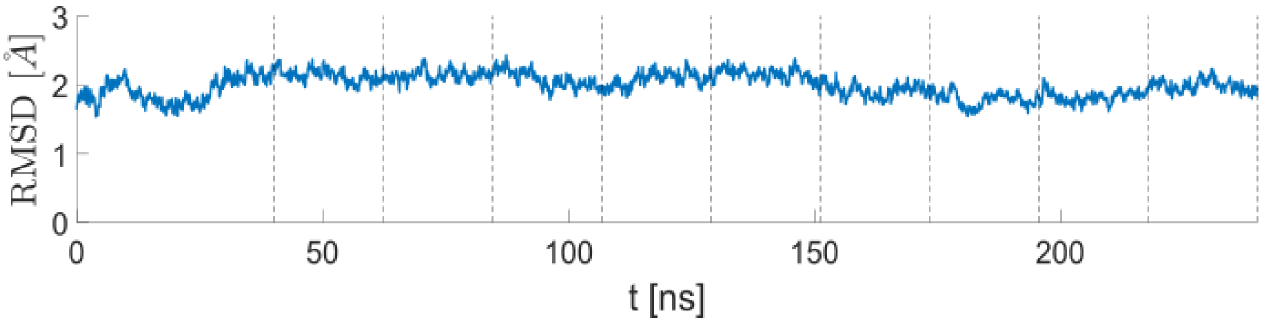

Figure S3: Root mean square deviation (RMSD) of the protein backbone, with respect to crystal structure, during the MD simulation. For further exploration of configuration space, ten snapshots with equal time spaces (marked by dashed lines) were taken from the last 200 ns of the trajectory.

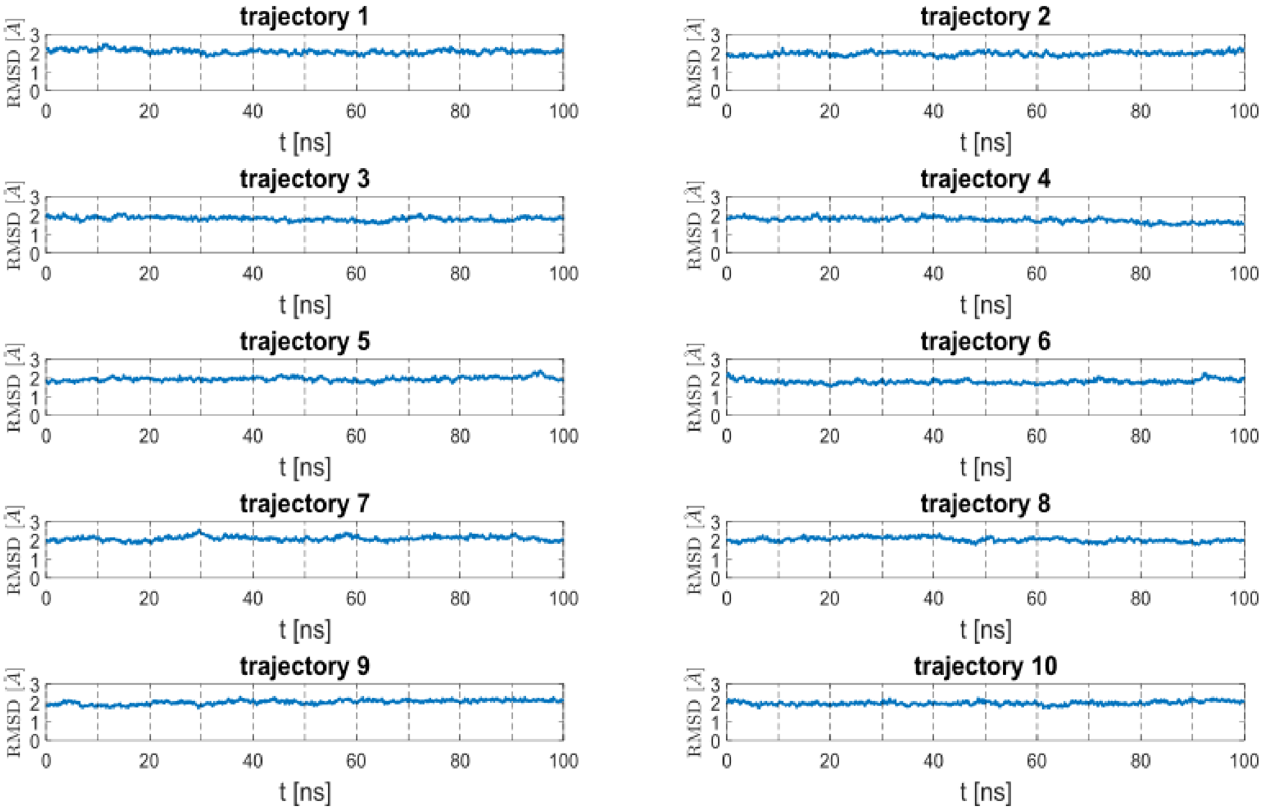

Figure S4: Root mean square deviation (RMSD) of the protein backbone, with respect to the crystal structure, of ten additional MD simulations. Eleven snapshots from each trajectory (marked by dashed lines) were taken for calculating the static disorder.

4 Structure preparation

4.1 Method

Generating a representative structure and static disorder In order to choose a representative structure of our PSII model, we selected the snapshot showing the smallest RMSD deviation from the average backbone geometry. For generating static disorder, we further explored the phase space in the MD simulations. Ten snapshots, with equal time spaces between them, were taken out of the last 200 ns of the production run.

New velocities were assigned and additional 100 ns production simulations were performed. From each trajectory, eleven snapshots with equal time spaces were sampled. All the sampled snapshots (including the representative structure) were optimized at the MM level, using the conjugate-gradient algorithm in AMBER.

QM/MM Optimization All MM optimized structures went through a further QM/MM optimization protocol using the ORCA 5.0.3 software.[19] QM/MM partitioning was done by truncating the $C_\alpha - C_\beta$ bonds of the Chl-ligating histidines and the $P_1 - P_2$ bonds of the phytyl chains (atoms denoted as $C_1 - C_2$ in the pdb file). The truncated bonds were saturated with hydrogen link atoms (Fig. S5). The QM-MM interaction was calculated by electrostatic embedding. Since ORCA does not support periodic boundary conditions, all MM atoms were fixed to their positions and only the QM part was optimized, and non-bonded interactions were calculated explicitly (no cutoff). In order to reduce computation time, we initially optimized the structures at the XTB semi-empirical DFT level of theory,[20] using the ORCA L-BFGS optimizer. Next, a second QM/MM optimization step was carried out at the B3LYP/def2-SVP[21] level of theory with Grimme's D3 dispersion correction with Becke-Johnson damping.[22] In this stage, the RIJCOSX approximation[23] was used for calculating the Coulomb and HF-exchange integrals.

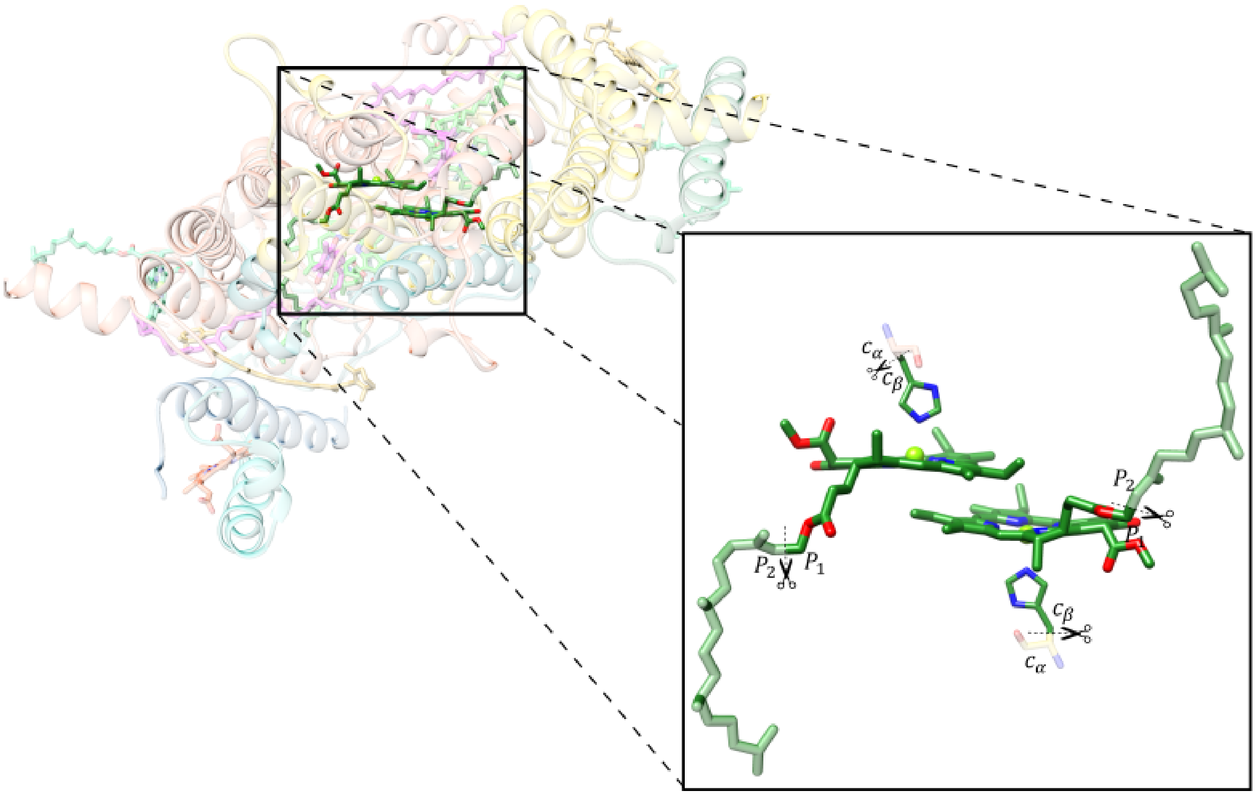

Figure S5: QM/MM partitioning. The QM region is made of the special pair Chls and their their fifth-ligand histidine side chains. Phytyl tails were omitted for the QM region by truncating the $P_1 - P_2$ bond and saturating it with a hydrogen link atom.

4.2 Results

The representative structure of the special pair is provided as an additional xyz-file "SP_Repr_Structure.xyz".

5 Parameterization of special pair Hamiltonian

5.1 Method

Excited states calculation The first 30 adiabatic excited states of the special pair structures mentioned above have been calculated by linear response TD-DFT at the ωB97X-D3[24]/6-31G* level using the AMBER-Terachem interface[25] with a direct cutoff of 50 Å for the QM atoms. In this way, we can take advantage of Terachem[26,27] GPU acceleration and calculate excited states for a large number of snapshots within a feasible time frame. In addition, the representative structure was subject to excited state calculation at the ωB97X-D3(BJ)[28]/def2-SVP level of theory using ORCA.[19] In order to save computation time, the RIJCOSX approximation was applied. The preference for the def2-SVP over the similar 6-31G* basis set is rooted in the need for an auxiliary basis set when using the RIJCOSX approximation [i], which is explicitly given for the def2-SVP basis set (def2/J).[29] Furthermore, initial comparative calculations revealed only very minor differences regarding relevant results and very similar computation times between the 6-31G* and def2-SVP basis sets.

Diabatization In the following, the Multi-FED-FCD[30] method is outlined, which has been applied for diabatization. As its name suggests, this method builds on two previously proposed diabatization procedures, i.e., the Fragment Charge Difference (FCD),[31] and the Fragment Excitation Difference (FED)[32] method. These methods take two adiabatic states, the diagonal Hamiltonian in the adiabatic representation, and a certain operator in the adiabatic basis as inputs and yield two diabatic states and the Hamiltonian in the diabatic representation. The underlying rationale is to find an operator which i) is diagonal in the diabatic representation with diagonal elements being at their maximum/minimum, and ii) can be used to sort diabatic states according to their eigenvalues with respect to this operator. Taking the FCD method with the charge difference operator $\Delta q_{mn} = \int_{\mathbf{r}\epsilon A} \rho_{mn}(\mathbf{r}) d\mathbf{r} - \int_{\mathbf{r}\epsilon B} \rho_{mn}(\mathbf{r}) d\mathbf{r}$ (fragments: A, B; transition densities of states m, n: ρ_{mn}) as an illustrative example, the procedure of the fragment difference methods follows the subsequent steps:

1. Calculation of $\Delta\mathbf{q}$ in the adiabatic basis and diagonalization to obtain the adiabatic-to-diabatic transformation $\mathbf{U}$:
$$\Delta\mathbf{q}^{'} = \begin{pmatrix} \Delta q^{'}_{aa} & 0 \\ 0 & \Delta q^{'}_{bb} \end{pmatrix} = \mathbf{U}^{\dagger} \Delta\mathbf{q} \mathbf{U}$$
with $\Delta\mathbf{q}^{'}$ being the charge difference operator in the basis of diabatic states a and b.

2. Transformation of the adiabatic Hamiltonian into the diabatic representation:
$$\mathbf{U}^{\dagger} \begin{pmatrix} E_{11} & 0 \\ 0 & E_{22} \end{pmatrix} \mathbf{U} = \begin{pmatrix} H_{aa} & H_{ab} \\ H_{ba} & H_{bb} \end{pmatrix}$$
.

3. Calculation of diabatic states: $(\Psi_a, \Psi_b) = (\Psi_1, \Psi_2)\mathbf{U}$.

Following the same procedure but utilizing the excitation difference operator $\Delta x_{mn} = \int_{\mathbf{r}\epsilon A} \rho^{ex}_{mn}(\mathbf{r}) d\mathbf{r} - \int_{\mathbf{r}\epsilon B} \rho^{ex}_{mn}(\mathbf{r}) d\mathbf{r}$, the FED scheme allows to identify different LE states instead of CT states. Here, the excitation

[i] `https://sites.google.com/site/orcainputlibrary/basis-sets/ri-and-auxiliary-basis-sets`

transition density $\rho_{mn}^{ex} = \rho_{mn}^{elec} + \rho_{mn}^{hole}$ is defined in the CIS-picture as

$$\rho_{mn}^{elec}(\mathbf{r}) = \sum_{i}^{N_{occ}} \sum_{a,b}^{N_{vir}} c_{ia}^{(m)} c_{ib}^{(n)*} \Phi_a(\mathbf{r}) \Phi_b^*(\mathbf{r}) \tag{1}$$

$$\rho_{mn}^{hole}(\mathbf{r}) = \sum_{i,j}^{N_{occ}} \sum_{a}^{N_{vir}} c_{ia}^{(m)} c_{ja}^{(n)*} \Phi_i(\mathbf{r}) \Phi_j^*(\mathbf{r}) \tag{2}$$

with Φ denoting a molecular orbital, and $c_{ia}^{(m)}$ being the CIS coefficient which describes the excitation from an occupied molecular orbital Φ_i to a virtual molecular orbital Φ_a of state m. At this point, the similarity between the transition density and excitation transition density, $\rho_{mn}^{ex} = \rho_{mn}^{elec} + \rho_{mn}^{hole}$ and $\rho_{mn} = \rho_{mn}^{elec} - \rho_{mn}^{hole}$, should be highlighted, suggesting that the combination of FCD and FED is efficient to compute. A nice illustration of the above idea of utilizing electron and hole densities to identify LE- and CT states can be found in Fig. 1 of the recent work by Wang *et al.*[33]

The above methods show some shortcomings like, e.g., the open question of how to best select the two adiabatic states (or the one state per fragment), which should span a similar space like the diabatic ones. Furthermore, a multi state treatment is not possible. Motivated by these shortcomings, the Multi-FED-FCD[30] has been developed. The Multi-FED-FCD method relies on previous works which address more than two states in the calculation and automatically assign states based on their eigenvalues, namely the 3-/4 state FCD approach[34] and the Multi state FCD approach.[35] Building on this knowledge, the Multi-FED-FCD scheme i) takes many adiabatic states as input to avoid manual adiabatic state selection, and ii) automatically assigns LE- and CT states according to their eigenvalues by an efficient combination of the FCD and FED operators. In more detail, the Multi-FED-FCD process follows the subsequent steps:

1. Definition and calculation of a new operator in the adiabatic basis, which serves to distinguish between LE- and CT states: $\mathbf{D} = (\Delta\mathbf{q})^2 - (\Delta\mathbf{x})^2$ with eigenvalues +1 (CT state) and -1 (LE state),

2. Diagonalization of $\mathbf{D}$ and sorting of eigenvectors according to eigenvalues to separate the subspaces: $\mathbf{D'} = \mathbf{U}_1^\dagger \mathbf{D} \mathbf{U}_1$,

3. Transformation of $\Delta\mathbf{q}$ and $\Delta\mathbf{x}$ into this new basis and diagonalization within subspaces to obtain the block-diagonal transformation matrix $\mathbf{U}_2 : \Delta\mathbf{q}'' = \mathbf{U}_2^\dagger \mathbf{U}_1^\dagger \Delta\mathbf{q} \mathbf{U}_1 \mathbf{U}_2$, $\Delta\mathbf{x}'' = \mathbf{U}_2^\dagger \mathbf{U}_1^\dagger \Delta\mathbf{x} \mathbf{U}_1 \mathbf{U}_2$,

4. At this stage, the transformation represented by $\mathbf{U}_1\mathbf{U}_2$ already transforms into a diabatic basis. However, after a second separation into LE1-, LE2-, CT1-, and CT2-subspaces according to the eigenvalues of the charge and excitation difference operators, a third transformation $\mathbf{U}_3$ is applied which introduces the assumption that diabatic states of the same type should have zero electronic coupling. This transformation is indicated in Figure S1 of the supplementary information of ref[30] by the resulting ϵ-blocks which represent diagonal submatrices. Therefore, the couplings between LE states on the same pigment, and between CT states where the hole and particle are localized on the same fragments, are zero. This assumption, although frequently used (e.g.,[33–35]), has been questioned in the literature[36] for the case of multimers though. After transforming the Hamiltonian to the intermediary diabatic representation with $\mathbf{U}_1$ and $\mathbf{U}_2$, the block-diagonal transformation matrix $\mathbf{U}_3$ is constructed by rediagonalizing the Hamiltonian in the respective four diabatic subspaces. The resulting overall scheme is visualized nicely in Figure S1 of the supplementary information of ref,[30] noted already above, and the resulting adiabatic-to-diabatic transformation matrix is represented by $\mathbf{U}_1\mathbf{U}_2\mathbf{U}_3$.

Although the Multi-FED-FCD method removes the necessity of selecting adiabatic input states manually, there remains the question of how many adiabatic states are required for a sufficient description of the

obtained diabatic states and elements of the Hamiltonian in the diabatic representation. A possible answer to this quality-related question is already provided in[35] with a convergence criterion of the quantity of interest (e.g., a certain CT-LE coupling value) with regard to the number of adiabatic input states.

To further assess the quality of the obtained diabatic states, we checked i) the eigenvalues resulting from the Multi-FED-FCD method, and ii) the character of the diabatic states. The latter is calculated by summing up the contributions from the transition density matrix in the atomic orbital basis, which lie on the respective fragments that characterize the kind of diabatic state of interest.[37] Specifically, the quality of diabatic states is computed as

$$X_{AA}(i) = \sum_{\mu \in A, \nu \in A} T^2_{\mu\nu}(i) \tag{3}$$

with T being the transition density matrix of diabatic state i in the atomic orbital basis with orbitals μ and ν. Index A indicates that the sum runs over atomic orbitals belonging to atoms of fragment A. Thus, X_{AA} represents the quality of a locally excited state of fragment A. Accordingly,

$$X_{AB}(i) = \sum_{\mu \in A, \nu \in B} T^2_{\mu\nu}(i) \tag{4}$$

computes the quality of a A^+B^- CT state.

The Multi-FED-FCD diabatization procedure[30] has been applied to obtain an initial set of diabatic states. The adiabatic-to-diabatic transformation obtained from the diabatization has been also applied to compute the diabatic electronic and magnetic transition dipole moments.

Perturbation theory for inclusion of CT states: resonant case We consider the special pair with local excited Q_y states $|\Psi_1\rangle = |P_{D1}Q_y\rangle$ and $|\Psi_2\rangle = |P_{D2}Q_y\rangle$, both with energy E_0. Because of the degeneracy, the excited state wavefunction will be delocalized and the adiabatic states read

$$|\Psi_1^{(ad)}\rangle = \frac{1}{\sqrt{2}}\left(|\Psi_1\rangle + |\Psi_2\rangle\right) \tag{5}$$

with energy $E_1^{(ad)} = E_0 + V_{12}^{diab}$ and

$$|\Psi_2^{(ad)}\rangle = \frac{1}{\sqrt{2}}\left(|\Psi_1\rangle - |\Psi_2\rangle\right) \tag{6}$$

with energy $E_2^{(ad)} = E_0 - V_{12}^{diab}$ where V_{12}^{diab} is the direct excitonic coupling between local excited Q_y states obtained from the diabatization.

Now, we switch on the coupling to CT states

$$\hat{V} = \sum_k (V_{1k}\,|\Psi_1\rangle\langle k| + V_{2k}\,|\Psi_2\rangle\langle k|) + h.c. \tag{7}$$

where, e.g., $V_{1k} = V_{P_{D1}Q_y,k\mathrm{CT}}$ is the coupling between the Q_y state of P_{D1} and the kth CT state, and h.c. denotes the hermitian conjugate.

In second-order perturbation theory, the following energies are obtained for the two adiabatic states

$$\begin{aligned} E_1 &= E_0 + V_{12}^{diab} + \sum_k \frac{|\langle \Psi_1^{(ad)}|V|k\rangle|^2}{E_1^{(ad)} - E_k} \\ &= E_0 + V_{12}^{diab} + \frac{1}{2}\sum_k \frac{V_{1k}V_{k1} + V_{2k}V_{k2} + V_{1k}V_{k2} + V_{2k}V_{k1}}{E_0 + V_{12}^{diab} - E_k} \end{aligned} \tag{8}$$

and

$$
\begin{aligned}
E_2 &= E_0 - V_{12}^{\mathrm{diab}} + \sum_k \frac{|\langle \Psi_2^{(\mathrm{ad})}|V|k\rangle|^2}{E_2^{(\mathrm{ad})} - E_k} \\
&= E_0 - V_{12}^{\mathrm{diab}} + \frac{1}{2}\sum_k \frac{V_{1k}V_{k1} + V_{2k}V_{k2} - V_{1k}V_{k2} - V_{2k}V_{k1}}{E_0 - V_{12}^{\mathrm{diab}} - E_k}
\end{aligned} \tag{9}
$$

Taking into account that $E_0 - E_k \pm V_{12}^{\mathrm{diab}} \approx E_0 - E_k$ and that the coupling elements are real ($V_{1k} = V_{k1}$ and $V_{2k} = V_{k2}$), the difference between the energies of adiabatic states becomes

$$
E_1 - E_2 = 2V_{12}^{\mathrm{diab}} + 2\sum_k \frac{V_{1k}V_{k2}}{E_0 - E_k} \tag{10}
$$

The splitting of eigenenergies in a homodimer is two times the coupling and, hence, we obtain the following coupling between $|\mathrm{P_{D1}Q_y}\rangle$ and $|\mathrm{P_{D2}Q_y}\rangle$

$$
V_{12} = V_{12}^{\mathrm{diab}} + \sum_k \frac{V_{1k}V_{k2}}{E_0 - E_k}\,, \tag{11}
$$

where V_{12}^{diab} is the direct coupling between local excited states (containing long-range Coulomb as well as non-CT-SR contributions) and the second term on the r.h.s. contains the superexchange-type coupling mediated by the coupling between local excited and CT states.

Perturbation theory for inclusion of CT states: non-resonant case We consider the special pair with local excited $\mathrm{Q_y}$ state of $\mathrm{P_{D1}}$, $|\Psi_1\rangle = |\mathrm{P_{D1}Q_y}\rangle$, with energy E_1 and a high-energy local excited state of $\mathrm{P_{D2}}$, $|\Psi_2\rangle = |\mathrm{P_{D2}}a\rangle$, with energy E_2. The coupling between these local excited states and the CT states (eq 7) is treated in perturbation theory up to second-order, giving for the adiabatic states

$$
|\Psi_1^{(\mathrm{ad})}\rangle \approx |\Psi_1\rangle + \sum_k \frac{V_{1k}}{E_1 - E_k}|k\rangle + \sum_k \frac{V_{1k}V_{k2}}{(E_1 - E_k)(E_1 - E_2)}|\Psi_2\rangle \tag{12}
$$

and

$$
|\Psi_2^{(\mathrm{ad})}\rangle \approx |\Psi_2\rangle + \sum_k \frac{V_{2k}}{E_2 - E_k}|k\rangle + \sum_k \frac{V_{2k}V_{k1}}{(E_2 - E_k)(E_2 - E_1)}|\Psi_1\rangle\,. \tag{13}
$$

Taking into account that the coupling matrix elements are real, that is, $V_{1k} = V_{k1}$ and $V_{2k} = V_{k2}$ we may express the above equations as

$$
|\Psi_1^{(\mathrm{ad})}\rangle \approx |\Psi_1\rangle + \sum_k \frac{V_{1k}}{E_1 - E_k}|k\rangle + \frac{V_{12}^{\mathrm{eff}}}{E_1 - E_2}|\Psi_2\rangle \tag{14}
$$

and

$$
|\Psi_2^{(\mathrm{ad})}\rangle \approx |\Psi_2\rangle + \sum_k \frac{V_{2k}}{E_2 - E_k}|k\rangle + \frac{V_{21}^{\mathrm{eff}}}{E_2 - E_1}|\Psi_1\rangle\,, \tag{15}
$$

where we have introduced the effective couplings

$$
V_{12}^{\mathrm{eff}} = \sum_k \frac{V_{1k}V_{k2}}{(E_1 - E_k)} \tag{16}
$$

and

$$V_{21}^{\text{eff}} = \sum_k \frac{V_{2k} V_{k1}}{(E_2 - E_k)} \,. \tag{17}$$

Obviously, it holds that $V_{12}^{\text{eff}} \neq V_{21}^{\text{eff}}$. In order to restore the required symmetry, we determine an energy difference ΔE such that its inverse has minimal deviations from both $1/(E_1 - E_k)$ and $1/(E_2 - E_k)$, occurring in V_{12}^{eff} and V_{21}^{eff}, respectively. Hence, we have

$$(\frac{1}{\Delta E} - \frac{1}{E_1 - E_k})^2 + (\frac{1}{\Delta E} - \frac{1}{E_2 - E_k})^2 \rightarrow \text{min.} \tag{18}$$

The solution for $1/\Delta E$ reads

$$\frac{1}{\Delta E} = \frac{1}{2}\left(\frac{1}{E_1 - E_k} + \frac{1}{E_2 - E_k}\right) \tag{19}$$

and the effective coupling is obtained as

$$V_{12}^{\text{eff}} = \sum_k \frac{V_{1k} V_{k2}}{2}\left(\frac{1}{E_1 - E_k} + \frac{1}{E_2 - E_k}\right) \tag{20}$$

If one of the two states, say $|\Psi_2\rangle$ has an energy E_2 that is not off-resonant enough from the CT state, we can only give a lower bound to V_{12}^{eff} by setting $E_2 = E_1$ in the above equation, resulting in

$$V_{12}^{\text{eff}} \geq \sum_k \frac{V_{1k} V_{k2}}{E_1 - E_k} \tag{21}$$

The term on the r.h.s. is used as an estimate of the CT contribution to the excitonic coupling $V_{\mathrm{P_{D1}P_{D2}}}^{(\mathrm{Q_y},a)}$ between local excited $\mathrm{Q_y}$ state of $\mathrm{P_{D1}}$ and the ath high-energy excited state of $\mathrm{P_{D2}}$ in eq 3 of the main text.

5.2 Results

Diabatic Hamiltonian The full Hamiltonian of the special pair as obtained from the diabatization is provided as an additional file "SP_Hamiltonian_diab.xlsx". The values for the CT states and the Q_y states are reported in Table S4. The energies of the lowest CT states are found to be around 10000 cm^{-1} higher than the $\mathrm{Q_y}$ states. However, relatively large couplings of up to 1127 cm^{-1} suggest a potential impact of the CT states on the $\mathrm{Q_y}$ states. Indeed, the site energies after perturbation by CT states (eq 2 of the main text) have been computed to be 15919 cm^{-1} and 15922 cm^{-1} for $\mathrm{P_{D1}}$ and $\mathrm{P_{D2}}$, respectively, as compared to 16079 cm^{-1} and 16078 cm^{-1}, respectively, without the perturbation (Table S5). The coupling between the $\mathrm{Q_y}$ states of $\mathrm{P_{D1}}$ and $\mathrm{P_{D2}}$ is increased from 137 cm^{-1} to 235 cm^{-1} by the coupling to CT states (eq 3 of the main text with $a = \mathrm{Q_y}$). Note, that the full Hamiltonian after perturbation by CT states is provided as an additional file ("SP_Hamiltonian_pert.xlsx"). Furthermore, an analysis of the character of the first two adiabatic states according to eq 3- 4 yields a composition of CT1: 2.6%, CT2: 2.5%, LE1: 47.9%, LE2: 46.9% for the first adiabatic state and a composition of CT1: 1.7%, CT2: 1.7%, LE1: 46.5%, LE2: 50.0% for the second adiabatic state.

In order to assess the quality of the obtained CT states (cf. Table S4), their character has been computed according to eq 4 as 95%, 93%, 82%, and 89% for the CT1 states, and 93%, 92%, 91%, and 88% for the CT2 states. Thus, although yielding diabatic states of high quality, the diabatization procedure cannot provide strictly separated diabatic states. The latter result justifies to include the effects of the quasi CT states perturbatively for the respective energies, couplings, and dipole moments of the $\mathrm{Q_y}$ states despite strict CT states would be optically dark and, thus, should not affect the electric transition dipoles. However,

Table S4: Excerpt of diabatic Hamiltonian comprising the four lowest CT states $P_{D1}^{-}P_{D2}^{+}$ (CT1) and $P_{D1}^{+}P_{D2}^{-}$ (CT2), as well as the Q_y states of P_{D1} (LE1) and P_{D2} (LE2) (in units of cm^{-1}) .

	**1.CT1**	**2.CT1**	**3.CT1**	**4.CT1**	**1.CT2**	**2.CT2**	**3.CT2**	**4.CT2**	**1.LE1**	**1.LE2**
**1.CT1**	27113	0	0	0	3	-31	-17	4	613	-1127
**2.CT1**	0	30841	0	0	37	43	16	-19	-597	-308
**3.CT1**	0	0	36679	0	-49	-52	31	-105	552	-179
**4.CT1**	0	0	0	37051	22	18	3	-1	166	218
**1.CT2**	3	37	-49	23	25495	0	0	0	855	-524
**2.CT2**	-31	43	-52	18	0	28841	0	0	30	4
**3.CT2**	-17	16	31	3	0	0	34419	0	-392	-148
**4.CT2**	4	-19	-105	-1	0	0	0	34683	5	-54
**1.LE1**	613	-597	552	166	855	30	-392	5	16079	137
**1.LE2**	-1127	-308	-179	218	-524	4	-148	-54	137	16078

Table S5: Q_y transition energies (in cm^{-1}) as obtained from quantum chemistry calculations on (i) isolated monomers, (ii) dimers, after diabatization, (iii) dimers, after diabatization and perturbation by CT-states (eq 2 of the main text) in vacuum and by including the protein environment as point charges (for (ii) and (iii)).

	**Monomer**	**Diabatization**		**Pert. by CT-states**	
Environment	vacuum	vacuum	protein	vacuum	protein
$\mathbf{P_{D1}}$	16051	16119	16079	15968	15919
$\mathbf{P_{D2}}$	16017	16062	16078	15909	15922

as stated in the main text, we do not find the effects of the CT states to be the main cause for the observed non-conservativity in the CD-spectra.

Excitation energies The Q_y transition energies (site energies) obtained from quantum chemistry calculations on isolated monomers and (after diabatization) from dimers are further analyzed regarding the impact of the environment. The results are summarized in Table S5. Vacuum calculations are compared with calculations were the protein environment was included in the quantum chemical calculations by point charges (electrostatic embedding). In vacuum, the site energies obtained after diabatization are blue-shifted by about 60 cm^{-1} with respect to the values obtained for the isolated monomers. Including the perturbation by CT states (eq 2 of the main text) leads to a 150 cm^{-1} red shift. Hence, in vacuum the non-CT-SR effects and the CT coupling effects partially compensate each other. Including the electrostatic coupling to the protein environment adds a 50 cm^{-1} red shift to P_{D1} and a 15 cm^{-1} blue shift to P_{D2}, such that the site energies of the two pigments are in resonance. The overall effect of CT state coupling, non-CT-SR and long-range electrostatic effects on the site energies of the special pair are small.

Poisson-TrEsp couplings The long-range couplings between special pair states have been computed using the Poisson-TrEsp method.[38,39] This method takes into account the polarizability of the protein environment by that of a homogeneous dielectric with an average optical dielectric constant of $\epsilon_{opt} = 2$, estimated from an analysis of the oscillator strength of Chl *a* bound to photosystem I.[40] The required atomic partial transition charges for the special pair are obtained from quantum chemical calculations on the isolated P_{D1} and P_{D2} monomers. The transition densities between the ground state and the first 5 LE states were calculated using TDDFT with the ωB97X-D3 XC functional and a def2-SVP basis set, using Q-Chem.[41] From a fit of the ESP of the transition densities, the respective atomic transition charges were obtained. The MEAD software tool[ii] has been used for the solution of the Poisson equation for the electrostatic potential of the transition charges used

[ii] https://rtullmann.de/index.php?name=extended-mead

Table S6: Excitonic couplings between Q_y state of P_{D1} and five lowest excited states of P_{D2} ($a = 1, ..., 5$) ; $V^{(LR,qc)}_{P_{D1}P_{D2}}(Q_y, a)$: long-range excitonic coupling based on transition charges of quantum chemical calculations; $V^{(LR,pp)}_{P_{D1}P_{D2}}(Q_y, a)$: post-processed long-range excitonic couplings considering rescaling of electronic transition dipole moments as well as polarization effects in the environment;$V^{diab}_{P_{D1}Q_y,P_{D2}a}$: excitonic couplings obtained from diabatization (i.e., including non-CT-SR effects); $V^{(Q_y,a)}_{P_{D1}P_{D2}}$(qc): overall excitonic coupling after perturbation by CT-states (eq 3 of the main text); $V^{(Q_y,a)}_{P_{D1}P_{D2}}$(pp): overall post-processed excitonic coupling values obtained as $V^{(Q_y,a)}_{P_{D1}P_{D2}}$(qc)- $V^{(LR,qc)}_{P_{D1}P_{D2}}(Q_y, a)$ + $V^{(LR,pp)}_{P_{D1}P_{D2}}(Q_y, a)$

a	$V^{(LR,qc)}_{P_{D1}P_{D2}}(Q_y, a)$	$V^{(LR,pp)}_{P_{D1}P_{D2}}(Q_y, a)$	$V^{diab}_{P_{D1}Q_y,P_{D2}a}$	$V^{(Q_y,a)}_{P_{D1}P_{D2}}$(qc)	$V^{(Q_y,a)}_{P_{D1}P_{D2}}$(pp)
1 (Q_y)	149	107	137	235	193
2 (Q_x)	20	7	23	128	115
3	180	106	148	180	106
4	29	38	-92	-176	-167
5	253	155	241	335	237

for the coupling calculations. The transition charges of the LE states of the special pair have been rescaled to correct for uncertainties in the quantum chemical transition dipole moments, based on experimental oscillator strengths of Chl *a*, as it has been conducted in ref.[42] On the one hand, the scaling factor for the Q_y states is obtained by a comparison of the vacuum transition dipole moments of the monomers with the value of 4.58 D for the Q_y transition of chlorophyll *a* obtained by Knox & Spring[43] from an analysis of experimental oscillator strengths measured in different solvents. The scaling factor for the higher excited states on the other hand results from a comparison of calculated transition dipole strengths relative to that calculated for the Q_y transition and corresponding areas of the absorption spectrum.[42] For the present quantum chemical calculations we obtain scaling factors that lead to calibrated vacuum transition dipole moments of 4.58 D (1.LE), 6.99 D (3.LE), 6.93 D 4.LE) and 4.38 D (5. LE). In the present work we include the Q_x-transition (2.LE) of the Chls in the special pair. An experimental vacuum transition dipole moment of 1.47 D has been assumed for this transition, based on an estimate by Reimers *et al.*[44] For the reaction field factor, introduced recently into the Poisson-TrEsp method,[45] we use the lower limit 1.0, since it provides a better fit of the experimental CD spectrum (Fig. S10). The long-range contributions to overall excitonic couplings in Eq. 3 of the main text, obtained with unscaled transition charges from the diabatization and subsequent perturbation theory, were then corrected by subtracting the vacuum coupling obtained from the unscaled transition charges and adding the above Poisson-TrEsp values obtained for rescaled transition charges and in the presence of a dielectric medium. This procedure yields coupling values between Q_y and higher excited states which are summarized in Tables S6 and S7, where long-range contributions are provided together with the respective couplings after diabatization and perturbation by CT states (eq 3 of the main text). Whereas the excitonic coupling between Q_y transitions is strongly enhanced by the coupling to CT states, the excitonic coupling between the 3rd LE state of one special pair pigment and the Q_y transition of the other is barely affected by SR effects. In two cases, $V^{(Q_y,4)}_{P_{D1}P_{D2}}$ and $V^{(Q_x,Q_y)}_{P_{D1}P_{D2}}$ we find a sign inversion of the overall coupling by SR effects.

Fluctuations of Hamiltonian parameters As outlined in the main text, we monitored the change in the parameters of the special pair Hamiltonian along 110 MD snapshots, that were each geometry-optimized and analyzed by our quantum chemical/diabatization procedure. Examples of distribution functions obtained from these analyzes are shown in Fig. S6, where the Q_y transition energies of P_{D1} and P_{D2} (eq 2 of the main text) as well as the coupling $V^{(Q_y,Q_y)}_{P_{D1}P_{D2}}$ between Q_y transitions, obtained after diabatization with and without including the perturbation by CT states are investigated. The mean values of the transition energies and

Table S7: same as Table S6 but between Q_y state of P_{D2} and higher excited states of P_{D1}

a	$V^{(LR,qc)}_{P_{D1}P_{D2}}(a, Q_y)$	$V^{(LR,pp)}_{P_{D1}P_{D2}}(a, Q_y)$	$V^{diab}_{P_{D1}a,P_{D2}Q_y}$	$V^{(a,Q_y)}_{P_{D1}P_{D2}}(qc)$	$V^{(a,Q_y)}_{P_{D1}P_{D2}}(pp)$
1	149	107	137	235	193
2	-66	-22	-24	-5	39
3	-176	-101	-144	-181	-106
4	127	59	140	165	97
5	-221	-146	-223	-273	-198

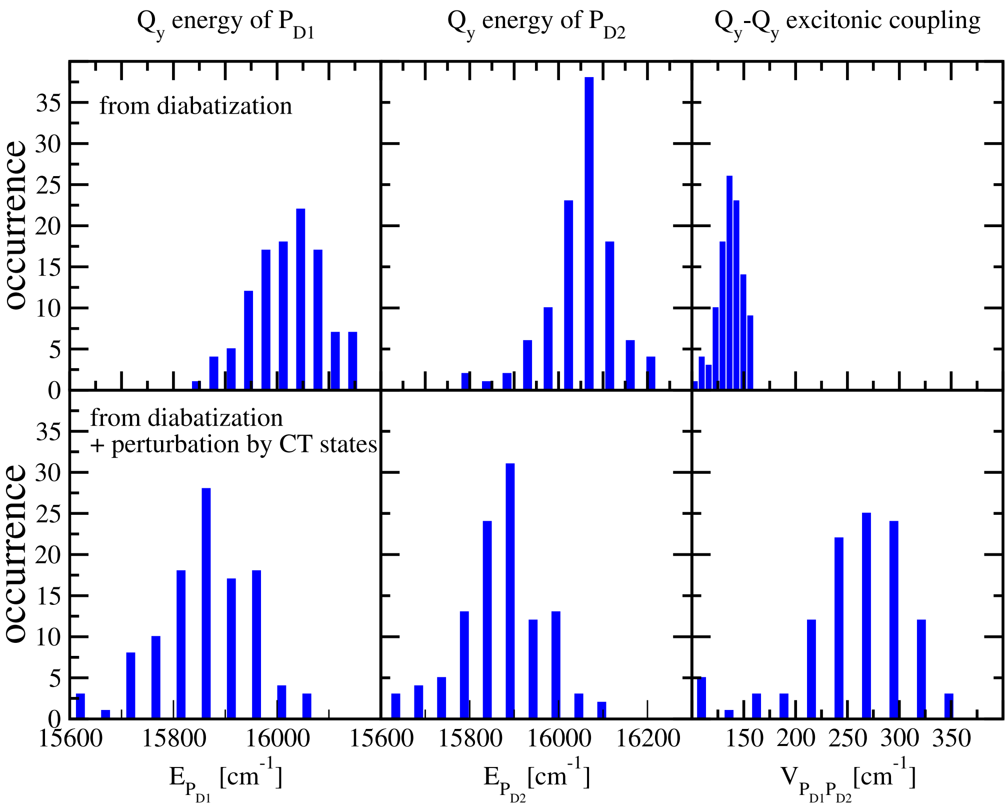

Figure S6: Histograms for site energies and couplings of Q_y states based on snapshot analysis with (left) and without (right) perturbation by CT states; top left: P_{D1} site energy $E^{diab}_{P_{D1}Q_y}$, top right: $E^{(Q_y)}_{P_{D1}}$ (eq 2 of the main text), middle left : same as top but for P_{D2}, bottom left: $V^{diab}_{P_{D1}Q_y,P_{D2}Q_y}$, bottom right: $V^{(Q_y,Q_y)}_{P_{D1}P_{D2}}$ (eq 3 of the main text).

Table S8: Q_y transiton energies of the special pair pigments as well as excitonic couplings between Q_y transitions, obtained after diabatization and including a perturbation by CT states (eqs 2 and 3 of the main text for site energies and couplings, respectively). The values obtained for the representative structure are compared with the mean values of the 110 MD snapshots.

	$E^{diab}_{P_{D1}Q_y}$	$E^{diab}_{P_{D2}Q_y}$	$E^{(Q_y)}_{P_{D1}}$	$E^{(Q_y)}_{P_{D2}}$	$V^{diab}_{P_{D1}Q_y,P_{D2}Q_y}$	$V^{(Q_y,Q_y)}_{P_{D1}P_{D2}}$
Mean over snapshots	16038	16073	15885	15899	133	243
Representative structure	16079	16078	15919	15922	137	235

couplings are very close to the transition energies obtained for the representative structure (Table S8). In the calculation of spectra, the distribution functions are approximated by Gaussians with standard deviations obtained from the snapshots. Note, that the snapshot analysis has been performed based on 20 adiabatic input states, whereas 30 adiabatic input states have been used for the representative structure calculations. Although the histograms in Figure S6 roughly indicate a Gaussian distribution, the possibility of having multiple possible conformations for the special pair cannot be eliminated completely with this relatively small sample size. The standard deviation in units of cm^{-1} of the energy fluctuations of the diabatic transition energies $E^{\mathrm{diab}}_{\mathrm{P_{D1}}a}$ and $E^{\mathrm{diab}}_{\mathrm{P_{D2}}a}$ of the first $a = 1...5$ LE states are obtained as.

- $E^{\mathrm{diab}}_{\mathrm{P_{D1}}a}$: 68 (160), 100 (235), 168 (396), 322 (758), 158 (372)
- $E^{\mathrm{diab}}_{\mathrm{P_{D2}}a}$: 76 (179), 128 (301), 133 (313), 297 (699), 160 (377)

where the FWHM values (in cm^{-1}) are given in brackets. Taking into account the coupling to CT states (main text, eq 2) leads to an increase of the variation to 92 cm^{-1} (217 cm^{-1} FWHM) and 94 cm^{-1} (221 cm^{-1} FWHM) for the Q_y state of the P_{D1} and P_{D2} pigments, respectively.

The standard deviations in diabatic couplings $V^{\mathrm{diab}}_{\mathrm{P_{D1}Q_y,P_{D2}}a}$ and $V^{\mathrm{diab}}_{\mathrm{P_{D1}}a\mathrm{,P_{D2}Q_y}}$ between Q_y state and the ath LE state of the other special pair pigment (FWHM in brackets, all in units of cm^{-1}) are obtained as

- $V^{\mathrm{diab}}_{\mathrm{P_{D1}Q_y,P_{D2}}a}$: 13 (31), 8 (19), 29 (68), 62 (146), 35 (82)
- $V^{\mathrm{diab}}_{\mathrm{P_{D1}}a\mathrm{,P_{D2}Q_y}}$: 13 (31), 7 (16), 41 (97), 49 (115), 19 (45)

Taking into account the coupling via CT states (eq 3 of the main text) results in the couplings $V^{(\mathrm{Q_y},a)}_{\mathrm{P_{D1}P_{D2}}}$ and $V^{(a,\mathrm{Q_y})}_{\mathrm{P_{D1}P_{D2}}}$ (eq 3 of the main text), the standard deviations of which (FWHM in brackets, all in units of cm^{-1}) are obtained as

- $V^{(\mathrm{Q_y},a)}_{\mathrm{P_{D1}P_{D2}}}$: 53 (125), 28 (66), 35 (82), 88 (207), 44 (104)
- $V^{(a,\mathrm{Q_y})}_{\mathrm{P_{D1}P_{D2}}}$: 53 (125), 20 (47), 49 (115), 65 (153), 23 (54).

Additionally, the explicit fluctuations of CT state energies have been computed to be $\approx$1550 cm^{-1} and the fluctuations of the coupling between CT states and LE states are in the range of 4-230 cm^{-1}. The latter fluctuations are about 5 times higher than the fluctuations of the direct LE couplings. In particular, variations of LE-LE couplings between Q_y transitions are about 4 times higher when taking into account their indirect coupling via CT states, as seen also in the right panels of Fig. S6.

6 Computation of CD-spectra

6.1 Method

6.1.1 Embedding the Special Pair Hamiltonian into the Reaction Center Hamiltonian

The long-range excitonic couplings between special pair states and LE states of the other reaction center pigments have been computed using the Poisson-TrEsp method,[38,39] described above. The required atomic partial transition charges for the special pair are calculated based on the diabatic transition densities and are determined with Mulliken partitioning, whereas the transition charges of other reaction center pigments are taken from prior work.[1] The coupling values between as well as transition energies of excited states which do not involve the special pair pigments have been taken from ref.[1] Note that we have used different transition charges for the intra special pair couplings, described above, since in the latter case we wanted

Table S9: Q_y transition energies (in units of cm^{-1}) of the present full model are compared to those of our earlier work.[4] Please note that the latter values are used also in the minimal model, introduced in the present work.

**Pigment**	**full model**	**ref[4]**
P_{D1}	14975	14970
P_{D2}	14975	14970
Chl_{D1}	14732	14749
Chl_{D2}	14952	14925
$Pheo_{D1}$	14985	14925
$Pheo_{D2}$	14820	14815
$Chlz_{D1}$	14975	14975
$Chlz_{D2}$	15042	15038

to avoid artefacts due to the fact that the diabatic transition densities are not perfectly localized. For the couplings between special pair pigments and the other RC pigments, these effects are negligible.

The system-field interaction is characterized by the electric transition dipole moments $\boldsymbol{\mu}_{ma}$ of LE states. For the non-special pair pigments, these transition dipole moments refer to those of monomeric Chl *a*, Pheo *a* and Car, as described previously.[1] For the special pair pigments, we use the transition dipole moments of the diabatic states instead. For the Q_y transition of the special pair pigments, in addition, the coupling to CT states is included (main text, eq 9). The diabatic electronic transition dipole moments have been scaled for the Q_y-region and the Soret-band, respectively, as it has been conducted in ref.[42] The atomic transition charges for the Poisson-TrEsp calculations for couplings between special pair states and other RC pigments have been scaled analogously. The scaling factors for the transition dipole moments have been calculated to be 0.840 (Q_y of P_{D1}), 0.857 (Q_y of P_{D2}), 0.905 (higher excited LE states of P_{D1})), and 0.912 (higher excited LE states of P_{D2})). In order to keep the scaling procedure consistent, we keep only the first 5 LE states. This truncation has no significant effect on the results based on rotational strength calculations of the special pair. Regarding the other reaction center pigments, the Q_y-, B_y-, B_x, and N_{x+xy} are considered for the 4 chlorophyll *a* and pheophytin *a* pigments. Additionally, four excited states of the β-carotene (Car) are taken into account, corresponding to the 0-0. 0-1, 0-2, and 0-3 vibronic transitions between the electronic ground state and the first dipole-allowed excited state (S_2).

The coupling between Q_y transitions in the special pair is found to be 193 cm^{-1} (see above and eq 4 of the main text). In the calculation of the CD spectra, we nevertheless use a value of 150 cm^{-1} that has been inferred from a fit of optical spectra.[46] Indeed, we find that the non-conservativity of the CD spectrum obtained with this coupling is slightly closer to the experimental value. An assessment regarding the influence of this coupling value on the CD spectrum is provided in Fig. S8. Prior to the computation of optical spectra, the special pair structure has been embedded into the crystal structure using the Kabsch algorithm.[47]

As quantum chemical methods are known to yield too high excitation energies, they have been shifted a posteriori. The Q_y transition energies of the special pair pigments P_{D1} and P_{D2} have been shifted to resemble the values from our earlier studies,[1,4] and the transition energies of the higher LE states are shifted by the same constant (950 cm^{-1}). Finally, the site energy values for the reaction center pigments obtained from the literature[1,4] come with a tolerance of ± 60 cm^{-1}. Therefore, a final manual fit of Q_y transition energies (site energies) has been conducted for the final CD-spectrum using the full model. Please find a comparison between the refined site energies and those of the literature (that correspond to that of the present minimal model) in Table S9. The special pair excitation energies used for the calculation of spectra are for P_{D1}: 14975 cm^{-1}, 18934 cm^{-1}, 26690 cm^{-1}, 28242 cm^{-1}, 29251 cm^{-1}; and for P_{D2}: 14975 cm^{-1}, 19215 cm^{-1}, 26357 cm^{-1}, 28402 cm^{-1}, 28954 cm^{-1}. Note, that the full reaction center Hamiltonian is provided as an additional excel

file "RC_Hamiltonian.xlsx".

6.1.2 Theory of Circular Dichroism Spectra

The transition of the system from the ground state to a one-exciton state is caused by couplings to the fields of the electromagnetic wave with frequency ω and is included as $H_{\mathrm{ex-rad}}$ into the overall Hamiltonian

$$H = H_{\mathrm{ex}} + H_{\mathrm{ex-vib}} + H_{\mathrm{vib}} + H_{\mathrm{ex-rad}}, \tag{22}$$

where the exciton-radiation interaction Hamiltonian is defined in the rotating wave approximation[48] as

$$H_{\mathrm{ex-rad}} = \sum_m h_{m0} e^{-i\omega t} + \mathrm{h.c.} \tag{23}$$

with

$$h_{m0} = \frac{eA_0}{2m_e c} \langle m | \sum_j e^{i\mathbf{k}\cdot\hat{\boldsymbol{r}}_j^{(m)}} \boldsymbol{\epsilon} \cdot \hat{\boldsymbol{p}}_j^{(m)} |0\rangle \,, \tag{24}$$

where $\hat{\boldsymbol{r}}_j^m$ is the coordinate of the j-th electron of pigment m, $\hat{\boldsymbol{p}}_j^m = -i\hbar\nabla_j$ is its momentum, m_e is its mass, $A_0 k = E_0 = B_0$ (with $k = \frac{\omega}{c}$) are the amplitudes of the incoming electromagnetic field, and $\boldsymbol{\epsilon}$ is its polarization unit vector. The light propagates in the direction of $\boldsymbol{n} = \frac{\boldsymbol{k}}{k}$.

The rate constant for the transition to the Mth exciton state is given by Fermi's golden rule as

$$k_{0\to M} = \frac{2\pi}{\hbar^2} \left| \sum_m c_m^{(M)} h_{m0} \right|^2 \delta(\omega - \omega_{M0}) \tag{25}$$

with eigenvalues of H_{ex} given by $E_M = \hbar\omega_{M0}$. The δ-distribution is replaced by a line shape function $D_M(\omega)$ as a result of the exciton-vibrational couplings. For the calculation of optical spectra, the exciton-vibrational coupling as well as the coupling of the RC to external light fields has to be taken into account. As described previously,[1] the exciton-vibrational coupling gives rise to a line shape function of optical transitions that contains the excitation of vibrational sidebands and a lifetime broadening due to exciton relaxation. As before, we use a line shape function obtained with a partial ordering prescription (POP) cumulant expansion approach and a spectral density $J(\omega)$ that has been extracted from fluorescence line narrowing spectra of the B777 pigment-protein complex.[49] The line shape function reads[50]

$$D_M(\omega) = \frac{1}{\pi} \mathrm{Re}[\int_0^\infty dt e^{i(\omega-\omega_M-\Delta\omega_M)t} e^{F_{MM}(t)-F_{MM}(0)} e^{-t/\tau_M}], \tag{26}$$

where τ_M describes the lifetime broadening of the exciton states, and $\Delta\omega_M$ a frequency shift as defined in ref.[50] The function $F_{MM}(t)$ depends on the generalized spectral density $J_{MNKL}(\omega) = \sum_\xi g_\xi(M,N) g_\xi(K,L) \delta(\omega - \omega_\xi)$. The latter is commonly approximated to be of the form[49]

$$J_{MNKL}(\omega) = S J_0(\omega) \sum_m c_m^{(M)} c_m^{(N)} c_m^{(K)} c_m^{(L)} \tag{27}$$

with the Huang-Rhys factor S and the normalized function ($\int_0^\infty d\omega J_0(\omega) = 1$)

$$J_0(\omega) = \frac{1}{s_1 + s_2} \sum_{i=1,2} \frac{s_i}{7!2\omega_i^4} \omega^3 e^{-(\omega/\omega_i)^{1/2}} \tag{28}$$

with parameters $s_1 = 0.8$, $s_2 = 0.5$, $\hbar\omega_1 = 0.069$ meV, and $\hbar\omega_2 = 0.24$ meV.[49] The shape of the spectral

density has been found to be very similar for different pigment-protein complexes, whereas the Huang-Rhys factor may be different.[3] The latter can be obtained from the temperature dependence of the linear absorbance, where $S = 0.65$ was determined for the PSII RC.[51] Note that the fluctuations of excitonic couplings and the correlations in site energy fluctuations can be neglected, based on an earlier normal mode analysis of the spectral density.[52,53]

In order to calculate the rate constants $k_{0\to M}$, the matrix elements h_{m0} are determined using a multipole expansion[54] in the wave vector $\boldsymbol{k}$. This approximation yields up to first order $h_{m0} \approx h_{m0}^{(0)} + h_{m0}^{(1)}$ with the zeroth-order contribution

$$h_{m0}^{(0)} = \frac{eA_0}{2m_e c}\langle m| \sum_i \boldsymbol{\epsilon} \cdot \hat{\boldsymbol{p}}_i^{(m)} |0\rangle \tag{29}$$

and the first-order term

$$h_{m0}^{(1)} = i\frac{eA_0}{2m_e c}\langle m| \sum_i (\boldsymbol{\epsilon} \cdot \hat{\boldsymbol{p}}_i^{(m)})(\boldsymbol{k} \cdot \hat{\boldsymbol{r}}_i^{(m)}) |0\rangle. \tag{30}$$

Using the relation

$$\langle m|\hat{\boldsymbol{p}}_i^{(m)}|0\rangle = i\frac{E_m m_e}{\hbar}\langle m|\hat{\boldsymbol{r}}_i^{(m)}|0\rangle \tag{31}$$

the zeroth-order contribution becomes

$$h_{m0}^{(0)} = i\frac{A_0}{2\hbar c}E_m(\boldsymbol{\epsilon}\cdot\boldsymbol{\mu_m}) = i\frac{E_0 E_m}{2\hbar\omega}(\boldsymbol{\epsilon}\cdot\boldsymbol{\mu_m}) \tag{32}$$

with $\boldsymbol{\mu_m} = \langle m| \sum_i e\hat{\boldsymbol{r}}_i^{(m)}|0\rangle$ being the electric transition dipole moment of pigment m in so-called length gauge. The first-order contribution splits into two parts which are symmetric and antisymmetric with respect to the interchange of $\boldsymbol{k}$ and $\boldsymbol{\epsilon}$ as

$$h_{m0}^{(1)} = h_{m0}^{(1,m)} + h_{m0}^{(1,Q)}. \tag{33}$$

Using the vector identity

$$(\boldsymbol{a}\cdot\boldsymbol{c})(\boldsymbol{b}\cdot\boldsymbol{d}) - (\boldsymbol{b}\cdot\boldsymbol{c})(\boldsymbol{a}\cdot\boldsymbol{d}) = (\boldsymbol{a}\times\boldsymbol{b})\cdot(\boldsymbol{c}\times\boldsymbol{d}) \tag{34}$$

and the definition of the magnetic transition dipole moment

$$\boldsymbol{m}_m = \frac{e}{2m_e c}\langle m| \sum_i (\hat{\boldsymbol{r}}_i^{(m)} \times \hat{\boldsymbol{p}}_i^{(m)}|0\rangle \tag{35}$$

the first contribution on the r.h.s. of (33) becomes

$$h_{m0}^{(1,m)} = i\frac{E_0}{2}(\boldsymbol{n}\times\boldsymbol{\epsilon})\cdot\boldsymbol{m}_m. \tag{36}$$

The second contribution $h_{m0}^{(1,Q)}$ results from the electric quadrupole moment and reads

$$\begin{aligned} h_{m0}^{(1,Q)} &= i\frac{eA_0}{4m_e c}\sum_i\sum_{\alpha,\beta} k_\alpha \epsilon_\beta \langle m|\hat{r}_{i,\alpha}^{(m)}\hat{p}_{i,\beta}^{(m)} + \hat{r}_{i,\beta}^{(m)}\hat{p}_{i,\alpha}^{(m)}|0\rangle = \\ &= i\frac{eA_0}{4m_e c}\sum_i\sum_{\alpha,\beta} k_\alpha\epsilon_\beta \frac{i m_e E_m}{\hbar}\langle m|\hat{r}_{i,\alpha}^{(m)}\hat{r}_{i,\beta}^{(m)}|0\rangle = -\frac{E_m}{\hbar\omega}\frac{E_0}{4}\sum_{\alpha,\beta} k_\alpha \epsilon_\beta Q_m^{(\alpha\beta)}, \end{aligned} \tag{37}$$

where in the last step the definition of the quadrupole operator $\hat{Q}_m^{(\alpha\beta)} = e\sum_i \hat{r}_{i,\alpha}^{(m)}\hat{r}_{i,\beta}^{(m)}$ has been used and $\hat{r}_{i,\alpha}^{(m)}$ denotes the α-th Cartesian coordinate of $\hat{\boldsymbol{r}}_{\boldsymbol{i}}^{(\boldsymbol{m})}$.

Next, relative coordinates are introduced as

$$\boldsymbol{r}_{\boldsymbol{i}}^{(\boldsymbol{m})} = \boldsymbol{R}_m + \boldsymbol{r}_i'^{(m)}, \tag{38}$$

where $\boldsymbol{R}_m$ denotes the center position of pigment m and $\boldsymbol{r}_i'^{(m)}$ the relative coordinate with respect to this center. Using this transformation, the magnetic transition dipole becomes

$$\boldsymbol{m}_m = \frac{e}{2m_e c}(\boldsymbol{R}_m \times \langle m| \sum_i \hat{\boldsymbol{p}}_i^{(m)}|0\rangle) + \boldsymbol{m}_m^{(\text{intr})} = \frac{iE_m}{2\hbar c}(\boldsymbol{R}_m \times \boldsymbol{\mu}_m) + \boldsymbol{m}_m^{(\text{intr})} \tag{39}$$

with the intrinsic magnetic transition dipole $\boldsymbol{m}_m^{(\text{intr})} = \frac{e}{2m_e c}\langle m| \sum_i (\hat{\boldsymbol{r}}_i'^{(m)} \times \hat{\boldsymbol{p}}_i^{(m)}|0\rangle$. The transition quadrupole moment is transformed as

$$Q_m^{(\alpha\beta)} = R_m^{(\alpha)}\mu_m^{(\beta)} + R_m^{(\beta)}\mu_m^{(\alpha)} + Q_m'^{(\alpha\beta)} \tag{40}$$

with the intrinsic transition quadrupole moment $Q_m'^{(\alpha\beta)} = \langle m|e\sum_i \hat{r}_{i,\alpha}'^{(m)}\hat{r}_{i,\beta}'^{(m)}|0\rangle$.

Inserting the expressions for the magnetic transition dipole as well as the electric transition quadrupole into (36) and (37) yields, using (34)

$$\begin{aligned} h_{m0}^{(1,m)} &= i\frac{E_0}{2}(\boldsymbol{n} \times \boldsymbol{\epsilon}) \cdot (\frac{iE_m}{2\hbar c}\boldsymbol{R}_m \times \boldsymbol{\mu}_m + \boldsymbol{m}_m^{(\text{intr})}) = \\ &= -\frac{E_0 E_m}{4\hbar c}((\boldsymbol{n} \cdot \boldsymbol{R_m})(\boldsymbol{\epsilon} \cdot \boldsymbol{\mu}_m) - (\boldsymbol{\epsilon} \cdot \boldsymbol{R_m})(\boldsymbol{n} \cdot \boldsymbol{\mu}_m)) + i\frac{E_0}{2}(\boldsymbol{n} \times \boldsymbol{\epsilon}) \cdot \boldsymbol{m}_m^{(\text{intr})} \end{aligned} \tag{41}$$

and

$$h_{m0}^{(1,Q)} = -\frac{E_m}{\hbar\omega}\frac{E_0}{4}((\boldsymbol{R}_m \cdot \boldsymbol{k})(\boldsymbol{\epsilon} \cdot \boldsymbol{\mu}_m) + (\boldsymbol{R}_m \cdot \boldsymbol{\epsilon})(\boldsymbol{k} \cdot \boldsymbol{\mu}_m)) + \sum_{\alpha,\beta} k_\alpha \epsilon_\beta Q_m'^{(\alpha\beta)}. \tag{42}$$

It has been shown in ref[48] that the contribution of the intrinsic transition quadrupole vanishes for CD spectra of isotropic samples. As we only investigate spectra of the latter kind, that contribution is neglected already at the present stage, and the overall first-order contribution is obtained from eqs (33), (41), and (42) as

$$h_{m0}^{(1)} = -\frac{E_0 E_m}{2\hbar\omega}(\boldsymbol{n} \cdot \boldsymbol{R_m})(\boldsymbol{\epsilon} \cdot \boldsymbol{\mu}_m) + i\frac{E_0}{2}(\boldsymbol{n} \times \boldsymbol{\epsilon}) \cdot \boldsymbol{m}_m^{(\text{intr})}. \tag{43}$$

Using the multipole extension, mentioned above, the rate constant in eq (25) is approximated as

$$k_{0\to M} \approx k_{0\to M}^{(0)} + k_{0\to M}^{(1)} \tag{44}$$

with the zeroth-order part

$$\begin{aligned} k_{0\to M}^{(0)} &= \frac{2\pi}{\hbar^2}\left|\sum_m c_m^{(M)} h_{m0}^{(0)}\right|^2 D_M(\omega - \omega_{M0}) = \frac{\pi E_0^2}{2\hbar^4\omega^2}\left|\sum_m c_m^{(M)} E_m \boldsymbol{\epsilon} \cdot \boldsymbol{\mu}_m\right|^2 D_M(\omega - \omega_{M0}) = \\ &= \frac{\pi E_0^2}{2\hbar^4\omega^2}(\sum_{m,n} c_m^{(M)} c_n^{(M)} E_m E_n (\boldsymbol{\epsilon}^* \cdot \boldsymbol{\mu}_m)(\boldsymbol{\epsilon} \cdot \boldsymbol{\mu}_n))D_M(\omega - \omega_{M0}). \end{aligned} \tag{45}$$

The first-order contribution to the rate constant is given by

$$k_{0\to M}^{(1)} = \frac{2\pi}{\hbar^2}\sum_{m,n} c_m^{(M)} c_n^{(M)} (h_{n0}^{(0)} h_{m0}^{*(1)} + h_{n0}^{*(0)} h_{m0}^{(1)}) D_M(\omega - \omega_{M0}). \tag{46}$$

Inserting (32) and (43) yields

$$k^{(1)}_{0\to M} = \frac{E_0^2\pi}{2\hbar^2}\sum_{m,n} c_m^{(M)} c_n^{(M)} \,\mathrm{Re}\left\{ i\frac{E_n}{\hbar\omega}(\boldsymbol{\epsilon}^* \cdot \boldsymbol{\mu}_n)\left(\frac{E_m}{\hbar c}(\boldsymbol{n}\cdot\boldsymbol{R}_m)(\boldsymbol{\epsilon}\cdot\boldsymbol{\mu}_m) - i(\boldsymbol{n}\times\boldsymbol{\epsilon})\cdot\boldsymbol{m}_m^{(\mathrm{intr})}\right)\right\} \times D_M(\omega-\omega_{M0})\,. \quad (47)$$

Isotropic circular dichroism The rate constant $k_{0\to M}$ is related to the absorption coefficient $\alpha(\omega)$ by[55] as

$$\alpha(\omega) = \frac{8\pi n_{\mathrm{mol}}}{cE_0^2}\hbar\omega\sum_M k_{0\to M} \approx \alpha^{(0)}(\omega) + \alpha^{(1)}(\omega), \quad (48)$$

where n_{mol} denotes the density of molecular aggregates in a homogeneous sample. The zeroth-order contribution $\alpha^{(0)}(\omega)$ and first-order contribution $\alpha^{(1)}(\omega)$ result from (45) and (47), respectively. The CD signal is defined as the difference between left and right circularly polarized light. Without loss of generality, the light is assumed to propagate in z-direction ($\boldsymbol{n} = (0,0,1)$), resulting in polarization vectors $\boldsymbol{\epsilon}_\mathrm{r} = \frac{1}{\sqrt{2}}(1,-i,0)$ and $\boldsymbol{\epsilon}_\mathrm{l} = \frac{1}{\sqrt{2}}(1,i,0)$. The zeroth-order terms $\alpha^{(0)}_{\mathrm{l/r}}(\omega)$ in (48) are identical because $\boldsymbol{\epsilon}_\mathrm{r} = \boldsymbol{\epsilon}_\mathrm{l}^*$. Therefore, their contribution vanishes for the CD signal. The latter is solely obtained from the first-order contributions by

$$\begin{aligned}\mathrm{CD}(\omega) &= \alpha(\omega)^{(1)}_\mathrm{l} - \alpha(\omega)^{(1)}_\mathrm{r} = \\ &= \frac{8\pi n_{\mathrm{mol}}}{cE_0^2}\hbar\omega\sum_M k^{(1)}_{0\to M,\mathrm{l}} - k^{(1)}_{0\to M,\mathrm{r}} = \\ &= \frac{-4\pi^2 n_{\mathrm{mol}}}{\hbar^2 c}\hbar\omega\sum_M\sum_{m,n} c_m^{(M)} c_n^{(M)}\left(\mathrm{Im}\left\{\frac{E_m E_n}{\hbar^2\omega c}(\boldsymbol{\epsilon}_\mathrm{l}^*\times\boldsymbol{\epsilon}_\mathrm{l})\cdot(\boldsymbol{\mu}_n\times\boldsymbol{\mu}_m)(\boldsymbol{n}\cdot\boldsymbol{R}_m)\right\}+\right. \\ &\left. + \,\mathrm{Re}\left\{\frac{E_n}{\hbar\omega}(\boldsymbol{\epsilon}_\mathrm{r}\cdot\boldsymbol{\mu}_n)(\boldsymbol{n}\times\boldsymbol{\epsilon}_\mathrm{l})\cdot\boldsymbol{m}_m^{(\mathrm{intr})} - \frac{E_n}{\hbar\omega}(\boldsymbol{\epsilon}_\mathrm{l}\cdot\boldsymbol{\mu}_n)(\boldsymbol{n}\times\boldsymbol{\epsilon}_\mathrm{r})\cdot\boldsymbol{m}_m^{(\mathrm{intr})}\right\}\right) \times D_M(\omega-\omega_{M0}), \quad (49)\end{aligned}$$

where again the vector identity (34) has been used. It can be seen from (49), that the CD signal splits into an excitonic contribution and an intrinsic contribution $\mathrm{CD}(\omega) = \mathrm{CD}(\omega)_{(\mathrm{exc})} + \mathrm{CD}(\omega)_{(\mathrm{intr})}$. For an isotropic sample, a rotational average over randomly oriented complexes gives the isotropic signal[48]

$$\mathrm{CD}(\omega)^{(\mathrm{iso})}_{(\mathrm{exc})} = \frac{4\pi^2 n_{\mathrm{mol}}}{3\hbar^2 c}\sum_M\sum_{m,n} c_m^{(M)} c_n^{(M)} \frac{E_m E_n}{2\hbar c}\boldsymbol{R}_{mn}\cdot(\boldsymbol{\mu}_m\times\boldsymbol{\mu}_n)\times D_M(\omega-\omega_{M0}) \quad (50)$$

where $(\boldsymbol{\epsilon}_\mathrm{l}^*\times\boldsymbol{\epsilon}_\mathrm{l}) = i\boldsymbol{n}$, as well as $\sum_{m,n} ... = \frac{1}{2}(\sum_{m,n} ... + \sum_{m,n} ...)$, interchanging of variable names in the second sum, and $\boldsymbol{R}_{mn} = \boldsymbol{R}_m - \boldsymbol{R}_n$ have been used. The orientational average has been carried out using Euler angles[56] as conducted in.[48] Similarly, the intrinsic isotropic contribution reads

$$\mathrm{CD}(\omega)^{(\mathrm{iso})}_{(\mathrm{intr})} = \frac{4\pi^2 n_{\mathrm{mol}}}{3\hbar^2 c}\sum_M\sum_{m,n} c_m^{(M)} c_n^{(M)} 2E_n((-i)\boldsymbol{m}_m^{(\mathrm{intr})}\cdot\boldsymbol{\mu}_n)\times D_M(\omega-\omega_{M0}). \quad (51)$$

Please note that eqs 50 and 51 reduce to expressions derived earlier[48] if the local excitation energies E_m and E_n are approximated by the photon energy $\hbar\omega$. This approximation becomes questionable, when the coupling between transitions of very different excitation energies are included in the theory. We have, therefore, repeated the whole derivation without using this approximation. Note also, that magnetic transition dipole moments are purely imaginary, that is, $(-i)\boldsymbol{m}_m^{(\mathrm{intr})} = \mathrm{Im}\{\boldsymbol{m}_m^{(\mathrm{intr})}\}$. The CD signal can also be obtained using an alternative theory which is commonly used in the literature and known as the Rosenfeld equation.[57,58]

Rosenfeld equation Starting with an underlying interaction Hamiltonian[58]

$$H_{\text{ex}-\text{rad}} = -\boldsymbol{\mu} \cdot \boldsymbol{E} - \boldsymbol{m} \cdot \boldsymbol{B} \tag{52}$$

with $\boldsymbol{E}$ and $\boldsymbol{B}$ denoting the electric and magnetic field, the final expression for the rotational strength of an exciton state $|M\rangle$ is defined as

$$R_M = \text{Im}\{\boldsymbol{\mu}_M \cdot \boldsymbol{m}_M\}. \tag{53}$$

The isotropic CD signal can be obtained from the rotational strength by[42]

$$\text{CD}(\omega) = \frac{8\pi^2 \omega n_{\text{mol}}}{3c\hbar} \sum_M R_M D_M(\omega - \omega_{M0}). \tag{54}$$

Using again the definition of the magnetic transition dipole moment (39) yields for the rotational strength

$$R_M \;=\; Im(\boldsymbol{\mu}_M \cdot \boldsymbol{m}_M) \;=\; Im(\sum_{m,n} c_m^{(M)} c_n^{(M)} \boldsymbol{\mu}_n \cdot \boldsymbol{m}_m) \;=\; \sum_{m,n} c_m^{(M)} c_n^{(M)} (\frac{E_m}{2\hbar c}(\boldsymbol{R}_m \times \boldsymbol{\mu}_m) - i\boldsymbol{m}_m^{(\text{intr})}) \cdot \boldsymbol{\mu}_n \tag{55}$$

which results in a CD signal of

$$\text{CD}(\omega) = \frac{8\pi^2 \omega n_{\text{mol}}}{3c\hbar} \sum_M \sum_{m,n} c_m^{(M)} c_n^{(M)} (\frac{E_m}{2\hbar c}(\boldsymbol{R_m} \times \boldsymbol{\mu}_m) - i\boldsymbol{m}_m^{(\text{intr})}) \cdot \boldsymbol{\mu}_n D_M(\omega - \omega_{M0}). \tag{56}$$

Again, the signal can be split into an excitonic and intrinsic part as

$$\text{CD}(\omega)_{(\text{exc})} = \frac{8\pi^2 \omega n_{\text{mol}}}{3c\hbar} \sum_M \sum_{m,n} c_m^{(M)} c_n^{(M)} \frac{E_m}{2\hbar c} \boldsymbol{\mu}_n \cdot (\boldsymbol{R_m} \times \boldsymbol{\mu}_m) D_M(\omega - \omega_{M0}) \tag{57}$$

and

$$\text{CD}(\omega)_{(\text{intr})} = \frac{8\pi^2 \omega n_{\text{mol}}}{3c\hbar} \sum_M \sum_{m,n} c_m^{(M)} c_n^{(M)} ((-i)\boldsymbol{m}_m^{(\text{intr})} \cdot \boldsymbol{\mu}_n) D_M(\omega - \omega_{M0}). \tag{58}$$

Comparing these expressions with (50) and (51) shows that the only difference lies in the diabatic energy E_n, which has been approximated by the photon energy $\hbar\omega$ in the present formalism. Therefore, the excitonic part in (57) is not origin-independent. Note, that in general only observables which comprise all contributions from a certain order of a multipole extension are origin-independent. However, the definition of the rotational strength in (55) lacks the contribution from the electric transition quadrupole. Fortunately, this argument is not valid regarding the CD signal itself, because it can be shown that also the excitonic contribution of the transition quadrupole vanishes for isotropic samples. This situation leaves the partial approximation of diabatic energies by the photon energy as the reason for the origin-dependent CD signal. The origin-dependence can only be removed by approximating also E_m by the photon energy $\hbar\omega$ as

$$\begin{aligned}\text{CD}(\omega)_{(\text{exc})} = \frac{8\pi^2 \omega n_{\text{mol}}}{3c\hbar} \sum_M \sum_{m,n} c_m^{(M)} c_n^{(M)} \frac{E_m}{2\hbar c} (\boldsymbol{R}_m \times \boldsymbol{\mu}_m) \cdot \boldsymbol{\mu}_n D_M(\omega - \omega_{M0}) \approx \\ \approx \frac{2\pi^2 \omega^2 n_{\text{mol}}}{3c^2\hbar} \sum_M \sum_{m,n} c_m^{(M)} c_n^{(M)} \boldsymbol{R}_{mn} \cdot (\boldsymbol{\mu}_m \times \boldsymbol{\mu}_n) D_M(\omega - \omega_{M0})\end{aligned} \tag{59}$$

where again $\sum_{m,n} ... = \frac{1}{2}(\sum_{m,n} ... + \sum_{m,n} ...)$, and the interchanging of variable names in the second sum have been used. The expression for the CD signal given in (59) is well known from the literature.[48,59]

Overall, there are several levels of theory applicable to computations of CD signals. Whereas (50) together

with (51) represent the most accurate result, approximating the diabatic energies by the photon energy results in well-known expressions for the excitonic part. Although the commonly used Rosenfeld equation, (53) and (54), includes contributions originating from the intrinsic magnetic transition dipole moment, the resulting CD signal is not origin-independent due to the bespoke approximation. Moreover, if also higher energy transitions are taken into account in the calculation of CD signals, the respective energies can be substantially different from the photon energy related to the investigated region of the spectrum. Please note, that in our calculations the high-energy LE states are only included to study their influence on the low-energy (Q_y) region of the spectra. An open question was whether the non-conservativity of the low-energy region of the experimental CD spectrum is mainly caused by the excitonic coupling between high-energy and low-energy excited states of the pigments that redistributed rotational strength, or by the intrinsic (non-excitonic) rotational strength of the special pair. The present calculations show that the latter effect dominates for the present system.

Theory of OD and LD spectra The expressions for the linear absorption (OD) and linear dichroism (LD) spectra read

$$\mathrm{OD}(\omega) \propto \omega \sum_M |\boldsymbol{\mu}_M|^2 D_M(\omega - \omega_{M0}) \tag{60}$$

and

$$\mathrm{LD}(\omega) \propto \omega \sum_M |\boldsymbol{\mu}_M|^2 (1 - 3\cos^2\theta_M) D_M(\omega - \omega_{M0}), \tag{61}$$

respectively, with the electric transition dipole moment of the Mth exciton state

$$\boldsymbol{\mu}_M = \sum_{m,a} c_{ma}^{(M)} \boldsymbol{\mu}_m^{(a)} \tag{62}$$

and the angle θ_M between this transition dipole moment and the membrane normal.

Note that we assume an isotropic sample in the case of the CD and the OD spectrum. In case of LD, the complexes have been oriented in a plane with isotropic orientation with respect to the normal on that plane. This plane was assumed to be parallel to the membrane, in which PSII is embedded.

6.2 CD spectra for alternative parameters and theories

In the following, a comparison of CD spectra calculated for different parameters and models is presented. First, the spectrum of the full model in Fig. 2 of the main text is compared to the spectrum obtained with the same parameters but using the Rosenfeld equation (eqs 57 and 58) for the calculation of the signal (Figure S7, right). Whereas the long-wavelength positive peak obtained in the two theories agrees well, the relative intensity of the two negative peaks is somewhat different. The non-conservativity ratio is very similar (3.6 ± 0.1).

In the left part of Figure S7, the spectra of the full model obtained with the refined site energies (Fig. 2 of the main text) are compared to those obtained with the same model but a site energy set from the literature.[4] The former have been fitted based on a tolerance of 60 cm^{-1} with respect to the latter. Numerical values of these site energy sets are given in Table S9. The CD spectra obtained with the original site energy set contain a slightly narrower main positive band and a somewhat red-shifted main negative band, as compared to the calculations with the refined site energies.

If instead of the 150 cm^{-1} coupling between the Q_y transitions of the special pair pigments the directly calculated value of 193 cm^{-1} (eq 4 of the main text) is used, a larger intensity and a somewhat red-shifted position of the positive peak in the CD spectrum are obtained (Figure S8). Note, that the ratio of non-

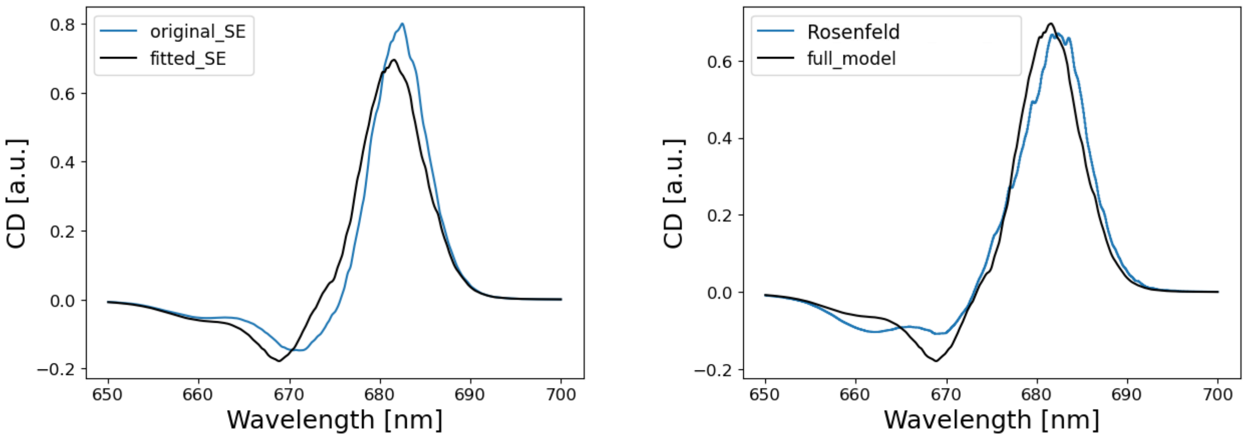

Figure S7: Comparison of spectra calculated in the full model using Q_y energies refined in the present work (same as in Fig. 2 of the main text) with those obtained by using Q_y energies from our earlier work[4] left panel) or with spectra calculated in the Rosenfeld model (right panel), using the refined site energies. Numeric values of site energies are given in Table S9.

conservativity decreases from 3.4 to 3.0 between the calculations for 150 cm^{-1} and 193 cm^{-1}, respectively. Furthermore, the effects of the short-range contributions on the magnitude of the electric transition dipole moment were investigated in Fig. S8. The SR contributions are found to slightly enhance the dipole strengths of the Q_y transition of P_{D1} and P_{D2} by about 4%. However, this enhancement has practically no effect on the CD spectrum. Note that the rotation of the transition dipole moments by the SR effects is taken into account.

The model by Lindorfer *et al.*[1] took into account the coupling between the Q_y and high-energy transitions of the pigments and postulated increased excitonic couplings mediated by CT states in the special pair. Here, we find that this mediation is much weaker than assumed (Tables S6 and S7). In order to further connect to our previous calculations,[1] we replaced the present diabatic transition energies and dipole moments with those obtained for the isolated monomers. Thereby, the effects of higher excited states are included, but short-range effects are omitted, except for the increased special pair coupling, which was kept at 150 cm^{-1}. The ratio of non-conservativity drops from 3.4 to 1.0, with the respective spectra shown in Figure S9. In addition to assuming monomer quantities, the model proposed in ref[1] (i) does not consider intrinsic CD contributions, and (ii) approximates transition energies by the photon energy of the incoming light $\hbar\omega$. Introducing those approximations on top of the considered monomer values yields ratios of non-conservativity of 1.8 (i) and 1.4 (i+ii), respectively. Based on the original model and data of ref,[1] we computed a non-conservativity ratio of 2.0 (without increase of coupling of Q_y states to high-energy LE states via CT states). Remaining discrepancies which could explain the prevailing difference between the non-conservativity ratio of 1.4 and the ratio of 2.0 concern (i) different quantum chemical methods for the computation of monomer excited states, and (ii) fixing the direction of electric transition dipole moments to predefined directions rather than working with quantities obtained from quantum chemistry computations. Note, that the spectra are particularly sensitive to the direction of transition dipole moments (cf. main text).

Finally, we discuss the influence of the reaction field corrections, introduced recently into the Poisson-TrEsp method for the calculation of excitonic couplings.[45] So far, we have used the lower limit $f^{\mathrm{rf}} = 1.0$. We find that for the present system, a larger reaction field factor leads to less agreement between calculated and measured spectra. The CD spectrum obtained for the upper limit $f^{\mathrm{rf}} = 1.44$ are compared in Figure S10 to the experimental spectrum and the spectrum obtained for $f^{\mathrm{rf}} = 1.0$ (same as in Fig. 2, of the main text, full model). The spectra obtained for $f^{\mathrm{rf}} = 1.44$ agree significantly less with the experiment than those

(green).

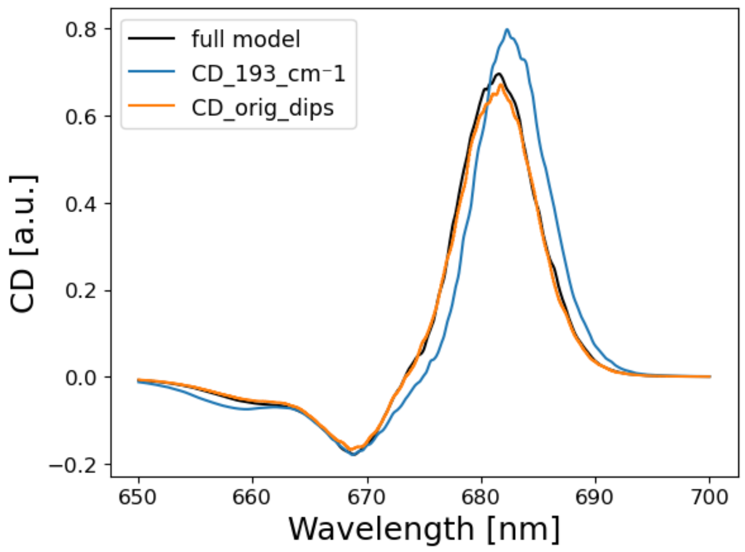

Figure S8: Comparison of full model spectrum (Fig. 2 of the main text) to spectra obtained (i) with altered $Q_y - Q_y$ coupling (150 cm^{-1} to 193 cm^{-1}), and (ii) with monomer electric transition dipole strengths where, in contrast to the full model, short-range effects on the magnitude of dipoles are omitted. Note, that short-range effects regarding the direction of dipoles are still considered.

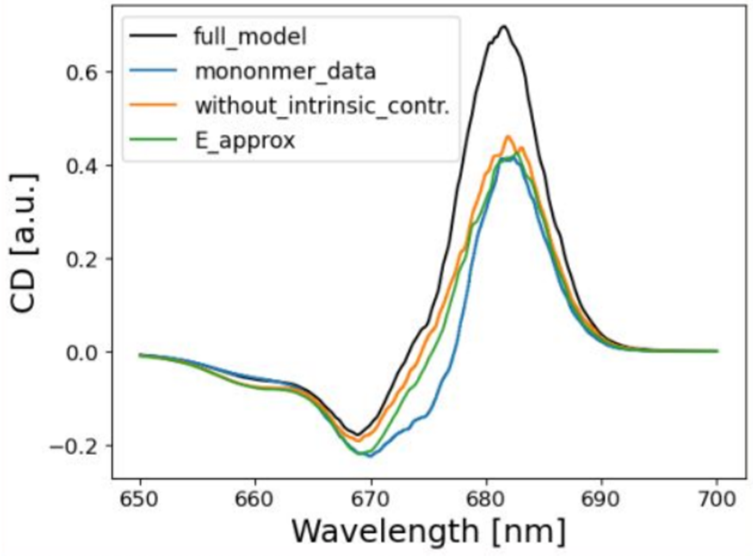

Figure S9: Comparison of final CD-spectrum (black) with (i): energies, couplings, dipoles replaced with respective monomer quantities (blue), (ii): (i) + omitting intrinsic contributions to the CD signal (yellow), (iii): (ii) + approximating transition energies with the energy of the incoming electromagnetic wave ω (green).

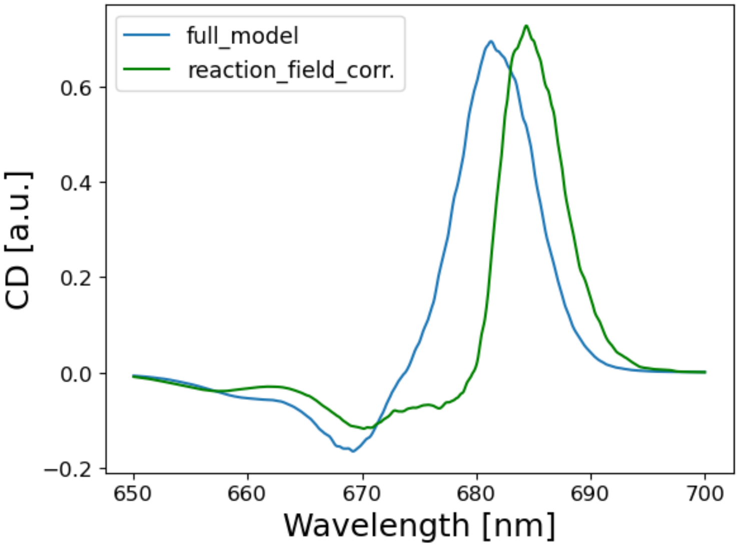

Figure S10: Comparison of final CD-spectrum with additional reaction field corrections as proposed by Friedl et al.;[45] experimental spectrum is from Hall et al.[60]

obtained for $f^{\mathrm{rf}} = 1.0$. Whereas the redshift of the spectrum calculated for $f^{\mathrm{rf}} = 1.44$ could be corrected by a blue-shift of all site energies, there are remaining discrepancies concerning the shape. We note that in a recent application of Poisson-TrEsp to Fenna-Matthews-Olson-protein-reaction center supercomplexes, also a rather low reaction field correction factor $f^{\mathrm{rf}} = 1.15$ has been inferred. A discussion of the uncertainties related to an independent estimation of this factor is given in ref.[45]

7 Construction of a minimal model

In order to extract the essential parts of our full model, which includes 5 LE states per RC pigment, we investigated several approximations. In case of the special pair, these 5 LE states correspond to the 5 lowest diabatic states of $\mathrm{P_{D1}}$ and $\mathrm{P_{D2}}$, where the one with the lowest energy ($\mathrm{Q_y}$) is perturbed by superexchange-type coupling to CT states at higher energies. In a first attempt, we treated all high-energy LE states, that is, all LE states except for the two lowest ($\mathrm{Q_y}$ and $\mathrm{Q_x}$) for the Chls and Pheos and all excited LE states of the Cars in perturbation theory. Similar to the perturbation theory with respect to the coupling to CT states, as provided in the main text and in Section 6.1.1, we obtain perturbed energies

$$E_m^{(a)} = E_{ma}^{(0)} - \sum_{kc}^{\mathrm{he}} \frac{|V_{ma,kc}^{(0)}|^2}{E_{kc}^{(0)} - E_{ma}^{(0)}},$$

where $a = \mathrm{Q_y}$ for all Chls and Pheos, except the special pair, and $a = \mathrm{Q_x}, \mathrm{Q_y}$ for $\mathrm{P_{D1}}$ and $\mathrm{P_{D2}}$. Here, $V_{ma,kc}^{(0)}$ is the excitonic coupling between the ath excited state of site m and the cth high-energy LE state of site k. In the case of the special pair, the energies ($E^{(0)}_{\mathrm{P_{D1}Q_y}}$ and $E^{(0)}_{\mathrm{P_{D2}Q_y}}$), couplings ($V^{(0)}_{\mathrm{P_{D1}}a,\mathrm{P_{D2}}c}$) and transition dipole moments involving the $\mathrm{Q_y}$ states contain already a perturbation by CT states, as described in the main text (eqs 2, 3, and 9 and 11, respectively). Note, that in case of the energies, these effects enter only indirectly since the final energies of LE states of the full model are shifted by a constant obtained from a fit of the experiment, as described in Section 6.1.1.

The excitonic couplings perturbed by the coupling to higher excited LE states are obtained as

$$V_{ma,nb} = V^{(0)}_{ma,nb} - \sum_{kc}^{\text{he}} \frac{V^{(0)}_{ma,kc} V^{(0)}_{kc,nb}}{E^{(0)}_{kc} - E^{(0)}_{ma}} \,, \tag{63}$$

the electric transition dipole moments become

$$\boldsymbol{\mu}^{(a)}_{m} = \boldsymbol{\mu}^{(0)}_{ma} + \sum_{kc}^{\text{he}} \frac{V^{(0)}_{ma,kc}}{E^{(0)}_{ma} - E^{(0)}_{kc}} \boldsymbol{\mu}^{(0)}_{kc} \,, \tag{64}$$

and the magnetic transition dipole moments are

$$\boldsymbol{m}^{(a)}_{m} = \boldsymbol{m}^{(0)}_{ma} + \sum_{kc}^{\text{he}} \frac{V^{(0)}_{ma,kc}}{E^{(0)}_{ma} - E^{(0)}_{kc}} \boldsymbol{m}^{(0)}_{kc} \,. \tag{65}$$

Table S10 contains energies and couplings in the minimal model, the full model, and an effective model, termed effective high-energy (h-e) model, where the couplings to high-energy states are included by the perturbation theory described above. The parameters of the minimal model were taken from our earlier work.[1] For clarity, we assumed here the same transition energies in the full model. Note, however, that in the calculation of optical spectra these values are slightly refined (Table S9). Thus, from a comparison of the three models we can learn the following. The difference between the values of the minimal model and the excerpt of the full model are due to different computation schemes for the long-range couplings. In ref[1] the quantum chemical calculations are less elaborate and do not rely on a refined representative structure of the dimer. Furthermore, the couplings are obtained with TrEsp charges, whereas Mulliken charges have been used for the LR excitonic couplings between the special pair and the remaining RC pigments in our full model. Next, the difference between the excerpt of the full model and the effective model represent the effects of higher LE states that are considered using perturbation theory.

The Q_x-states are kept explicitly in the effective model and are also perturbed by higher LE-states. This inclusion was considered due to the small energy difference between the Q_x- and the Q_y-states. Comparison of the perturbed excitonic couplings $V_{ma,nb}$ and energies $E^{(a)}_{m}$ of the effective h-e model with the unperturbed ones $V^{(0)}_{ma,nb}$ and $E^{(0)}_{ma}$, respectively, of our full model shows that the differences are small (2nd and 3rd numbers in Table S10). Moreover, the couplings are also very similar to the ones of our minimal model (1st numbers in Table S10) that are taken from ref.[1] Note that the non-special pair excitonic couplings of our full model were also taken from this ref. The differences in the couplings between the special pair and the remaining pigments in the present full model (obtained with Mulliken charges) are very close to those obtained in ref[1] (with TrEsp charges).

The contributions to the CD spectrum from the Q_x states of the special pair are investigated in Fig. S11, where we compare spectra calculated in the minimal model with and without including the Q_x states of the special pair. There are practically no differences and, hence, the Q_x states of the special pair can be omitted.

Next, we studied the static disorder of the Q_y states of the special pair. So far, this disorder has been described microscopically based on MD snapshots, whereas the static disorder of the remaining RC pigments was taken randomly from a Gaussian distribution function of 150 cm^{-1} FWHM. It turns out that practically the same inhomogeneous optical spectra are obtained by taking also the Q_y transition energies of the special pair from a Gaussian distribution function, but with an increased width of 280 cm^{-1}, which takes into account additional broadening due to the involvement of CT state couplings (Fig. S12).

On the grounds described above, we find the following approximations valid: (i) using crystal structure

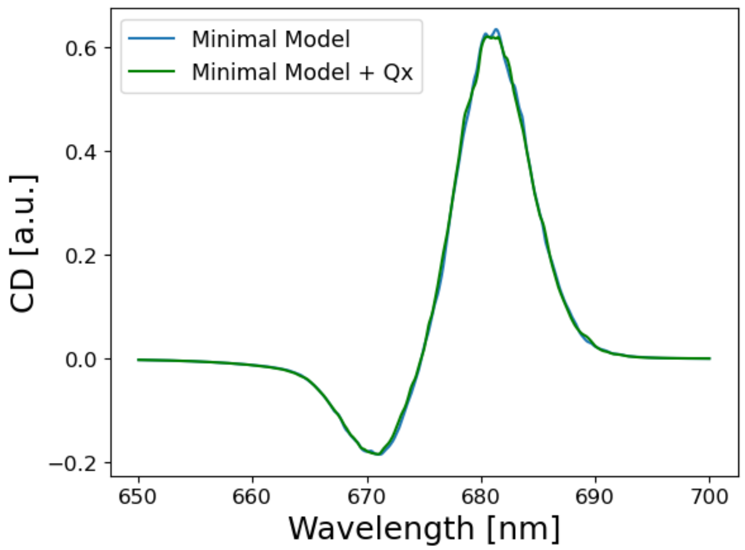

Figure S11: CD spectra obtained in the minimal model with (green line) and without (blue line) including the Q_x transition of the P_{D1} and P_{D2} pigments.

coordinates, (ii) using long-range coupling values except for the intra special pair coupling, (iii) omitting the Q_x-states, and (iv) omitting the effects of higher LE-states. All these approximations result in a negligible change of the spectra and ratios of non-conservativity. The latter are smaller than 0.1 and, therefore, below the tolerance limit. Using the unfitted site energies as originally proposed in ref[4] even leads to an increase of the non-conservativity from 3.1 to 3.3 compared to the use of the site energies from the full model calculations (Table S9). Therefore, we take the originally proposed energies in our minimal model. Omitting the influence of CT-states but still accounting for electron-electron exchange, results in a reduction of the non-conservativity ratio from 3.3 to 2.8, whereas the use of transition dipoles from monomer calculations, i.e., neglecting all short-range contributions, leads to a ratio of 0.9 as described in the main text.

In Figure S13, the OD, LD, and CD spectra are shown for both sets of site energies using the full model and in Figure S14 these spectra are shown again for the two sets of site energies, but using the minimal model, derived above.

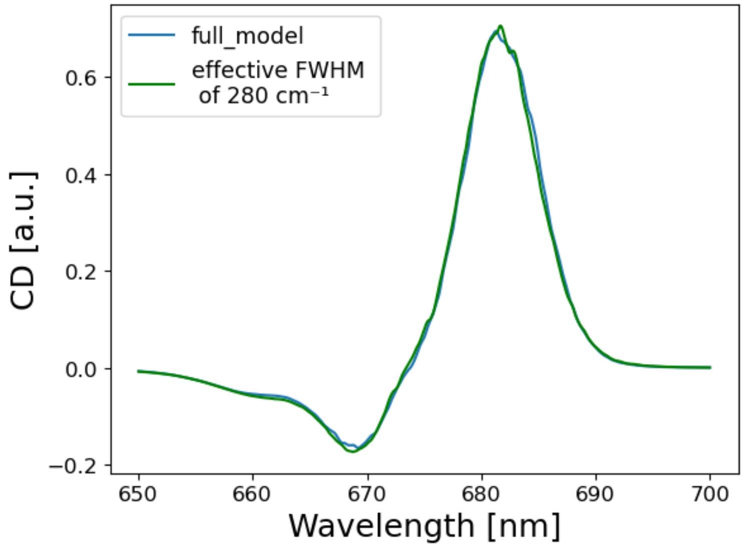

Figure S12: CD spectra obtained in the full model, using individual Hamiltonian parameter fluctuations (blue) for the special pair and effective site energy fluctuations of the Q_y energies of P_{D1} and P_{D2} (green).

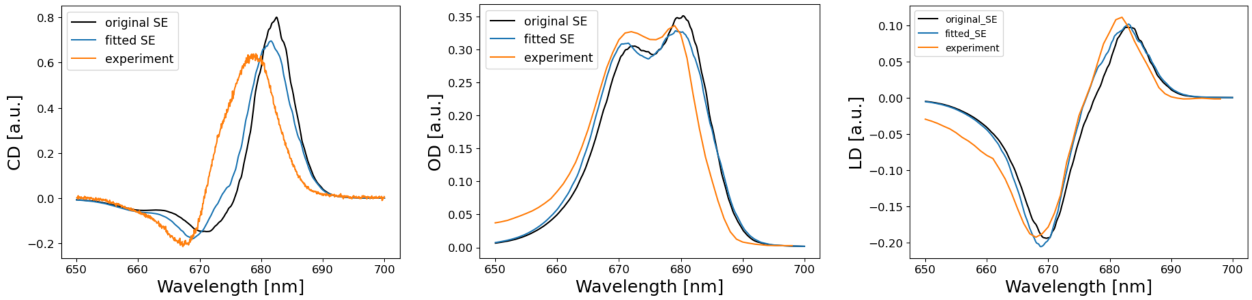

Figure S13: CD (left), OD (middle) and LD (right) spectra obtained with original site energies from[4] and fitted site energies for the full model calculations compared to experimental spectra from[61]

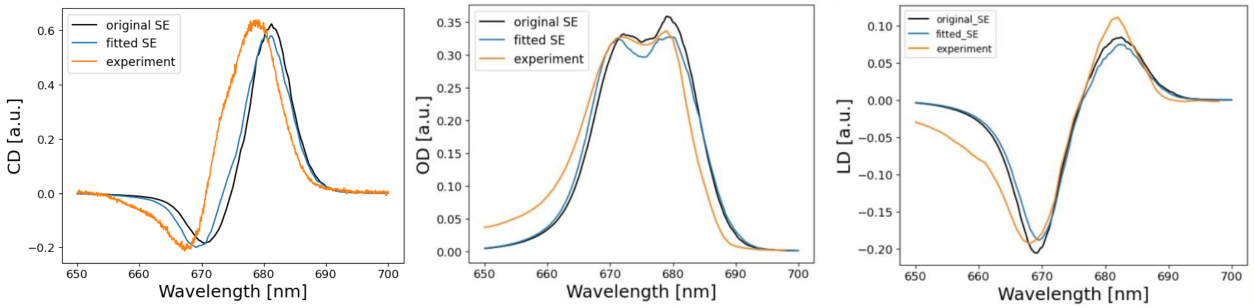

Figure S14: CD (left), OD (middle) and LD (right) spectra obtained with original site energies from[4] and fitted site energies for the minimal model calculations compared to experimental spectra from[61]

Table S10: Hamiltonian parameters for minimal model, excerpt of full reaction center Hamiltonian as well as implicit high-energy (h-e) model (*minimal/full/implicit h-e*); note that the Q_x-states are not included in the minimal model. The original site energies (Table S9) are taken here in the minimal and full model and are used as the umperturbed energies $E_{ma}^{(0)}$ in the h-e effective model (eq 63). The excitonic coupling 150 cm^{-1} between Q_y transitions of P_{D1} and P_{D2} in the minimal and full model has been adopted from the directly calculated value (eq 4 of the main text) to fit the experimental CD spectrum (see also Fig. S8).

	$P_{D1}-Q_y$	$P_{D1}-Q_x$	$P_{D2}-Q_y$	$P_{D2}-Q_x$	Chl_{D1}	Chl_{D2}	$Pheo_{D1}$	$Pheo_{D2}$	$Chlz_{D1}$	$Chlz_{D2}$
$P_{D1}-Q_y$	14970.1/<br>14970.1/<br>14937.3	-/<br>0.0/<br>-7.35	150.0/<br>150.0/<br>150.0	-/<br>115.0/<br>114.1	-35.2/<br>-28.3/<br>-28.7	-44.7/<br>-41.9/<br>-42.7	-3.0/<br>-3.9/<br>-4.4	11.5/<br>10.4/<br>9.5	0.4/<br>0.6/<br>0.5	0.6/<br>0.6/<br>0.5
$P_{D1}-Q_x$	-	-/<br>18934.0/<br>18917.7	-/<br>39.5/<br>40.5	-/<br>10.5/<br>9.6	-/<br>-18.6/<br>-18.2	-/<br>-22.2/<br>-22.2	-/<br>-4.9/<br>-5.1	-/<br>4.0/<br>3.4	-/<br>-0.6/<br>-0.6	-/<br>-0.2/<br>-0.2
$P_{D2}-Q_y$		-	14970.1/<br>14970.1/<br>14948.5	-/<br>0.0/<br>4.5	-43.8/<br>-45.5/<br>-45.9	-28.7/<br>-26.5/<br>-25.5	13.0/<br>11.7/<br>12.9	-2.3/<br>-3.3/<br>-2.2	0.6/<br>0.5/<br>0.5	0.6/<br>0.4/<br>0.4
$P_{D2}-Q_x$	-	-	-	-/<br>19215.5/<br>19207.0	-/<br>25.8/<br>24.9	-/<br>12.2/<br>10.9	-/<br>-3.8/<br>-5.4	-/<br>3.5/<br>2.6	-/<br>0.2/<br>0.1	-/<br>0.7/<br>0.7
Chl_{D1}		-		-	14749.0/<br>14949.0/<br>14742.0	4.0/<br>4.0/<br>2.5	46.0/<br>46.0/<br>46.2	-2.3/<br>-2.3/<br>-2.5	1.5/<br>1.5/<br>1.5	-0.1/<br>-0.1/<br>-0.1
Chl_{D2}		-		-		14925.0/<br>14925.0/<br>14919.7	-2.5/<br>-2.5/<br>-2.7	44.7/<br>44.7/<br>44.8	0.0/<br>0.0/<br>-0.1	1.7/<br>1.7/<br>1.8
$Pheo_{D1}$		-		-			14925.0/<br>14925.0/<br>14919.7	1.6/<br>1.6/<br>1.8	-2.4/<br>-2.4/<br>-2.3	-0.2/<br>-0.1/<br>-0.1
$Pheo_{D2}$		-		-				14815.0/<br>14815.0/<br>14813.6	-0.2/<br>-0.2/<br>-0.1	-2.4/<br>-2.4/<br>-2.5
$Chlz_{D1}$		-		-					14975.1/<br>14975.1/<br>14972.2	0.1/<br>0.1/<br>0.1
$Chlz_{D2}$		-		-						15038.6/<br>15038.6/<br>15037.2

8 Dipole moment analysis

Full model: Tables S11 and S12 provide further results regarding the differences between the electric and intrinsic magnetic transition dipole moment $\boldsymbol{\mu}_{ma}^{\mathrm{mon}}$ and $\boldsymbol{m}_{ma}^{\mathrm{mon}}$, respectively, of the isolated special pair pigments $m = \mathrm{P_{D1}}, \mathrm{P_{D2}}$ and the corresponding transition dipole moments $\boldsymbol{\mu}_m^{(a)}$ (eqs 8 and 9 of the main text) and $\boldsymbol{m}_m^{(a)}$ (eqs 10 and 11 of the main text), obtained by the diabatization from the dimer calculations for the first 5 excited LE states ($a = 1, ..., 5$), and including a perturbation by CT states for the first excited state ($a = 1$). Unfortunately, TDDFT calculations do not allow for an assignment of higher excited states to the $\mathrm{B_x}-, \mathrm{B_y}-$, and $\mathrm{N_{x+xy}}$-states of the isolated pigments. However, comparing the electric transition dipole moments obtained from diabatization with those from monomer calculations allows at least an assignment of the excited states resulting from those two approaches as shown in Table S11 and further illustrated in Figure S15. Note that such an assignment is unproblematic for the $\mathrm{P_{D1}}$ pigment, whereas for the fourth and fifth excited state of $\mathrm{P_{D2}}$ it is more ambiguous, despite the high quality of the diabatization. A description of the quantification of this quality is given in Section 5.1.

According to Tables S11 and S12 as well as Figures S15 and S16, the magnetic transition dipole moments $\boldsymbol{m}_m^{(a)}$ show higher differences than the electric transition dipole moments $\boldsymbol{\mu}_m^{(a)}$ from their respective monomer values $\boldsymbol{m}_{ma}^{\mathrm{mon}}$ and $\boldsymbol{\mu}_{ma}^{\mathrm{mon}}$. Note, that the magnetic transition dipole moments for the first excited state ($a = 1$) are very similar in both cases ($\mathrm{P_{D1}}$ and $\mathrm{P_{D2}}$). As the diabatization procedure yields well-localized states (cf. Table S11, third column), we explain the deviations for higher excited states with the larger spatial extent of their wave functions, which increases the non-CT-SR effects. From Table S12 one can identify the $\boldsymbol{m}_m^{(a)}$ of the third excited state ($a = 3$) as the one with the highest deviation from the monomer calculations. As illustrated in Figure S16, $\boldsymbol{m}_{\mathrm{P_{D1}}3}^{\mathrm{mon}}$ and $\boldsymbol{m}_{\mathrm{P_{D1}}}^{(3)}$ have a significant component in the pigment plane, in contrast to the other states ($a \neq 3$).

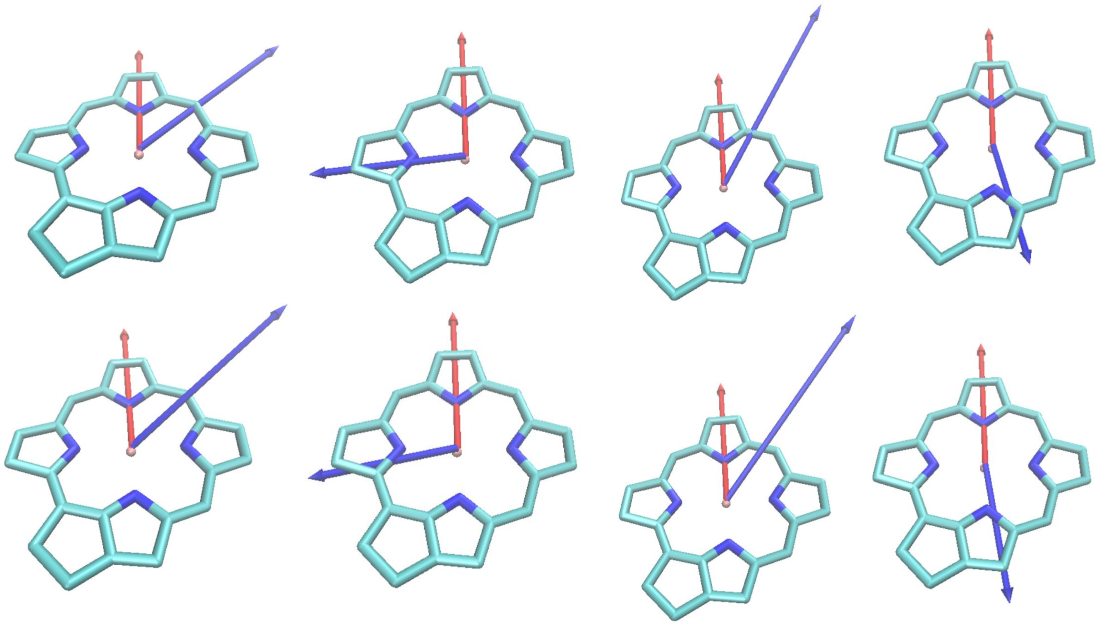

Figure S15: Electric transition dipole moments of 2.-5. LE-state of P_{D1} (blue) relative to the reference vector from the pigment center to the N_B-atom (red); **top:** $\boldsymbol{\mu}_{ma}^{\mathrm{mon}}$ from monomer calculations, **bottom:** $\boldsymbol{\mu}_{m}^{(a)}$ from dimer calculations after diabatization.

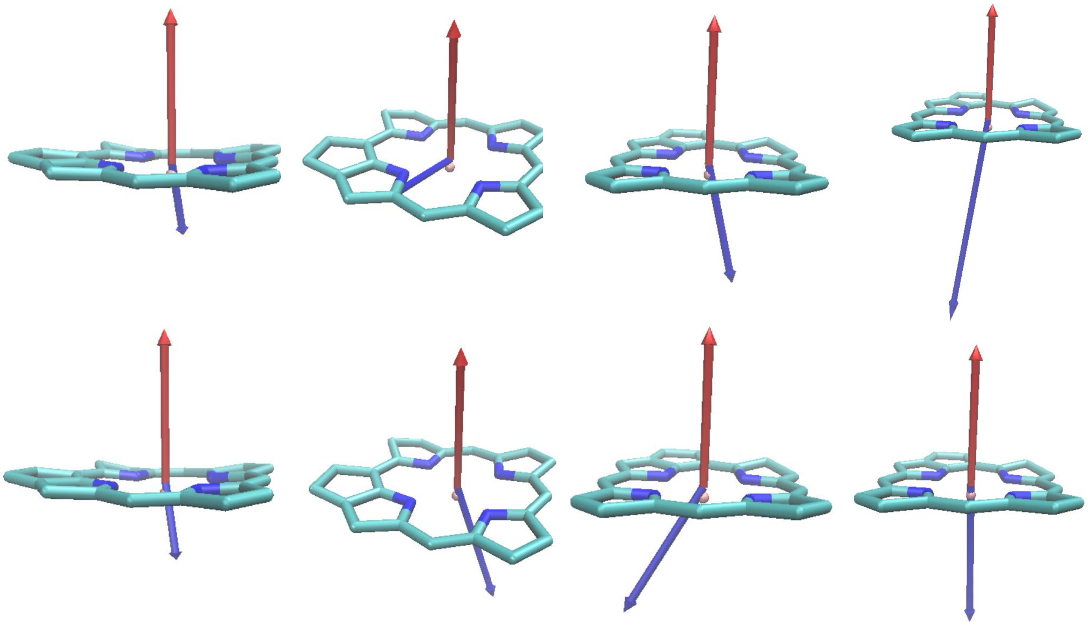

Figure S16: Intrinsic magnetic transition dipole moments of 2.-5. LE-state of P_{D1} (blue) relative to the reference pigment plane normal vector (red); **top:** $\boldsymbol{m}_{ma}^{\mathrm{mon}}$ from monomer calculations, **bottom:** $\boldsymbol{m}_{m}^{(a)}$ from dimer calculations after diabatization.

Table S11: Electric transition dipole moments of the five lowest excited states ($a = 1 \ldots 5$) of the special pair pigments ($m = \mathrm{P_{D1}}, \mathrm{P_{D2}}$) obtained by calculations on isolated monomers ($\boldsymbol{\mu}_{ma}^{\mathrm{mon}}$) or dimer calculations and diabatization ($\boldsymbol{\mu}_{m}^{(\mathrm{a})}$, eq 8 of the main text). For the $\mathrm{Q_y}$ states ($a = 1$), we also included the perturbation by CT states (numbers in brackets, eq 9 of the main text). Angles ϕ and θ are defined in Fig. 4 of the main text. The quality of LE states after diabatization is defined in section. 5.1.

Pigment m	LE state a	Quality [%]	$\lvert\boldsymbol{\mu}\rvert$ [A. U.] $\boldsymbol{\mu}_{ma}^{\mathrm{mon}}$ / $\boldsymbol{\mu}_{m}^{(\mathrm{a})}$	θ [deg] $\boldsymbol{\mu}_{ma}^{\mathrm{mon}}$ / $\boldsymbol{\mu}_{m}^{(\mathrm{a})}$	ϕ [deg] $\boldsymbol{\mu}_{ma}^{\mathrm{mon}}$ / $\boldsymbol{\mu}_{m}^{(\mathrm{a})}$
$\mathrm{P_{D1}}$	1	99	2.14/2.10 (2.14)	90.1/86.2 (85.5)	1.0/2.7 (3.1)
	2	99	1.03/1.04	90.5/96.1	48.4/48.2
	3	98	3.19/2.83	91.2/89.2	81.2/78.0
	4	97	2.63/2.40	93.3/81.9	23.3/36.9
	5	98	2.42/2.83	89.7/95.8	15.5 /8.0
$\mathrm{P_{D2}}$	1	99	2.05/2.07 (2.10)	89.9/93.3 (93.8)	0.6/6.1 (6.4)
	2	99	0.81/0.83	87.6/85.5	47.5/53.1
	3	98	3.18/2.79	92.5/90.5	81.2/79.9
	4	98	3.21/2.37	91.2/94.5	11.1/38.7
	5	98	1.60/2.84	88.3/95.8	42.4/6.4

Table S12: Same as in Table S11 but for magnetic transition dipole moments $\boldsymbol{m}_{ma}^{\mathrm{mon}}$ (isolated monomers) and $\boldsymbol{m}_{m}^{(\mathrm{a})}$ (eqs 10 and 11 of the main text).

Pigment m	LE state a	$\lvert\boldsymbol{m}\rvert$ [A. U.] $\boldsymbol{m}_{ma}^{\mathrm{mon}}$ / $\boldsymbol{m}_{m}^{(\mathrm{a})}$	θ [deg] $\boldsymbol{m}_{ma}^{\mathrm{mon}}$ / $\boldsymbol{m}_{m}^{(a)}$	ϕ [deg] $\boldsymbol{m}_{ma}^{\mathrm{mon}}$ / $\boldsymbol{m}_{m}^{(a)}$
$\mathrm{P_{D1}}$	1	1.10/1.03 (1.02)	2.2/3.6 (4.0)	71.0/79.7 (89.9)
	2	0.20/0.16	12.6/19.7	18.6/76.5
	3	0.20/0.65	79.4 /17.5	14.5/30.7
	4	0.26/0.34	17.9/44.8	39.7/59.9
	5	0.69/0.23	14.8/27.7	77.0/72.2
$\mathrm{P_{D2}}$	1	1.09/1.03 (1.03)	1.3/4.0 (4.0)	71.2/88.0 (87.8)
	2	0.15/0.10	18.6/16.5	24.4/85.9
	3	0.22/0.62	84.2/16.3	12.3/31.4
	4	0.20/0.39	72.8/23.4	50.8/85.9
	5	0.71/0.17	10.8/59.4	20.4/62.1

9 Embedding special pair adiabatic states into the reaction center Hamiltonian

As an alternative, the 30 adiabatic states from the excited states calculations have been embedded in the reaction center Hamiltonian, i.e., the special pair dimer has been treated as a single pigment in this type of computations. The couplings to other pigments have been calculated for the adiabatic transition densities using the Poisson-TrEsp method. The CD-signals have been calculated based on the Rosenfeld equation (eq (56)). The post-processing of parameters is less straightforward compared to the diabatic case outlined above. A conceptional problem is that it is more difficult to rescale the transition dipole moments, based on experimental values of the isolated monomers. The increased quantum chemical transition dipoles cause a too large excitonic coupling and, thereby, a too large splitting between adiabatic states, besides a too large transition dipole strength of the adiabatic states. For the optical spectra in the Q_y region, only the first two adiabatic excited states of the special pair are relevant. Therefore, we shifted the energies of these states to the values we obtained by the diabatization before. Since the dipole strength of these two adiabatic states depends mainly on that of the first LE state of P_{D1} and P_{D2}, we rescaled the dipole strength of these adiabatic states by a factor 0.86, as determined above for the two LE states. Accordingly, the factor 0.91 for the higher excited LE states (see above) is applied to rescale all higher excited state transition dipole moments.

The fluctuations of the Hamiltonian parameters to account for static disorder have been calculated by analyzing the variations of the 20 adiabatic states given by the provided snapshots. The remaining 10 highest excited states have been assumed to vary according to the mean fluctuation of the 20 lower excited states.

The resulting ratio of non-conservativity for treating the special pair as a supermolecule is 3.6±0.1.

10 Assessment of possible errors

Assessment of possible errors of quantum chemical calculations In order to investigate how the principal result of the present work, namely the rotation of electric transition dipole moments by non-CT-SR effects in the special pair, depends on possible errors of quantum chemistry, we have performed additional calculations. These calculations were done at the ωB97X-D3(BJ)/Def2-SVP level of theory, using the ORCA program. The results are shown in Table S13. First, we performed the calculations in the gas phase, that is, without taking the protein environment into account. As in the calculations with electrostatic embedding into the protein, we obtain a 4.4° out-of-plane rotation of the diabatic electric transition dipole moment of P_{D1} and also the rotation angles θ for the P_{D2} monomer is very similar in the gas phase (3.6 °) and in the protein environment (3.9 °). An illustration of these results is given in Fig. S17.

Next, in order to investigate a possible basis set superposition error (BSSE), we performed monomer calculations, in which the basis set functions of the other monomer in the special pair (ghost orbitals) were included. Looking at the results of this calculation ("Gas Phase with Ghost Orb." in Table S13), we find that introducing the ghost orbitals has a very small effect on the transition dipole moment orientations. The maximum out-of-plane rotation of the electric transition dipole moment (difference in angle θ) with and without the ghost orbitals is 0.6°, obtained for P_{D2}. Hence, the BSSE is not causing the important 4° out-of-plane rotation of electric transition dipole moments of P_{D1} and P_{D2} obtained by the diabatisation.

Finally, to separate the contributions of the Coulombic and non-CT-SR effects of the dimer, we performed a gas phase dimer calculation. However, this time only one monomer was given a full QM treatment while the second was described by atomic partial charges. We named this calculation "Gas Phase with Point Charges" in Table S13. The presence of the second monomer as point charges had practically no effect on the transition dipole moments. Thus, we conclude that the tilt of diabatic electric transition dipole moments

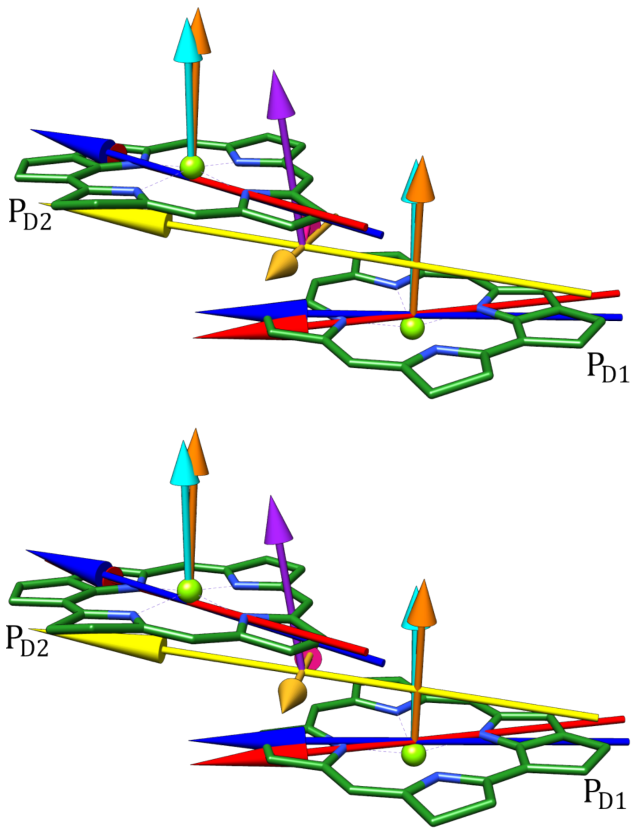

Figure S17: **Upper part:** Gas-phase calculated Q_y monomeric electric (red) and magnetic (orange) transition dipole moments are compared with the respective transition dipole moments resulting from dimer calculations and subsequent diabatization and perturbation by CT states (eqs 9 and 11, respectively, of the main text) shown in blue and cyan, respectively. The adiabatic electric transition dipole moments for the $M = 1$ (yellow) and $M = 2$ (gold) states are shown in yellow and gold, respectively, and the respective adiabatic magnetic transition dipole moments are shown in purple and pink. **Lower part:** Same as upper part, but including the protein environment as point charges (electrostatic embedding).

originates from intra-dimer non-CT-SR effects, which are only present in the full dimer calculation. BSSE and polarization effects by the protein environment have only very minor effects on the transition dipole moments. To further demonstrate this effect, we performed a geometry scan. Starting with our representative structure, we increased the distance between the two pigments by moving the P_{D2} pigment along the normal on the plane of the P_{D1} pigment. In this way, we maintain the pigment's relative orientation while increasing their distance. The scan results (Fig. S18) clearly show that as the inter-pigment distance is increased, the diabatic transition dipole moments are rotating toward their orientations obtained for the isolated monomers (Fig. S18 A and B, and Table S13). In Fig. S18 C, the magnitude of the two lowest ($M = 1, 2$) adiabatic transition dipole moments are shown as a function of distance increase Δr. For $\Delta r = 0$ (native distance), the transition dipole moment of the lowest adiabatic state ($M = 1$) is larger than that of $M = 2$, reflecting the "in-line" type geometry of transition dipole moments. At Δr around 5 Å, the magnitudes of the adiabatic transition dipole moments become equal to that of the adiabatic transition dipole moments. In this case, the excitonic coupling becomes zero. For larger separations Δr, the dipole geometry changes towards "sandwich"-type and the higher-energy adiabatic state ($M = 2$) gets the largest dipole moment magnitude. With increasing distance, the excitonic coupling decreases and the dipole strengths of adiabatic states slowly converge towards that of the diabatic states.

Table S13: Q_y electric and intrinsic magnetic transition dipole moments of the special pair pigments, with ("Protein") and without ("Gas Phase") the protein environment. Angles Θ and Φ are defined in Fig. 4 of the main text. The values for the protein environment are identical to those of Table 2 of the main text. In "Gas Phase with Ghost Orb." and in "Gas Phase with Point Charges" the BSSE and the Coulomb coupling, respectively, were investigated (see text).

m	P_{D1}		P_{D2}	
	Protein			
$\boldsymbol{\mu}_m^{Q_y}$	Θ[°][3]	Φ[°][3]	Θ[°][3]	Φ[°][3]
mon.	90.2	1.0	89.9	0.6
diab.[1]	85.6	3.1	93.9	6.4
$\boldsymbol{m}_m^{Q_y}$	Θ[°]	Φ[°]	Θ[°]	Φ[°]
mon.	2.2	71.0	1.3	71.2
diab.[2]	4.0	89.9	4.0	87.8
	Gas Phase			
$\boldsymbol{\mu}_m^{Q_y}$	Θ[°]	Φ[°]	Θ[°]	Φ[°]
mon.	90.4	0.7	89.6	2.1
diab.[1]	85.7	4.5	93.6	7.7
$\boldsymbol{m}_m^{Q_y}$	Θ[°]	Φ[°]	Θ[°]	Φ[°]
mon.	2.2	73.6	1.2	76.3
diab.[2]	3.5	85.0	3.7	89.8
	Gas Phase with Ghost Orb.			
$\boldsymbol{\mu}_m^{Q_y}$	Θ[°]	Φ[°]	Θ[°]	Φ[°]
mon.	90.0	0.7	90.2	1.7
$\boldsymbol{m}_m^{Q_y}$	Θ[°]	Φ[°]	Θ[°]	Φ[°]
mon.	2.0	70.5	1.2	72.1
	Gas Phase with Point Charges			
$\boldsymbol{\mu}_m^{Q_y}$	Θ[°]	Φ[°]	Θ[°]	Φ[°]
mon.	90.4	1.8	89.5	3.8
$\boldsymbol{m}_m^{Q_y}$	Θ[°]	Φ[°]	Θ[°]	Φ[°]
mon.	2.1	75.1	1.2	82.8

[1] μ_{mQ_y} as defined in Eq. 9 of the main text

[2] m_{mQ_y} as defined in Eq. 11 of the main text

[3] defined in Fig. 4 in the main text.

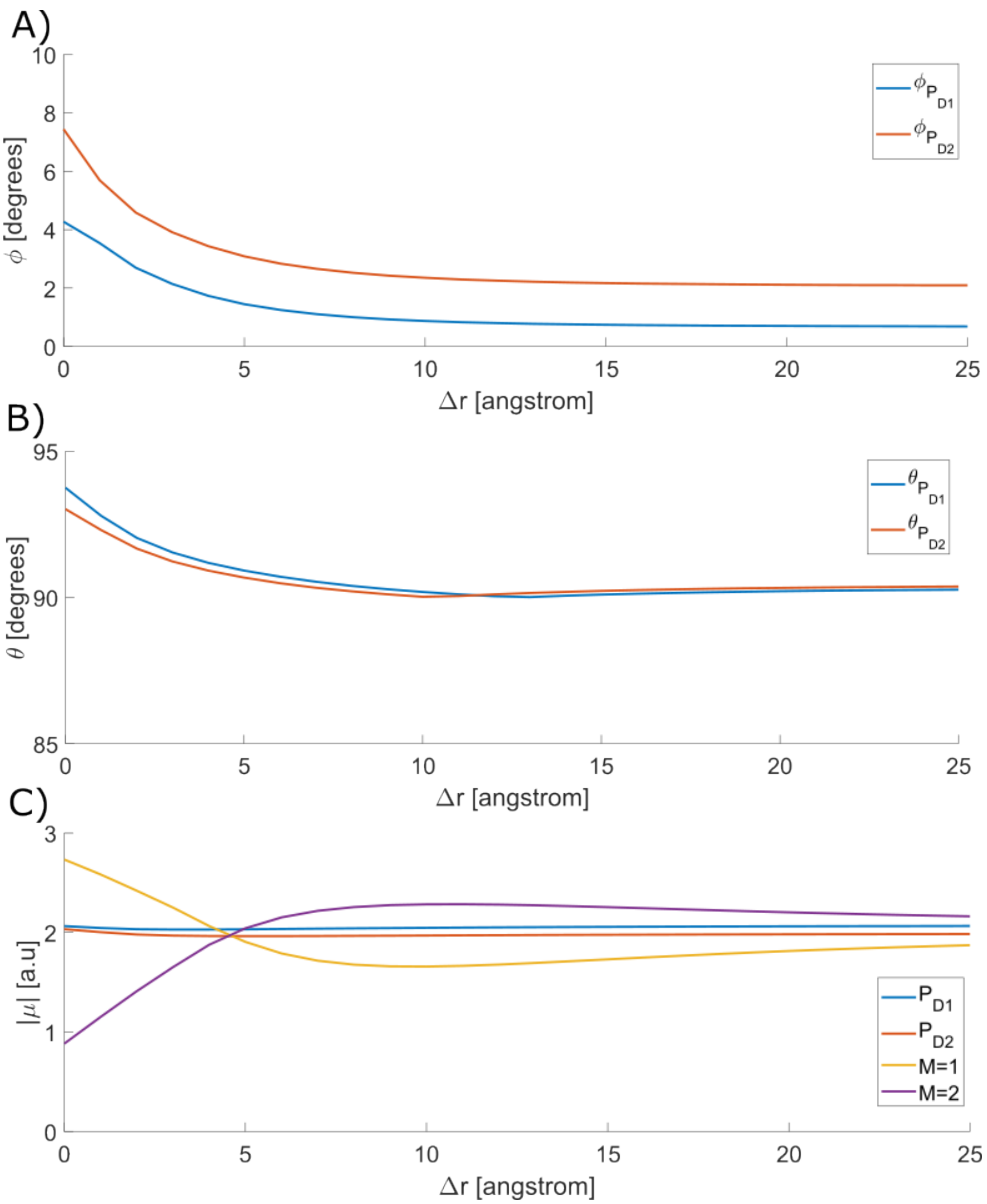

Figure S18: Orientation and magnitude of electric transition dipole moments of the special pair, as a function of distance increase Δr. Starting with the native structure ($\Delta r = 0$), the distance was increased by moving the P_{D2} pigment along the normal on the plane of the P_{D1} pigment. A) Angle Φ of the diabatic electric transition dipole moments of P_{D1} and P_{D2}. B) Angle Θ of the diabatic electric transition dipole moments of P_{D1} and P_{D2}. (For a definition of angles see Fig. 4 of the main text. C) The magnitudes of electric transition dipole moments of the adiabatic states $M = 1, 2$ are compared to that of the diabatic states of P_{D1} and P_{D2}.

References

[1] Lindorfer, D.; Müh, F.; Purchase, R.; Krausz, E.; Renger, T. Non-conservative circular dichroism of photosystem II reaction centers: Is there an enhancement by a coupling with charge transfer states? *J. Photochem. Photobiol.* **2021**, *404*, 112883.

[2] Umena, Y.; Kawakami, K.; Shen, J.-R.; Kamiya, N. Crystal structure of oxygen-evolving photosystem II at a resolution of 1.9 Å. *Nature* **2011**, *473*, 55–60.

[3] Renger, T.; Müh, F. Understanding photosynthetic light-harvesting: a bottom up theoretical approach. *Phys. Chem. Chem. Phys.* **2013**, *116*, 367–388.

[4] Müh, F.; Plöckinger, M.; Renger, T. Electrostatic asymmetry in the reaction center of photosystem II. *J. Phys. Chem. Lett.* **2017**, *8*, 850–858.

[5] Rabenstein, B.; Knapp, E.-W. Calculated pH-dependent population and protonation of carbon-monoxy-myoglobin conformers. *Biophys. J.* **2001**, *80*, 1141–1150.

[6] Kieseritzky, G.; Knapp, E.-W. Optimizing pKa computation in proteins with pH adapted conformations. *Proteins: Struct., Funct., Bioinf.* **2008**, *71*, 1335–1348.

[7] Rabenstein, B. Monte Carlo methods for simulation of protein folding and titration. Ph.D. thesis, 2000.

[8] Narzi, D.; Guidoni, L. Structural and dynamic insights into Mn_4Ca cluster-depleted photosystem II. *Phys. Chem. Chem. Phys.* **2021**, *23*, 27428–27436.

[9] Han, R.; Rempfer, K.; Zhang, M.; Dobbek, H.; Zouni, A.; Dau, H.; Luber, S. Investigating the structure and dynamics of apo-photosystem II. *ChemCatChem* **2019**, *11*, 4072–4080.

[10] Jo, S.; Kim, T.; Iyer, V. G.; Im, W. CHARMM-GUI: A web-based graphical user interface for CHARMM. *J. Comput. Chem.* **2008**, *29*, 1859–1865.

[11] Maier, J. A.; Martinez, C.; Kasavajhala, K.; Wickstrom, L.; Hauser, K. E.; Simmerling, C. ff14SB: Improving the accuracy of protein side chain and backbone parameters from ff99SB. *J. Chem. Theory Comput.* **2015**, *11*, 3696–3713.

[12] Jorgensen, W. L.; Chandrasekhar, J.; Madura, J. D.; Impey, R. W.; Klein, M. L. Comparison of simple potential functions for simulating liquid water. *J. Chem. Phys.* **1983**, *79*, 926–935.

[13] Seminario, J. M. Calculation of intramolecular force fields from second-derivative tensors. *Int. J. Quantum Chem.* **1996**, *60*, 1271–1277.

[14] Li, P.; Merz, K. M. J. MCPB.py: A Python based metal center parameter builder. *J. Chem. Inf. Model.* **2016**, *56*, 599–604.

[15] Case, D. et al. Amber 2021. 2021; University of California, San Francisco.

[16] Becke, A. D. Density-functional thermochemistry. I. The effect of the exchange-only gradient correction. *J. Chem. Phys.* **1992**, *96*, 2155–2160.

[17] Rassolov, V. A.; Pople, J. A.; Ratner, M. A.; Windus, T. L. 6-31G* basis set for atoms K through Zn. *J. Chem. Phys.* **1998**, *109*, 1223–1229.

[18] Frisch, M. J. et al. Gaussian~16 Revision C.01. 2016; Gaussian Inc. Wallingford CT.

[19] Neese, F.; Wennmohs, F.; Becker, U.; Riplinger, C. The ORCA quantum chemistry program package. *J. Chem. Phys.* **2020**, *152*, 224108.

[20] Bannwarth, C.; Ehlert, S.; Grimme, S. GFN2-xTB—An accurate and broadly parametrized self-consistent tight-binding quantum chemical method with multipole electrostatics and density-dependent dispersion contributions. *J. Chem. Theory Comput.* **2019**, *15*, 1652–1671.

[21] Weigend, F.; Ahlrichs, R. Balanced basis sets of split valence, triple zeta valence and quadruple zeta valence quality for H to Rn: Design and assessment of accuracy. *Phys. Chem. Chem. Phys.* **2005**, *7*, 3297–3305.

[22] Grimme, S.; Ehrlich, S.; Goerigk, L. Effect of the damping function in dispersion corrected density functional theory. *J. Comput. Chem.* **2011**, *32*, 1456–1465.

[23] Izsák, R.; Neese, F. An overlap fitted chain of spheres exchange method. *J. Chem. Phys.* **2011**, *135*, 144105.

[24] Lin, Y.-S.; Li, G.-D.; Mao, S.-P.; Chai, J.-D. Long-range corrected hybrid density functionals with improved dispersion corrections. *J. Chem. Theory Comput.* **2013**, *9*, 263–272.

[25] Isborn, C. M.; Götz, A. W.; Clark, M. A.; Walker, R. C.; Martínez, T. J. Electronic absorption spectra from MM and ab Initio QM/MM molecular dynamics: Environmental effects on the absorption spectrum of photoactive yellow protein. *J. Chem. Theory Comput.* **2012**, *8*, 5092–5106.

[26] Ufimtsev, I. S.; Martinez, T. J. Quantum chemistry on graphical processing units. 3. Analytical energy gradients, geometry optimization, and first principles molecular dynamics. *J. Chem. Theory Comput.* **2009**, *5*, 2619–2628.

[27] Titov, A. V.; Ufimtsev, I. S.; Luehr, N.; Martinez, T. J. Generating efficient quantum chemistry codes for novel architectures. *J. Chem. Theory Comput.* **2013**, *9*, 213–221.

[28] Najibi, A.; Goerigk, L. The nonlocal kernel in van der Waals density functionals as an additive correction: An extensive analysis with special emphasis on the B97M-V and ω B97M-V approaches. *J. Chem. Theory Comput.* **2018**, *14*, 5725–5738.

[29] Weigend, F.; Kattannek, M.; Ahlrichs, R. Approximated electron repulsion integrals: Cholesky decomposition versus resolution of the identity methods. *J. Chem. Phys.* **2009**, *130*, 164106.

[30] Nottoli, M.; Jurinovich, S.; Cupellini, L.; Gardiner, A. T.; Cogdell, R.; Mennucci, B. The role of charge-transfer states in the spectral tuning of antenna complexes of purple bacteria. *Photosynth. Res.* **2018**, *137*, 215–226.

[31] Voityuk, A. A.; Rösch, N. Fragment charge difference method for estimating donor–acceptor electronic coupling: Application to DNA π-stacks. *J. Chem. Phys.* **2002**, *117*, 5607–5616.

[32] Hsu, C.-P.; You, Z.-Q.; Chen, H.-C. Characterization of the short-range couplings in excitation energy transfer. *J. Phys. Chem. C* **2008**, *112*, 1204–1212.

[33] Wang, Y.-C.; Feng, S.; Liang, W.; Zhao, Y. Electronic couplings for photoinduced charge transfer and excitation energy transfer based on fragment particle–hole densities. *J. Phys. Chem. Lett.* **2021**, *12*, 1032–1039.

[34] Lee, S.-J.; Chen, H.-C.; You, Z.-Q.; Liu, K.-L.; Chow, T. J.; Chen, I.-C.; Hsu, C.-P. Theoretical characterization of photoinduced electron transfer in rigidly linked donor–acceptor molecules: the fragment charge difference and the generalized Mulliken–Hush schemes. *Mol. Phys.* **2010**, *108*, 2775–2789.

[35] Yang, C.-H.; Hsu, C.-P. A multi-state fragment charge difference approach for diabatic states in electron transfer: Extension and automation. *J. Chem. Phys.* **2013**, *139*, 154104.

[36] Green, J. A.; Asha, H.; Santoro, F.; Improta, R. Excitonic model for strongly coupled multichromophoric systems: the electronic circular dichroism spectra of guanine quadruplexes as test cases. *J. Chem. Theory Comput.* **2020**, *17*, 405–415.

[37] Voityuk, A. A. Electronic couplings for photoinduced electron transfer and excitation energy transfer computed using excited states of noninteracting molecules. *J. Phys. Chem. A* **2017**, *121*, 5414–5419.

[38] Adolphs, J.; Renger, T. How proteins trigger excitation energy transfer in the FMO complex of green sulfur bacteria. *Biophys. J.* **2006**, *91*, 2778–2797.

[39] Renger, T.; Müh, F. Theory of excitonic couplings in dielectric media. *Photosynth. Res.* **2012**, *111*, 47–52.

[40] Renger, T.; Madjet, M. E.; Müh, F.; Trostmann, I.; Schmitt, F.-J.; Theiss, C.; Paulsen, H.; Eichler, H.-J.; Knorr, A.; Renger, G. Thermally activated superradiance and intersystem crossing in the water-soluble chlorophyll binding protein. *J. Phys. Chem. B* **2009**, *113*, 9948–9957.

[41] Epifanovsky, E. et al. Software for the frontiers of quantum chemistry: An overview of developments in the Q-Chem 5 package. *J. Chem. Phys.* **2021**, *155*, 084801.

[42] Lindorfer, D.; Müh, F.; Renger, T. Origin of non-conservative circular dichroism of the CP29 antenna complex of photosystem II. *Phys. Chem. Chem. Phys.* **2017**, *19*, 7524–7536.

[43] Knox, R. S.; Spring, B. Q. Dipole strengths in the chlorophylls. *Photochem. Photobiol* **2003**, *77*, 497–501.

[44] Reimers, J. R.; Cai, Z.-L.; Kobayashi, R.; Rätsep, M.; Freiberg, A.; Krausz, E. Assignment of the Q-Bands of the chlorophylls: Coherence loss via Q_x-Q_y mixing. *Sci. Rep.* **2013**, *3*, 2761.

[45] Friedl, C.; Fedorov, D. G.; Renger, T. Towards a quantitative description of excitonic couplings in photosynthetic pigment–protein complexes: quantum chemistry driven multiscale approaches. *Phys. Chem. Chem. Phys.* **2022**, *24*, 5014–5038.

[46] Raszewski, G.; Diner, B. A.; Schlodder, E.; Renger, T. Spectroscopic properties of reaction center pigments in photosystem II core complexes: Revision of the multimer model. *Biophys. J.* **2008**, *95*, 105–119.

[47] Kabsch, W. A discussion of the solution for the best rotation to relate two sets of vectors. *Acta Crystallogr.* **1978**, *34*, 827–828.

[48] Lindorfer, D.; Renger, T. Theory of anisotropic circular dichroism of excitonically coupled systems: Application to the baseplate of green sulfur bacteria. *J. Phys. Chem. B.* **2018**, *122*, 2747–2756.

[49] Renger, T.; Marcus, R. On the relation of protein dynamics and exciton relaxation in pigment–protein complexes: an estimation of the spectral density and a theory for the calculation of optical spectra. *J. Chem. Phys.* **2002**, *116*, 9997–10019.

[50] Dinh, T.-C.; Renger, T. Towards an exact theory of linear absorbance and circular dichroism of pigment-protein complexes: Importance of non-secular contributions. *J. Chem. Phys.* **2015**, *142*, 01B615_1.

[51] Raszewski, G.; Saenger, W.; Renger, T. Theory of optical spectra of photosystem II reaction centers: location of the triplet state and the identity of the primary electron donor. *Biophys. J.* **2005**, *88*, 986–998.

[52] Renger, T.; Klinger, A.; Steinecker, F.; Schmidt am Busch, M.; Numata, J.; Müh, F. Normal mode analysis of the spectral density of the Fenna–Matthews–Olson light-harvesting protein: how the protein dissipates the excess energy of excitons. *J. Phys. Chem. B.* **2012**, *116*, 14565–14580.

[53] Klinger, A.; Lindorfer, D.; Müh, F.; Renger, T. Normal mode analysis of spectral density of FMO trimers: Intra- and intermonomer energy transfer. *J. Chem. Phys.* **2020**, *153*, 215103.

[54] Bernadotte, S.; Atkins, A. J.; Jacob, C. R. Origin-independent calculation of quadrupole intensities in X-ray spectroscopy. *J. Chem. Phys.* **2012**, *137*, 204106.

[55] May, V.; Kühn, O. *Charge and energy transfer dynamics in molecular systems*; John Wiley & Sons, 2008.

[56] Andrews, S. S. Using rotational averaging to calculate the bulk response of isotropic and anisotropic samples from molecular parameters. *J. Chem. Educ.* **2004**, *81*, 877.

[57] Rosenfeld, L. Quantenmechanische Theorie der natürlichen optischen Aktivität von Flüssigkeiten und Gasen. *J. Phys.* **1929**, *52*, 161–174.

[58] Andrews, S. S.; Tretton, J. Physical principles of circular dichroism. *J. Chem. Educ.* **2020**, *97*, 4370–4376.

[59] Parson, W. Modern optical spectroscopy student edition. 2009.

[60] Hall, J.; Picorel, R.; Cox, N.; Purchase, R.; Krausz, E. New perspectives on photosystem II reaction centres. *Aust. J. Chem.* **2020**, *73*, 669–676.

[61] Germano, M.; Shkuropatov, A. Y.; Permentier, H.; Wijn de, R.; Hoff, A. J.; Shuvalov, V. A.; Gorkom van, H. J. Pigment organization and their interactions in reaction centers of photosystem II: Optical spectroscopy at 6 K of reaction centers with modified pheophytin composition. *Biochem.* **2001**, *40*, 11472–11482.
