# Supplementary material for: Short-Range Effects in the Special Pair of Photosystem II Reaction Centers: The Nonconservative Nature of Circular Dichroism
Source: J Phys Chem Lett. 2023 Dec 20;14(51):11758–67. doi: 10.1021/acs.jpclett.3c02693 (PMC10758115; doi:10.1021/acs.jpclett.3c02693)
Supplement: Supplementary file 6 — jz3c02693_si_006.pdf [file jz3c02693_si_006.pdf]

jz-2023-02693k.R1

Name: Peer Review Information for "Short-Range Effects in the Special Pair of Photosystem II Reaction Centers: The Non-Conservative Nature of Circular Dichroism"

First Round of Reviewer Comments

Reviewer: 1

Comments to the Author

In this manuscript, Gemeinhardt et al. investigate the CD spectrum in the reaction centers of Photosystem II by quantum chemical calculations. As suggested by earlier model calculations, the nonconservative nature of the CD in the Qy region can be related to the short-range couplings in the special pair and with the coupling between Qy and other transitions. Here the authors first build a Hamiltonian for the special pair based on MD simulations, QM calculations and a diabatization method. Then, they embed this Hamiltonian together with the rest of the RC pigments in order to compute the CD, and find a good agreement with experiments. Finally, they reduce the complexity of the model to understand which parameters actually determine the CD shape. They conclude that (i) coupling to CT states enhanced the effective excitonic coupling between the special pair Chls, and (ii) the "electron exchange" between the two Chls affects the direction of their transition dipole moments, thereby changing the shape of the CD.

The main advancement of this work is that it can provide a relationship between microscopic parameters, including CT energies and couplings, and the directly measurable CD spectrum. This opens the possibility of using spectroscopic measurements to derive or validate short-range excitonic (or excitonic-CT) effects. Furthermore, it suggests that the nonconservativity in the CD spectrum arises from the special pair itself, whereas the other pigments are inessential. This provides a link between the nonconservativity of CD spectra and short-range effects in pigment-protein complexes.

This work is well conducted and with several checks for possible sources of error in the calculations. I think the authors did a great job with the calculations and subsequent analysis. I have some doubts though on the presentation of the results and on the definition of electron exchange effects. There are a few points that need to be addressed to help readers understand the authors' reasoning. Overall, I recommend publication in J. Phys. Chem. Letters, provided that the following issues are satisfactorily addressed.

Main points:

1) The authors used a quite complicated protocol, which includes taking some Hamiltonian parameters from quantum chemical calculations which were performed along a MD simulation. Furthermore, they post-process these results in several ways, by "merging" the CT contributions perturbatively in the non-CT Hamiltonian and also by scaling some coupling values. It is quite difficult to follow the entire protocol. I suggest adding a scheme in the SI to better illustrate the calculation protocol, clarifying all the steps (see also the following points). The authors may also want to simplify the presentation of the minimal model.

2) I do not understand the definition of electron exchange used in this paper. The "additional effects" mentioned on page 2, left, line 50 are not detailed, and it is not clear from Ref 5 what is considered as electron exchange. To the best of my understanding, any difference between monomer states and dimer (diabatic) states is chalked up to electron exchange. However, the term electron exchange has a specific physical meaning, i.e. the exchange integral in Hartree-Fock theory. However, a dimer calculation differs from the monomer calculations also in the mutual polarization between the two moieties, both in the ground state and in the excited state. Mutual polarization cannot be entirely represented by a point-charge embedding. In addition, there are repulsion (or penetration) contributions that arise when two molecules are in close contact, whose effect on couplings may be more important (Scholes and Ghiggino, J. Phys. Chem. 1994, 98, 4580-4590). These effects arise from the fact that electron densities have to distort to avoid violating the Pauli exclusion principle. These are significant effects, which in the present calculations cannot be separated from the exchange contribution.

The main effect of "electron exchange" seems to be that on the electric TDMs. Judging from SI Figure 18, this effect seems to be significant also for quite large separations between PD1 and PD2, which is in contrast with the statement that it is an exchange effect. The authors should change the conclusion (ii) "intra special pair electron exchange changes the direction ..." to avoid referring to this effect as "electron exchange".

3) I could not find the energies of the CT states and the couplings between Qy and CT states as calculated by the diabatization. These ingredients are important because they are used in the perturbative correction to determine the CT-mediated effective excitonic coupling. Furthermore, a strong point of this work is the relation between CT mixing and spectroscopic results: the important parameters (CT energies and couplings) should be reported and discussed. Furthermore, the perturbative

corrections in eqs 2 and 3 are valid if the energy differences Qy-CT are large enough.

Other points:

4) It is not clear how the different snapshots from the MD were used. It seems that only one representative structure is used to compute the special pair Hamiltonian, but then in the SI section 4.2

more structures (110 snapshots) are used to estimate the static disorder. So are the values reported on page 5 of the main text obtained from one structure or as the mean over 110 structures? If a single structure is used, do the energies well approximate the average over the 110 MD structures? The authors should also discuss the uncertainty in the parameters obtained from MD.

6) In eqs. 10 and 12 the magnetic/electric transition dipole moments of the 0->Qy transition are corrected for the mixing to CT states. However, for a pure CT state, the transition density is negligible, so the electric transition dipole moment should be negligible as well. Have the authors verified that the obtained diabatic CT states are actual CT states? This analysis should be possible by computing the natural transition orbitals (NTOs). If these effects are indeed negligible, the presentation of the results may be even simplified.

5) If I understood correctly, the minimal model is a "normal" Qy-only exciton model, except that (i) magnetic transition dipole moments are considered for the special pair, and (ii) the couplings, energies, and magnetic/electric dipoles within the special pair are taken from the dimer calculations plus diabatization and include the effect of CT states with eqs 10 and 12. If this is correct, I suggest the authors to simplify the presentation of this model on page 8 (right).

7) Page 8, right, lines 15-20, "(v) neglecting the impact of higher LE states on the Qy transition" Does point (v) refer to higher LE states of the special pair Chls, or also to those of the other pigments? For example, are the excited states of the carotenes included?

8) Although the agreement in Figure 2 with experiments is very good, there is an important discrepancy. Both experiments show a positive shoulder around 672 nm between the main positive band and the negative band, which is not reproduced by calculations. Could this band arise from a different conformation of the complex, since (as mentioned by the authors) the short-range effects are sensitive to the conformations?

9) Figure 15 in the SI: It would be useful to swap the sign of the normal vector, so that the angle of the magnetic TDMs can be gauged more easily.

10) Page 10, right, lines 30-33: the reference to SI Figure 7 seems out of place here.

Reviewer: 2

Comments to the Author

The manuscript reports a multiscale approach that attempts to elucidate the nature of excitonic coupling in the special pair of photosystem II. The protocol appears intricate, combining various pieces of information and different techniques and approximations of theoretical analysis. A major conclusion of the manuscript is that the coupling to CT states is responsible for almost half of the excitonic coupling in the special pair, and that remaining short-range effects cause the non-conservative nature of the CD spectrum via the mechanism discussed in the manuscript.

1) I have no doubt that this is a significant addition to the literature in the field. I feel however that at least the presentation should be improved in order to make this work more accessible and comprehensible. Often the text is quite cramped, dense and technical, while at the same time relying extensively on the SI. I count 16 references to SI material, which to me feels excessive. It would be extremely beneficial if the authors make an effort to simplify, clarify, and try to focus the content by being more selective in what should be covered. If all the points covered in this work are indeed judged to be essential, then perhaps a longer full paper would be more appropriate, where the authors can take the space to explain things in detail and structure their presentation without space constraints.

2) Since this work moves on from the Lindorfer et al. 2021 paper (ref. 34), there should be a clear discussion at some point about specific aspects that are different from that work, and how the reader should think about these two papers and their conclusions.

3) Given the multi-layered nature of the approach, it would be useful to include overview comments about which components or assumptions may represent the weakest links or be associated with the largest uncertainties.

4) TD-DFT calculations were performed, but unless I am mistaken I did not see a full report of the results and an analysis of the nature of excited states. This is particularly relevant for the CT states. As far as I know, all quantum chemical studies so far (either in vacuo or embedded, e.g. Ref. 32) have placed CT states very high in energy for the PSII special pair. It is important to know what the calculations in this work suggest about such CT states and how the authors discuss their conclusion on the extensive coupling to CT states if these CT states are computed to be many thousands of wavenumbers higher than local excitations.

Author's Response to Peer Review Comments:

Dear Prof. Editor,

many thanks for chosing such expert reviewers and for sending their reports. We thank the reviewers for their constructive and insightful reviews, which were very helpful for us to improve our manuscript. Please find below a detailed response to the reviews and a description of the respective changes in the revised version of our manuscript. Please note that the changes in the manuscript have been marked in blue in the file “marked changes manuscipt.pdf”. In addition, we repeat the respective changes from the revised manuscript below (in blue color).

We hope that our manuscript can be published in its present form.

Yours sincerely,
Felix Gemeinhardt and Thomas Renger, in the name of all co-authors.

Review #1

Reviewer:
In this manuscript, Gemeinhardt et al. investigate the CD spectrum in the reaction centers of Photosystem II by quantum chemical calculations. As suggested by earlier model calculations, the nonconservative nature of the CD in the Qy region can be related to the short-range couplings in the special pair and with the coupling between Qy and other transitions. Here the authors first build a Hamiltonian for the special pair based on MD simulations, QM calculations and a diabatization method. Then, they embed this Hamiltonian together with the rest of the RC pigments in order to compute the CD, and find a good agreement with experiments. Finally, they reduce the complexity of the model to understand which parameters actually determine the CD shape. They conclude that (i) coupling to CT states enhanced the effective excitonic coupling between the special pair Chls, and (ii) the ”electron exchange” between the two Chls affects the direction of their transition dipole moments, thereby changing the shape of the CD. The main advancement of this work is that it can provide a relationship between microscopic parameters, including CT energies and couplings, and the directly measurable CD spectrum. This opens the possibility of using spectroscopic measurements to derive or validate short-range excitonic (or excitonic-CT) effects. Furthermore, it suggests that the nonconservativity in the CD spectrum arises from the special pair itself, whereas the other pigments are inessential. This provides a link between the nonconservativity of CD spectra and short-range effects in pigment-protein complexes. This work is well conducted and with several checks for possible sources of error in the calculations. I think the authors did a great job with the calculations and subsequent analysis. I have some doubts though on the presentation of the results and on the definition of electron exchange effects. There are a few points that need to be addressed to help readers understand the authors’ reasoning. Overall, I recommend publication in J. Phys. Chem. Letters, provided that the following issues are satisfactorily addressed.

Our answer: *We thank the reviewer for reading our manuscript so carefully and for appreciating our work.*

Reviewer:

(R1.1) 1) The authors used a quite complicated protocol, which includes taking some Hamiltonian parameters from quantum chemical calculations which were performed along a MD simulation. Furthermore, they post-process these results in several ways, by ”merging” the CT contributions perturbatively in the non-CT Hamiltonian and also by scaling some coupling values. It is quite difficult to follow the entire protocol. I suggest adding a scheme in the SI to better illustrate the calculation protocol, clarifying all the steps (see also the following points). The authors may also want to simplify the presentation of the minimal model.

Our answer:

We appreciate the point of the reviewer to provide an overview on the applied methodology and provide an according scheme together with textual explanations in the SI (Section 1). We refer to this section in the main text on page 3, right column, bottom.:

“A schematic overview of the applied methodological procedure is given in the SI, Section 1, and further details are provided in the SI, Sections 2 and 3.”

Regarding the presentation of the minimal model, we kindly refer to our answer to (R1.6).

Reviewer:

(R1.2) 2) I do not understand the definition of electron exchange used in this paper. The ”additional effects” mentioned on page 2, left, line 50 are not de- tailed, and it is not clear from Ref 5 what is considered as electron exchange. To the best of my understanding, any difference between monomer states and dimer (diabatic) states is chalked up to electron exchange. However, the term electron exchange has a specific physical meaning, i.e. the exchange integral in Hartree-Fock theory. However, a dimer calculation differs from the monomer calculations also in the mutual polarization between the two moieties, both in the ground state and in the excited state. Mutual polarization cannot be entirely represented by a point-charge embedding. In addition, there are repulsion (or penetration) contributions that arise when two molecules are in close contact, whose effect on couplings may be more important (Scholes and Ghiggino, J. Phys. Chem. 1994, 98, 45804590). These effects arise from the fact that electron densities have to distort to avoid violating the Pauli exclusion principle. These are significant effects, which in the present calculations cannot be separated from the exchange contribution. The main effect of ”electron exchange” seems to be that on the electric TDMs. Judging from SI Figure 18, this effect seems to be significant also for quite large separations between PD1 and PD2, which is in contrast with the statement that it is an exchange effect. The authors should change the conclusion (ii) ”intra special pair electron exchange changes the direction ...” to avoid referring to this effect as ”electron exchange”.

Our answer:

Many thanks for these insights. We completely agree that our diabatization procedure can only separate the CT coupling effects but cannot discriminate between different non-CT short-range effects. We have, therefore changed the term “electron exchange effects” to “non-CT-SR effects” throughout the paper. We have also added a short discussion of the paper by Scholes and Ghiggino together with three related works (refs 9-12) to characterize different possible non-CT-SR effects and their relation to the CT coupling effects, on page 2, left column, bottom/ page 3, right column, top, where we also admit that the present approach cannot differentiate between different non-CTSR effects:

"These SR interactions give rise to a coupling between local excited (LE) and charge transfer (CT) states, [5,6] as well as additional effects, [7] which will be summarized as non-CT-SR contributions. Theoretical studies on simple model systems investigated the relative importance of CT-coupling and the non-CT-SR contributions due to Dexter exchange interaction[8] and orbital penetration[9] to the excitonic coupling. The CT coupling effects were reported to be large compared to non-CT-SR contributions.[10,11] Concerning the latter, except for the case of orthogonal donor and acceptor orbitals,[12] orbital penetration effects were reported to dominate Dexter electron exchange.[9] In the present work we will use a diabatization technique to identify the CT contributions, but we will not be able to discriminate between different non-CT-SR effects."

We also agree that in the distance dependence of the transition dipole angles (SI, Fig. 20) there are changes for larger distances that could be due to mutual polarization effects. We discuss this point on page 11, left column below Fig. 5:

"Increasing the distance between the special pair pigments by moving P_{D2} along the normal on the pigment plane of P_{D1}, the wavefunction overlap is decreased and the orientations of transition dipole moments approach that of the isolated pigments (Fig. S18). The largest changes occur for distance increases between $\Delta r = 0$ (native structure) and $\Delta r = 3$ Å, reflecting the SR effects. The minor changes observed for Δr up to 10 Å show that also small LR effects are present, which could be due to dispersive interactions in the special pair leading to a mutual polarization of LE states."

Reviewer:

(R1.3) 3) I could not find the energies of the CT states and the couplings between Qy and CT states as calculated by the diabatization. These ingredients are important because they are used in the perturbative correction to determine the CT-mediated effective excitonic coupling. Furthermore, a strong point of this work is the relation between CT mixing and spectroscopic results: the important parameters (CT energies and couplings) should be reported and discussed. Furthermore, the perturbative corrections in eqs 2 and 3 are valid if the energy differences Qy-CT are large enough.

Our answer:

We thank the reviewer for highlighting the need for further data regarding the CT-states. We have summarized our findings discussing all the points of the reviewer, on page 4 left column, bottom/right column top, providing reference to the SI, where we provide an excerpt of the relevant parameters of the latter Hamiltonian (SI, Table 4) as well as a more detailed discussion (SI, Section 5.2):

"The lowest energy CT states are about 10000 cm^{-1} above the Q_y states of P_{D1} and P_{D2} and the largest couplings between the Q_y and the CT states are in the order of 1000 cm^{-1} (Table S4), justifying the perturbation theory that will be used below to treat the mixing of Q_y and CT states. From an analysis of the transition density matrix of the special pair, the first two adiabatic states can be characterized as delocalized over the Q_y states of P_{D1} and P_{D2} with a 3-5 % admixture of CT states. The CT states obtained from the diabatization have an actual CT chararacter larger than 82 % (90 % on average). The remaining small percentage of LE character results in non-zero but small transition dipole moments of the diabatic CT states, which, however, have no critical effect on the results, as will be shown below. Further details of the diabatic Hamiltonian parameters are reported and discussed in the SI (Section 5.2)."

Reviewer:

(R1.4) 4) It is not clear how the different snapshots from the MD were used. It seems that only one representative structure is used to compute the special pair Hamiltonian, but then in the SI section 4.2 more structures (110 snapshots) are used to estimate the static disorder. So are the values reported on page 5 of the main text obtained from one structure or as the mean over 110 structures? If a single structure is used, do the energies well approximate the average over the 110 MD structures? The authors should also discuss the uncertainty in the parameters obtained from MD.

Our answer:

We appreciate the reviewer's point regarding a further description of the use of the MD snapshots. The computations, except for determining the static disorder and reliability checks, are based on a single representative structure, which is defined as the snapshot with the lowest RMSD to the average coordinates during the simulation. We have clarified these points on page 3 bottom/page 4 upper part:

"A representative structure of the special pair dimer dimer is selected from the MD simulation as the snapshot, which shows the least root mean square deviation (RMSD) compared to the average backbone configuration of the protein (cf. SI, Section 4). We will use this representative structure and additional 110 MD snapshots (after a QM/MM geometry optimization) for the quantum chemical calculations of SR effects in the special pair. The calculations on the snapshots are needed to obtain disorder parameters for the calculation of inhomogeneous broadening of the spectra and to validate the short-range contributions, obtained for the respresentative structure." *on page 5 left column, lower half/right column upper half:*

"By comparing the diabatic energies with those obtained for the isolated monomers, non-CT-SR contributions to the site energy shifts can be inferred, whereas the second contribution on the r.h.s. of eq 2 reveals the effect of couplings between LE and CT states. This analysis was performed for the representative structure. We have checked the reliability of this structure by comparing energies and couplings with those obtained from an average over 110 geometry-optimized MD snapshots. Interestingly, non-CT-SR effects lead to a 57 cm^{-1} blue-shift of the site energies (averaged over P_{D1} and P_{D2} , Table S5) and the coupling to CT states results in a 158 cm^{-1} red shift (164 cm^{-1} average over the 110 MD snapshots, Table S8), such that the overall SR effect on the site energies is rather small." *on page 5, right column, below eq 3:*

"LR Coulomb interaction of transition densities and non-CT-SR effects are included in [...] for which we obtain a value of 137 cm^{-1} from the diabatization (133 cm^{-1} average over the 110 MD snapshots)." *on page 6, left column, above eq 4:*

"The second contribution on the r.h.s. of eq 3 for a = Q_y arises from superexchange type couplings of Q_y states to CT states in the special pair, which result in a coupling contribution of 98 cm^{-1} (110 cm^{-1} average over 110 MD snapshots)." *and on page 7 right column, middle part, where we also reference to the SI, sections 4.1 and 5.2, and Fig. S6 that contains further details as the distribution functions:*

"For the special pair pigments, it can be expected that SR effects on site energies and couplings are particularly sensitive with respect to conformational changes of the complex. Therefore, the width of the distribution of every element of the diabatic Hamiltonian of the special pair has been estimated by considering 110 geometry-optimized snapshots of the MD simulations, as discussed in more detail below and in the SI (Sections 4.1 and 5.2, Fig. S6)."

Reviewer:
(R1.5) 6) In eqs. 10 and 12 the magnetic/electric transition dipole moments of the 0− >Qy transition are corrected for the mixing to CT states. However, for a pure CT state, the transition density is negligible, so the electric transition dipole moment should be negligible as well. Have the authors verified that the obtained diabatic CT states are actual CT states? This analysis should be possible by computing the natural transition orbitals (NTOs). If these effects are indeed negligible, the presentation of the results may be even simplified.

Our answer:
We thank the reviewer for the point and kindly refer to our answer to (R1.3) and, in addition, quantify and discuss these aspects on page 6, right column bottom/page 7, left column, top:
"Please note that the CT states, obtained in our diabatization procedure, have small but nonzero transition dipole moments, as discussed above. By treating the CT states in the above perturbation theory, it is possible to circumvent the problem of a proper pigment center definition for the CT states, which would be needed, if the CT states were explicitly included in eq 6."

Reviewer:
(R1.6) 5) If I understood correctly, the minimal model is a "normal" Qy-only exciton model, except that (i) magnetic transition dipole moments are considered for the special pair, and (ii) the couplings, energies, and magnetic/electric dipoles within the special pair are taken from the dimer calculations plus diabatization and include the effect of CT states with eqs 10 and 12. If this is correct, I suggest the authors to simplify the presentation of this model on page 8 (right).

Our answer:
We thank the reviewer for this suggestion and have changed the presentation of the minimal model accordingly, on page 9 left column, middle part:
"We finally arrive at an effective Q_y Hamiltonian of the RC reading […] which resembles the one of the Q_y-only model, discussed above, except for the following adaptations concerning the special pair pigments: (i) an increased excitonic coupling of 150 cm^{-1} that takes into account CT state coupling effects, and (ii) enhanced static disorder, described by an increased width (FWHM) of
280 cm^{-1} of the distribution function of the site energies E_{PD1} and E_{PD2}, as compared to 180 cm^{-1} (FWHM) used for [...] . In addition, our minimal model takes into account (iii) rotated electric and magnetic transition dipole moments of the special pair pigments, taking into account SR effects (eqs 9 and 11, respectively) and (iv) the resulting intrinsic contribution to the CD signal (eq 7). Please note, that site energies of the RC pigments proposed earlier[31,36] are used."

Reviewer:
(R1.7) 7) Page 8, right, lines 15-20, "(v) neglecting the impact of higher LE states on the Qy transition" Does point (v) refer to higher LE states of the special pair Chls, or also to those of the other pigments? For example, are the excited states of the carotenes included?

Our answer:
The point (v) refers to all pigments of the reaction center. Carotenoids are not included in our minimal model. These points are clarified by presenting the minimal model, as the reviewer suggested (see point R1.6 and our answer above).

Reviewer:
(R1.8) 8) Although the agreement in Figure 2 with experiments is very good, there is an important discrepancy. Both experiments show a positive shoulder around 672 nm between the main positive band and the negative band, which is not reproduced by calculations. Could this band arise from a different conformation of the complex, since (as mentioned by the authors) the short-range effects are sensitive to the conformations?

Our answer:
We appreciate this valuable point of the reviewer. We discuss this idea on page 7, right column, bottom/page 8, left column, below Fig. 2:
"A notable deviation between theory and experiment seems is the theoretical underrepresentation of the shoulder occuring in both CD experiments (with different magnitude) around 673 nm. Among other uncertainties, it could be that this shoulder reflects a conformational substate not present in our calculations, which reveal unimodal distribution functions for energies and couplings (Fig. S8). In our calculations of optical spectra, these distribution functions are approximated by single Gaussian functions. The differences between experimental CD spectra could be related to different sample preparations giving rise to slightly different conformations that are sensed by the SR effects in the CD spectrum." *and in the outlook on page 11, right column, lower half/ page 12 top:*
"An open point of the present work concerns the temporal characterization of conformational dynamics by QM/MM simulations. So far, we have attributed the enhancement of site energy fluctuations by CT state couplings to static disorder. However, it will be more realistic to include the fast fluctuations in the spectral density of the exciton-vibrational coupling, leading to stronger homogeneous broadening that could lead to different overlap effects between optical lines in the CD spectrum influencing the apparent non-conservativity ratio. A related point concerns the modeling of solubilized complexes (including their detergent belts) that could reveal information on possible conformational substates probed by the optical experiments on such samples. We note that the detergent has an influence on SR-effects in the special pair of bRC due to a shift of conformational equilibria.[62]"

Reviewer:
(R1.9) 9) Figure 15 in the SI: It would be useful to swap the sign of the normal vector, so that the angle of the magnetic TDMs can be gauged more easily.

Our answer:
When swapping the sign of the normal vector, we have seen strong overlaps with the dipole moments. Therefore, we decided to keep the current sign for gauging the angle.

Reveiwer:
R1.10) 10) Page 10, right, lines 30-33: the reference to SI Figure 7 seems out of place here.

Our answer:

Thanks, this was indeed a mistake. The correct reference is Figs. S15 and S16, as written now on page 9, right column, bottom:

"We note in passing that despite the stronger influence of non-CT-SR effects on the electric than on the magnetic transition dipole moment of the Q_y transition of the P_{D1} and P_{D2} pigments, for the higher excited states the magnetic transition dipole moments are affected stronger than the electric ones (SI, Section 8, Figs. S15 & S16). These higher excited state transition dipole moments, however, have practically no effect on the spectra in the Q_y spectral region, as our minimal model demonstrates."

Review #2

Reviewer:
The manuscript reports a multiscale approach that attempts to elucidate the nature of excitonic coupling in the special pair of photosystem II. The protocol appears intricate, combining various pieces of information and different techniques and approximations of theoretical analysis. A major conclusion of the manuscript is that the coupling to CT states is responsible for almost half of the excitonic coupling in the special pair, and that remaining short-range effects cause the nonconservative nature of the CD spectrum via the mechanism discussed in the manuscript.

Our answer:
We thank the reviewer for pointing out the two main findings of our paper.

Reviewer:
(R2.1) 1) I have no doubt that this is a significant addition to the literature in the field. I feel however that at least the presentation should be improved in order to make this work more accessible and comprehensible. Often the text is quite cramped, dense and technical, while at the same time relying extensively on the SI. I count 16 references to SI material, which to me feels excessive. It would be extremely beneficial if the authors make an effort to simplify, clarify, and try to focus the content by being more selective in what should be covered. If all the points covered in this work are indeed judged to be essential, then perhaps a longer full paper would be more appropriate, where the authors can take the space to explain things in detail and structure their presentation without space constraints.

Our answer:
We thank the reviewer for pointing out that our presentation was not clear enough. We have rewritten the paper and refer to the SI only as additional source of information, not needed to get an overview. We have shortened the description of static disorder models (on page 8, right column, lower half), removing one Table. We have simplified the description of the minimal model, as decribed in our answer to point R1.6 of reviewer one (see above) and we also simplified the theory part by rearranging the description of electric and magnetic transition dipole moments on page 6 right column/page 7 left column. We also have restructered and simplified the supporting information.

Reviewer:
(R2.2) 2) Since this work moves on from the Lindorfer et al. 2021 paper (ref. 634), there should be a clear discussion at some point about specific aspects that are different from that work, and how the reader should think about these two papers and their conclusions.

Our answer:
*We appreciate the concern of the reviewer regarding the discussion of our previous work. The differences and corresponding conclusions have already been mentioned in the main paper and the SI of the original version (Section 5.2). However, we reevaluated our original presentation in this regard and added some clarifying text in the main paper, on page 3, right column, middle part: "*A main goal of the present work has been to investigate this hypothesis by explicitly taking into account the coupling between LE and CT states. As will be shown here, besides

coupling to CT states, there are other more dominant SR effects at play that determine the non-conservative nature of the CD spectrum." *and on page 11, left column bottom:*
"We finally want to come back to our previous model,[47] where, without including SR effects on the transition dipole moments of the special pair, but including the coupling between LE Q_y states and higher LE states of the RC pigments, a non-conservativity ratio of R = 2.3 was obtained,[47] that could be increased to R = 2.9 by assuming a three-fold enhanced excitonic coupling between Q_y and high-energy LE states in the special pair. It was proposed that the enhancement could occur due to superexchange via the CT states. As noted above, this effect could not be found in the present calculations. In order to clarify the remaining discrepancy between our previous and the present work, we applied the same methodology as before[47] and obtained R = 1.4. The remaining discrepancy to the earlier value of R = 2.3 is caused by the choice and calculation of special pair parameters (LE state energies, couplings, and transition dipole moments) as explained in the SI (Section 6.2). Obviously, the coupling to high-energy transitions in the case of PSII RCs is not the main contributor to the non-conservativity of the CD spectrum, in contrast to other pigmentprotein complexes.[45,46]"

Reviewer:
(R2.3) 3) Given the multi-layered nature of the approach, it would be useful to include overview comments about which components or assumptions may represent the weakest links or be associated with the largest uncertainties.

Our answer:
We added an overview figure in the SI (section 1), where the different layers of our approach are summarized. We have tried to balance these components such that there is no obvious weakest link. Most likely, our modeling of the sample, on which the optical experiments are performed, could be improved and we could distinguish between static and dynamic disorder in order to obtain more realistic optical lineshapes. Please see point R1.8 and our answer above.

Reviewer:
(R2.3) 3) 4) TD-DFT calculations were performed, but unless I am mistaken I did not see a full report of the results and an analysis of the nature of excited states. This is particularly relevant for the CT states. As far as I know, all quantum chemical studies so far (either in vacuo or embedded, e.g. Ref. 32) have placed CT states very high in energy for the PSII special pair. It is important to know what the calculations in this work suggest about such CT states and how the authors discuss their conclusion on the extensive coupling to CT states if these CT states are computed to be many thousands of wavenumbers higher than local excitations.

Our answer:
We appreciate the point of the reviewer and note, that a similar issue has been raised by reviewer 1.
Thus, we kindly refer to our answers to (R1.3) and (R1.5) above in this regard.
